# Biosecurity measures reducing *Salmonella* spp. and hepatitis E virus prevalence in pig farms—a systematic review and meta-analysis

**DOI:** 10.3389/fvets.2024.1494870

**Published:** 2024-12-23

**Authors:** Nikolaus Huber, Marina Meester, Elena L. Sassu, Elisabeth S. L. Waller, Gergana Krumova-Valcheva, Giuseppe Aprea, Daniela D’Angelantonio, Veit Zoche-Golob, Silvia Scattolini, Emily Marriott, Richard P. Smith, Elke Burow, Guido Correia Carreira

**Affiliations:** ^1^Unit of Veterinary Public Health and Epidemiology, Institute of Food Safety, Food Technology and Veterinary Public Health, University of Veterinary Medicine, Vienna, Austria; ^2^Department of Population Health Sciences, Faculty of Veterinary Medicine, Utrecht University, Utrecht, Netherlands; ^3^Division for Animal Health, Austrian Agency for Health and Food Safety (AGES), Vienna, Austria; ^4^Department of Epidemiological Sciences, Animal Plant and Health Agency, Weybridge, United Kingdom; ^5^National Food Safety Centre, National Diagnostic and Research Veterinary Medical Institute, Sofia, Bulgaria; ^6^Department of Food Hygiene, Istituto Zooprofilattico Sperimentale dell’Abruzzo e del Molise “G. Caporale”, Teramo, Italy; ^7^Department of Biological Safety, German Federal Institute for Risk Assessment (BfR), Berlin, Germany; ^8^Department of Rural Development and Agriculture, Ministry of Agriculture, Environment and Climate Protection of the State of Brandenburg, Potsdam, Germany

**Keywords:** biosecurity, swine herds, interventions, zoonoses, meta-analysis, risk reduction, HEV, *Salmonella* spp.

## Abstract

*Salmonella* spp. and hepatitis E virus (HEV) are significant foodborne zoonotic pathogens that impact the health of livestock, farmers, and the general public. This study aimed to identify biosecurity measures (BSMs) against these pathogens on swine farms in Europe, the United States, and Canada. Overall, 1,529 articles from three scientific databases were screened manually and with the artificial intelligence (AI) tool ASReview. We identified 54 BSMs from 32 articles, primarily focused on *Salmonella* spp. control. Amongst the extracted BSMs, only five measures for *Salmonella* spp. control, namely, ‘acidification of feed’, ‘acidification of drinking water’, ‘rodent control’, ‘all-in and all-out production’, and ‘disinfection’ had sufficient observations to conduct a meta-analysis. Of these five, acidification and rodent control were found to be protective measures, that is, their summary odds ratios in the corresponding meta-analyses were lower than 1, indicating lower odds of *Salmonella* spp. presence on farms which implemented these BSM compared to farms which did not implement them (odds ratio [OR] around 0.25). All-in and all-out production showed a non-significant protective effect (OR = 0.71), while disinfection showed a statistically non-significant lack of association between disinfection and the presence of *Salmonella* spp. on the farm (OR = 1.03). For HEV, no meta-analysis could be performed. According to multiple articles, two BSMs were significantly associated with a lower risk of HEV presence, namely, disinfecting vehicles (OR = 0.30) and quarantining pigs before introducing them on the farm (OR = 0.48). A risk of bias assessment for each included article revealed a high risk in the majority of the articles, mainly due to selection and performance bias. This emphasises the lack of standardised, high-quality study designs and robust empirical evidence linking BSM implementation to pathogen reduction. The limited data available for meta-analysis, coupled with the high risk of bias (RoB) in the literature, highlights the urgent need for more substantial evidence on the effectiveness of BSMs in mitigating the transmission and spread of zoonotic pathogens, such as *Salmonella* spp. and HEV on pig farms.

## 1 Introduction

### 1.1 Rationale

Livestock-associated zoonotic pathogens are a serious public health threat and an occupational hazard for farmers and personnel who are continuously in contact with farm animals and their products (1). Amongst other pathogens, *Salmonella* spp. and hepatitis E virus (HEV) are zoonotic pathogens that can cause foodborne disease in humans and are difficult to control in pig farms.

Salmonellosis is the second most reported gastrointestinal infection in humans after campylobacteriosis. In 2021, there were 60,050 human cases of salmonellosis in the European Union (EU), of which 6,755 were cases associated with foodborne outbreaks. In particular, the most common vehicle for *Salmonella* spp. included ‘pig meat and products thereof” (European Food Safety Authority [EFSA] and European Centre for Disease Prevention and Control [ECDC]) (2). In 2018, the United States Department of Agriculture (USDA) estimated the annual economic cost of human illness due to foodborne salmonellosis alone was $4.1 billion (USDA) (3). There is, therefore, a strong incentive to control *Salmonella* spp. in the pig food chain both from a healthcare and an economic perspective.

Similar to *Salmonella* spp. infections, HEV infections are subclinical in pigs. Pigs may be infected with HEV genotype 3 or 4 and shed the virus in faeces. Seroprevalences of up to 93% (4) have been reported in individual pigs and on the farm level, with up to 100% of farms being affected by HEV in European countries (5, 6). Humans can become infected by consuming raw or undercooked pork, particularly pork liver. In industrialised countries, pigs are presumed to be a significant source of human HEV infection (EFSA) (7). Contrary to pigs, HEV infection in humans may cause acute hepatitis, and in immunocompromised patients, can result in acute or acute-to-chronic liver failure and even become fatal (8), with 44,000 fatalities recorded worldwide in 2015 [WHO (9)].

Current policies emphasize prevention-based food production systems to address pathogens, such as *Salmonella* spp., and HEV and ensure food safety. Vora et al. (10) identified on-farm biosecurity measures (BSMs) as crucial to preventing zoonotic transmission from livestock to humans. A BSM is defined as “the implementation of a segregation, hygiene, or management procedure (excluding medically effective feed additives and preventive/curative treatment of animals) that specifically aims at reducing the probability of the introduction, establishment, survival, or spread of any potential pathogen to, within, or from a farm, operation or geographical area” (11). Implementing BSMs in swine production can reduce pathogen levels from farm to fork, leading to less contaminated pork and lower foodborne outbreak risks (12). Thus, the knowledge of effective BSMs on pig farms is vital for animal health, public health, and economic reasons.

Numerous types of BSMs in pig farming have been described in the scientific literature either in general or specifically against *Salmonella* spp. or HEV infections (13–15). One recent systematic review studied animal health/veterinary interventions’ effectiveness against pathogens relevant to pig herds and found feed additives and vaccination were appropriate for *Salmonella* spp. control, but not for HEV (16). Nevertheless, the effectiveness of such measures in reducing the risk of pathogen introduction or spread on or between farms has not yet been systematically reviewed and quantified comprehensively.

To our knowledge, this systematic review represents the first attempt to collate the published literature on BSMs against *Salmonella* spp. and HEV infections in domestic pigs from different exploratory study designs, analyse their effectiveness in reducing the prevalence of these pathogens at the farm level, and assess the eligible scientific literature regarding a potential risk of bias.

### 1.2 Aims and objectives

The primary aims of this review were to (i) provide an overview of the existing literature on BSMs for the control of *Salmonella* spp. and HEV infections at different production stages of domestic pigs on farms and (ii) outline the quantitative effectiveness of these BSMs. The secondary aim was to identify areas where future research is needed.

### 1.3 Research question

The research question was framed according to the Population, Intervention, Comparison, and Outcome (PICO) format.

P (Population): Swine farms in the EU, UK, USA, and Canada.

I (Intervention): Biosecurity measures (e.g., acidification, rodent control).

C (Comparison): Farms that did not implement these biosecurity measures.

O (Outcome): Reduction in prevalence of *Salmonella* spp. or HEV.

## 2 Methods

### 2.1 Systematic literature search

The outcome investigated in this study was the quantitative effectiveness of BSMs in reducing the occurrence of *Salmonella* spp. and HEV in pig farms. The geographical regions of interest were the United States, Canada and Europe and its associated political territories (e.g., French territories overseas which—although having a different climate than the US/Canada/Europe—are still subject to EU legislation on pathogen control). The decision to restrict the study to the EU, USA, Canada, and the UK was based on the socioeconomic similarities and established biosecurity frameworks that facilitated a more comparable analysis. The inclusion and exclusion criteria used to select studies for this systematic review are displayed in Table 1.

**Table 1:** Eligibility criteria for the searching and screening of the literature.

Category	Inclusion criteria	Exclusion criteria
Location	European Union (EU) and associated political territories (EU)	Any countries outside of the EU/UK/USA/Canada
	United Kingdom (UK)	
	United States of America (USA)	
	Canada	
Design	Peer-reviewed journal articles	Reviews
	Observational and Experimental studies	Systematic analyses
		Qualitative research articles
		Conference proceedings
Content	Primary^*^/external biosecurity measures (preventing spread between farms)	Tertiary^***^ biosecurity measures (increase resistance or immunity of the animals against pathogens). Only feed and water additives, that is, organic acids, were included.
	Secondary^**^/internal biosecurity measures (preventing spread within farms) Use of feed and water additives	Any biosecurity measures which were being applied in the slaughterhouse (i.e., not on the farm).
Species	Domestic pigs (*Sus scrofa domesticus*)	Wild boar (*Sus scrofa*) or any other species
Languages	English, French, Spanish, German, Dutch, Bulgarian, and Italian	Studies not understandable to at least one author in the team

* Reduction of the introduction and spread of microorganisms between farms.

** Reduction of the transmission or spread within a farm.

*** Increasing the ability of the animals to cope with the pathogens. Only one pathogen was searched simultaneously in conjunction with the rest of the search query terms.

**Figure 1:**
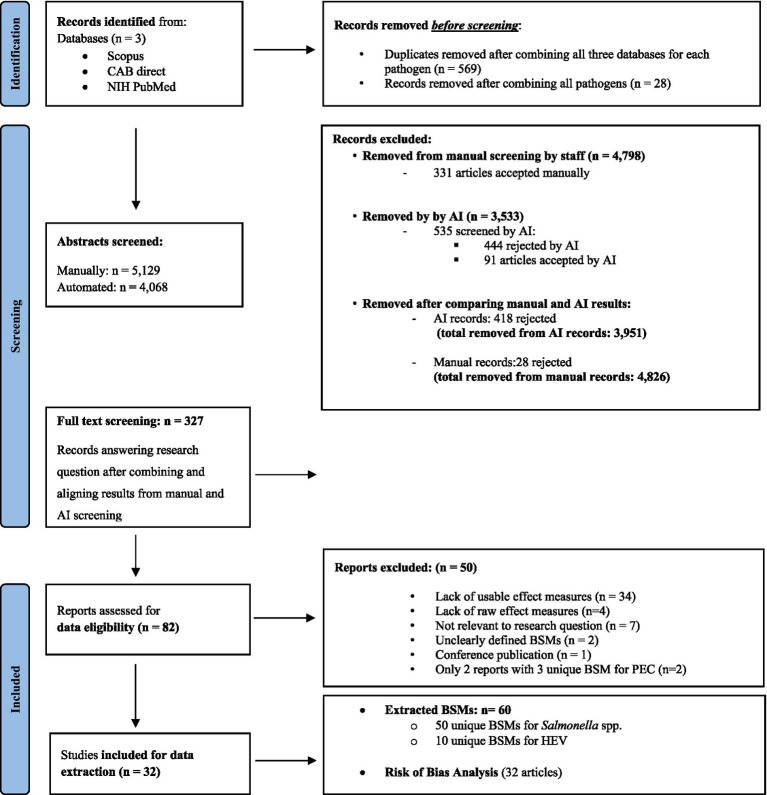
Preferred Reporting Items for Systematic Reviews and Meta-Analyses (PRISMA) flow diagram showing the workflow of the systematic review. Source: Adapted from reference (71). 35 articles made it to the final list, but BSMs were only extracted and analysed from 32 articles as one article reported confidence intervals for pathogen being not transformable into confidence intervals (CIs) for an OR and only 2 articles with 3 unique BSMs were identified for PEC and were, therefore, excluded from further analysis.

Initially the purpose of our search included pathogenic *E. coli* (PEC) as well. Since the literature search resulted in only two eligible publications, we excluded PEC from this analysis. However, PEC was still included in the search terms and is documented in the search process, as shown in Figure 1 and Supplementary Table S1.

Three scientific databases were used based on the author’s institutional access to the following databases: Scopus, CAB Direct, and National Institutes of Health (NIH) PubMed. The queries (Supplementary Table S1) were run separately for each of the pathogens but on all the databases, leading to nine searches conducted in April 2021.

A list of three example articles for both HEV and *Salmonella* spp. was created based on expert knowledge about available literature on these pathogens to validate that the search query was picking up appropriate articles. We deemed the query as effective if at least two of the three selected articles were identified.

#### 2.1.1 Data collection process

From each database, a list of search results per pathogen was exported from the Web interface, and the results were merged per pathogen into a Microsoft Excel file, from which duplicates were manually removed. Each article was provided with a unique identifier. Any articles that did not have author metadata were removed from the final list. In total, 2,314 titles were identified through the database search for *Salmonella* spp. (*n* = 2,003) and for HEV (*n* = 311; and 5,129 when including PEC; Figure 1). Titles and abstracts were screened in duplicate: once by one of the 12 researchers during a manual screening round and once in parallel using the AI reviewing software ASReview version 0.18.rc0 ^1^ (17).

#### 2.1.2 Screening process

Articles were initially screened by one of the primary authors according to the criteria in Table 1 and excluded if one of these criteria was not met. After that, they were equally divided amongst the 12 authors for three rounds of selection: (i) title and abstract screening stage, (ii) full-text screening, and (iii) data extraction. During these stages, reviewers were provided with an Excel file containing the details of the articles alongside a list of questions which referred to the review’s eligibility criteria (Table 1), with the article either accepted or rejected at the end of the review. Further details on this process are shown in Figure 1.

#### 2.1.3 ASReview

Screening was also performed using ASReview. This software uses an active learning technique. At first, some articles classified by a researcher as ‘positive’ (i.e., relevant/match selection criteria) and ‘negative’ (i.e., irrelevant/not matching selection criteria) are selected; thus, creating a starting point to classify publications as relevant or not. Then, ASReview starts screening the remaining publications and proposes articles that may be most relevant. The researcher checks each proposed publication and classifies them as relevant or not. ASReview picks up from the relevant and irrelevant articles defined by the researcher and continuously updates its knowledge, proposing further publications most likely to be relevant as well. This process is repeated until ASReview is proposing no more relevant articles, so one may assume that all relevant articles have been selected and the articles left to screen are irrelevant. Therefore, screening can be stopped before all articles are reviewed, thereby improving the efficiency of the reviewing process. Because of its efficiency, a single researcher could act as the “duplicate” reviewer through the software. Articles were re-extracted from the search databases following the same aforementioned query, yet in a format useable for ASReview, and after removing duplicates, 4,068 articles were uploaded to the ASReview software. The positive articles were uploaded separately and consisted of one article per pathogen (18–20). Additionally, three randomly proposed articles by the software that did not meet the selection criteria were selected as negative articles. Subsequently, 536 articles (13% of the total number of articles) were screened by the reviewer, and screening was stopped after 100 abstracts in a row were marked as irrelevant, which is considered a conservative heuristic for when to stop screening using this method (21). In total, 91 articles were accepted using ASRreview, and the reviewer rejected 444 articles. Results from both the authors that screened manually and from screening via ASreview were then manually merged into the final list of selected articles, with any final duplicates removed (Figure 1). Where there was a divergence between the manually reviewed and the software-reviewed articles, one author came in as a third reviewer to decide to reject or accept the article for the next round.

At the end of the abstract screening stage, and after aligning with ASReview results, 327 articles out of 5,129 were moved to full-text analysis, the majority of which were related to *Salmonella* spp. (195 titles), then *E. coli* (111 titles), and finally HEV (21) (Figure 1). Eighty-two articles (25%) were found to be entirely relevant to our research question after full-text analysis. They were selected for data extraction and the risk of bias analysis.

### 2.2 Extracted data

The following data were extracted from the articles by two reviewers independently to ensure harmonisation: effect measures (e.g., odds ratios or risk ratios), type of BSM, study location, the language used, title and author, year of publication, type of study, and production stage where the BSM was applied. From each article, one or more point estimates of the effect measures and their associated confidence intervals (CI) were extracted and sometimes recalculated from raw data when possible (see next section). Each extracted point estimate with its CI represents an “observation.” A publication may contain several observations, as it may study several BSMs. All observations were subsequently grouped based on the definition (where reported) or description of the BSM they best fell into, which was reported in the present study as a BSM. Therefore, a BSM in this article was defined as the combination of the main category, subcategory, and specific action taken on the farm to prevent pathogen presence (Tables 2, 3) Extracted effect measures from the articles were then cleaned by six authors, resulting in the removal of 50 articles at this stage. The reasons for exclusion at this point were (i) a lack of usable effect measures (e.g., unclear at which stage BSMs were administrated, or the data was inconsistent (in some tables, the numbers did not add up to info from other tables; *n* = 34), (ii) a lack of raw effect measures (*n* = 4), (iii) not relevant to our research question (*n* = 7), (iv) unclearly defined BSMs (*n* = 2); conference publication (*n* = 1), and (v) only two reports with three unique BSM for PEC (*n* = 2). In total, 50 articles were excluded at this stage, leading to a final dataset of 32 articles for analysis (for details on reasons for exclusion, see Preferred Reporting Items for Systematic Reviews and Meta-Analyses [PRISMA] flow diagram in Figure 1).

**Table 2:** Extracted biosecurity measures against *Salmonella* spp. from the literature.

Biosecurity measure	Definition	Measure Type	Reference
AgeGroups|GroupSeparation|MixingPigs	No contacts between animals of different age groups	Internal	(40)
AiAo|AiAo|MixingPigs	All-in-all-out	Both	(EFSA) (33, 35, 40–43)
BootDisinfection|Clothing|Humans	Boot disinfection	Both	(72, 73)
ChangePlaceTwice|Clean|CleanDisinf	Changing place of huts/pens twice a year vs. once or less (outdoor pig farming)	Internal	(74)
CleanDrinkers|CleanFeedWater|CleanDisinf	Cleaning of drinkers	Internal	(35, 72)
CleanFeedSystem|CleanFeedWater|CleanDisinf	Feed pipes / system is cleaned	Internal	(74)
CleanFreqAnimals|Clean|CleanDisinf	Frequency of removal of faecal material from sows (less than 2 times/day vs. more than 2 times a day)	Internal	(44)
Cleaning|Clean|CleanDisinf	Cleaning of pens, sections, barns	Both	(40)
ClosedBarn|ContactAvoidance|OtherAnimals	Completely closed barn / bird-proof houses	External	(35, 75)
ClosedFeedStorage|FeedSafety|FeedWaterBedding	Storage room for feed and equipment	External	(35, 73)
ClosedHerd|SourcePigs|Purchase	Closed herd without introduction of new pigs, vs. open herd	External	(73) (EFSA) (33, 42)
DeliveryRoom|HygieneFacility|Humans	Hygiene lock is present / defined contaminated-clean areas	External	(43)
Disinfection|Disinf|CleanDisinf	Disinfection	Both	(43) (44–46) (35)
DistManureSlatFloor|ManureRemoval|CleanDisinf	Big distance between pit manure and slatted floor	Internal	(35)
EmptyClean3|Downtime|CleanDisinf	>3 Days empty and clean before re-populating	Internal	(72)
EmptyClean6|Downtime|CleanDisinf	>6 Days empty and clean before re-populating	Internal	(72)
EmptyClean7|Downtime|CleanDisinf	>7 Days empty and clean before re-populating	Internal	(72)
EmptyManureBatch|ManureRemoval|CleanDisinf	Manure-free between batches	Internal	(43, 72)
EmptyManurePitFreq|ManureRemoval|CleanDisinf	Emptying of the manure pit > = 0.5 times per month	Internal	(35)
EquipDisinf|SepEquip|MaterialEquipment	Disinfection of equipment	Both	(35) (18, 35)
FarmClothesBoots|Clothing|Humans	Farm specific clothes and/or boots are used	External	(40) (18)
FarmEquip|SepEquip|MaterialEquipment	Not sharing farm equipment with other enterprises vs. doing so	External	(35, 73)
FarmShower|HygieneFacility|Humans	Shower-in entry to farm	External	(38)
FeedAcid|AcidUse|FeedWaterBedding	Organic acid in feed	Tertiary Measure	(30), (EFSA) (33) (34) (29) (31)
FeedWaterAcid|AcidUse|FeedWaterBedding	Acidification of water or feed	Tertiary Measure	(40)
FinisherSep|AiAo|MixingPigs	Strategic movement of animals to cleaned and disinfected finishing units / separate section for finishers	Internal	(40)
HygieneLock|HygieneFacility|Humans	Hygiene lock is present / defined contaminated-clean areas	External	(40) (74) (43)
Less2Suppliers|SourcePigs|Purchase	Number of suppliers 0 or 1 vs. 2 or more	External	(40) (76)
Less4Suppliers|SourcePigs|Purchase	Number of suppliers 0 to 3 vs. 3 or more	External	(43)
LessPigsInPen12|PigDensity|MixingPigs	<12 pigs per pen	Internal	(35)
LessPigsInPen20|PigDensity|MixingPigs	<20 pigs per pen	Internal	(37)
ManureRemovalFreq|ManureRemoval|CleanDisinf	Emptying of manure in crate > = 2 times a day	Internal	(72) (72, 77)
MunicipalWater|WaterOrigin|FeedWaterBedding	Municipal or filtered water / deep well vs. Surface/ unfiltered water / superficial well	External	(35) (73, 76) (39)
NoContactBetweenPens|GroupSeparation|MixingPigs	No snout contact between pens	Internal	(43) (78)
NoDrivingThroughSections|GroupSeparation|MixingPigs	Pigs are not driven through occupied barn sections	Internal	(40) (43)
NoMinglingFatteners|GroupSeparation|MixingPigs	No mingling of fatteners from different pens	Internal	(18)
NoSickBayReenter|GroupSeparation|MixingPigs	Not mixing sick pigs with mainstream pigs after recovery vs. doing so	Internal	(73)
NotFeedFloor|FeedSafety|FeedWaterBedding	Not distributing feed on the floor vs. doing so	Internal	(78)
PerimeterFence|ContactAvoidance|OtherAnimals	Fence around the farm	External	(35)
PigsInPen|PigDensity|MixingPigs	Number of pigs per pen	Internal	(EFSA) (33, 36)
PigsInPenCont|PigDensity|MixingPigs	Pig density in pen as continuous outcome per 10 pigs	Both	(78)
Quarantine|Quarantine|Purchase	Quarantine for animal introduction	External	(40, 43) (74)
RodentControl|PestControl|OtherAnimals	Control of rodents	Both	(40) (74) (35) (37) (36) (38) (39)
SepCarcassDisposal|Carcass|OtherAnimals	Disposal of dead pigs outside of the pig barn	Internal	(35)
SepVehicle|SepVehicle|Vehicles	Using separate transporter for each age group	Internal	(18)
SickBay|GroupSeparation|MixingPigs	Using dedicated pens for sickbay vs. not	Internal	(73, 79)
UseBiosecurityPlan|Document|CleanDisinf	Using a written hygiene / biosecurity plan vs. no plan	Both	(73)
VehicleDisinf|VehCleanDisinf|Vehicles	Vehicle disinfection	External	(73) (18, 76)
VehicleFarmEntry|FeedVehicle|Vehicles	Vehicles enter farm perimeter	External	(80)
WaterAcid|AcidUse|FeedWaterBedding	Organic acid in water	Tertiary Measure	(32) (29, 34)

For each measure, a definition, the type of biosecurity (internal, external, both, or tertiary), and the category assigned is provided.

**Table 3:** Extracted biosecurity measures against HEV from the literature.

Biosecurity measure	Definition	Measure type	References
CleanFreq|Clean|CleanDisinf	1 cleaning procedure per day vs. 1 per week or less	Internal	(81)
DistManureSlatFloor|ManureRemoval|CleanDisinf	Big distance between manure pit and slatted floor	Internal	(20)
EmptyDry4|Downtime|CleanDisinf	>4 Days empty and cleaning/drying before re-populating	Internal	(20)
FarmClothesBoots|Clothing|Humans	Farm specific clothes and/or boots are used	External	(20)
LessPigsInPen17|PigDensity|MixingPigs	<17 Pigs per pen	Internal	(20)
MunicipalWater|WaterOrigin|FeedWaterBedding	Municipal or filtered water / deep well vs. surface/ unfiltered water / superficial well	External	(53)
NoMinglingPiglets|GroupSeparation|MixingPigs	No mingling of piglets from different pens / litters / premises	Internal	(20)
PerimeterFence|ContactAvoidance|OtherAnimals	Fence around the farm	External	(53)
Quarantine|Quarantine|Purchase	Quarantine before animal introduction	External	(53) (52)
VehicleDisinf|VehCleanDisinf|Vehicles	Vehicle disinfection	External	(53) (52)

For each measure, a definition, the type of biosecurity (internal and, external) and the category assigned is provided.

### 2.3 Data analysis

The extracted data was analysed using the statistical software R version 4.0.3 (22). For the evidence synthesis of the data analysis, the R package metafor version 3.0-2 (23) was used.

Some studies provided raw data from which the effect measures could be calculated, whereas others reported an effect measure derived from a univariate and/or multivariate regression analysis. Whenever a study provided raw data, this was used to calculate the OR and its variance using the escalc() function from the metafor package. Otherwise, the reported effect size and the corresponding confidence interval (CI) were used.

As the aim was to compare the effectiveness of BSMs in different stages of pig production, we pooled all observations for a given combination of production stage, pathogen, and BSM. Since the considered publications studied pig production in different countries and periods, we expected some heterogeneity between the studies. Additionally, many publications assessed BSM effectiveness at stages different from their implementation stage, further contributing to heterogeneity. We employed a random-effects model to account for expected heterogeneity. We established that a minimum of five observations were needed for performing a meta-analysis, in line with the established methodology, as suggested, for example, refer to references (24, 25). Thus, whenever for a given combination of the production stage, pathogen, and BSM, five or more observations could be found, we performed a meta-analysis using the metafor function rma.uni() based on a random-effects model using restricted maximum likelihood estimator for estimating the amount of statistical heterogeneity. Statistical heterogeneity was assessed using the *I*^2^ statistic, and our interpretation of whether the corresponding I^2^ values may represent “moderate” or “substantial” heterogeneity followed (26). There were only two combinations of production stage, pathogen, and BSM, where five or more observations were available: acidification of feed and acidification of drinking water for *Salmonella* during the fattening stage. To extract more insights, we aggregated publications on the same BSM regardless of production stage, performing a meta-analysis for combinations with at least five observations. For five combinations of pathogen and BSM, there were five observations: acidification of feed, acidification of drinking water, all-in and all-out production, disinfection, and rodent control.

Therefore, evidence synthesis was carried out in two different ways. The first approach described above considered a combination of BSM, pathogen and production stage. This approach is referred to as “considering the production stage.” The second approach considered combinations of BSM and pathogen, irrespective of the production stage. This approach is referred to as “ignoring the production stage.”

To provide a formal assessment of publication bias concerning the observations included in the meta-analyses, we created contour-enhanced funnel plots using the function funnel() from the metafor package. We performed Egger’s test using the regtest() function, also from the metafor package (which performed a weighted regression with multiplicative dispersion).

Finally, we did a sensitivity analysis for the meta-analyses using the influence() function from the metafor package.

We only provided bias analyses and sensitivity analyses for the meta-analyses, which showed statistically significant summary OR estimates.

### 2.4 Risk of bias analysis

All studies included in the data extraction stage were evaluated for risk of bias (RoB) by using protocols adapted from the study mentioned in Kim et al. (27) for observational studies and from the study in Higgins et al. (28) for experimental studies. Both protocols provided a framework for assessing the likelihood or risk that bias concerning the study’s outcome could have been introduced. All RoB analyses were conducted in duplicate independently by two reviewers. In total, either seven or eight domains of bias, depending on the study type, were assessed: selection bias, allocation concealment (for experimental studies only), confounding variables, performance bias, detection bias, attrition bias, reporting bias, and an “other bias” domain including questions on funding sources and potential conflicts of interest. To assess the RoB in the context of BSMs and the respective article, specific signalling questions for each domain were formulated to guide the authors in classifying RoB as either low, medium, or high. The masks applied during the RoB assessment, including the signalling questions, are presented in Supplementary Tables S2 and S3. The overall assessment of each article was based on the combined assessments of all RoB domains. In cases where the majority of the signalling questions were at a medium level, or one was rated as a high level, the domain was classified as having a high RoB. If one of the domains was rated as having a high RoB, the overall assessment was also rated as having a high RoB.

## 3 Results

This analysis included 157 observations of 54 BSMs extracted from 32 articles. Amongst the 32 studies reviewed, eight main types of BSMs were identified, which were as follows: (a) humans, that is, interventions that involved humans, such as hand washing, showering, etc.; (b) mixing of pigs; (c) cleaning and disinfection; (d) vehicles; (e) feed, water, and bedding; (f) purchase of animals; (g) equipment, and (h) other animals. The number of observations for each type of BSM by the two pathogens studied in this review can be found in Supplementary Figure S2.

### 3.1 Study characteristics

### 3.2 *Salmonella* spp.

#### 3.2.1 Number of BSMs associated with *Salmonella* spp. in this study

Overall, 2,003 articles about the BSMs against *Salmonella* spp. were identified in the literature, with 28 of those (1.4%) eligible for final analysis (Figure 1). These articles were published between 1997 and 2019 (median: 2009, interquartile range [IQR] = 15). In total, 50 BSMs were identified for *Salmonella* spp. (Table 2).

#### 3.2.2 Internal and external biosecurity measures for *Salmonella* spp.

Overall, the majority of the extracted BSMs belonged to internal biosecurity (*n* = 24, Table 2, note that the number 24 refers to BSMs which are classified as strictly “internal” in Table 2), that is, addressing measures that would prevent the spread of a pathogen within the farm. However, BSMs that showed a high protective effect were mostly those covering external biosecurity, aimed at preventing transmission of pathogens between farms and represented 15 of the extracted BSMs for *Salmonella* spp. in Table 2. Of note, eight BSMs were attributed to both internal and external biosecurity measures for this pathogen and a small proportion of three BSMs were identified as relating to tertiary biosecurity, which is usually described as measures aiming to control the ability of animals to deal with the pathogen.

#### 3.2.3 Meta-analysis for *Salmonella* spp.

- Considering the production stage

**Figure 2:**
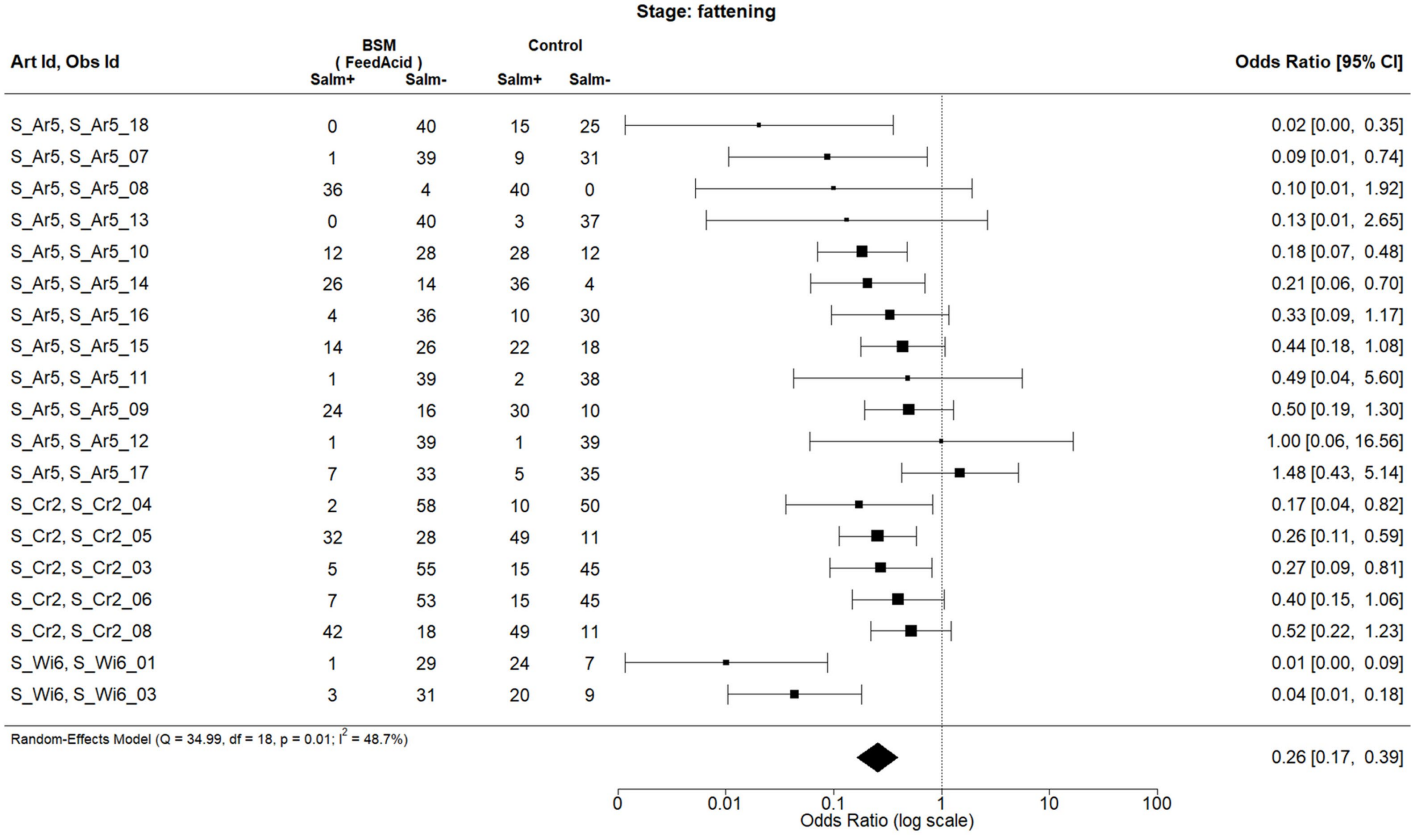
Forest plot of random-effects meta-analysis (with restricted maximum likelihood estimator of the amount of heterogeneity) for the biosecurity measure (BSM) acidification of feed for *Salmonella* spp. at the fattening stage. Each observation is encoded with internal BIOPIGEE designations for the article ID (Art Id) and the observation ID (Obs Id). Where available, the number of *Salmonella* spp. positive (‘Salm+’) and negative (‘Salm−’) samples for the BSM and the control condition, as stated in the corresponding publication, are provided. Each square indicates the odds ratio (OR) for one observation, with the size of the square indicating the weight with which that observation contributed to the summary value of the meta-analysis. The whiskers on the squares indicate the 95% confidence intervals (CIs) for each observation. The diamond at the bottom showed the summary value of the meta-analysis, indicating a summary OR of 0.26 with a 95% CI of 0.17–0.39.

**Figure 3:**
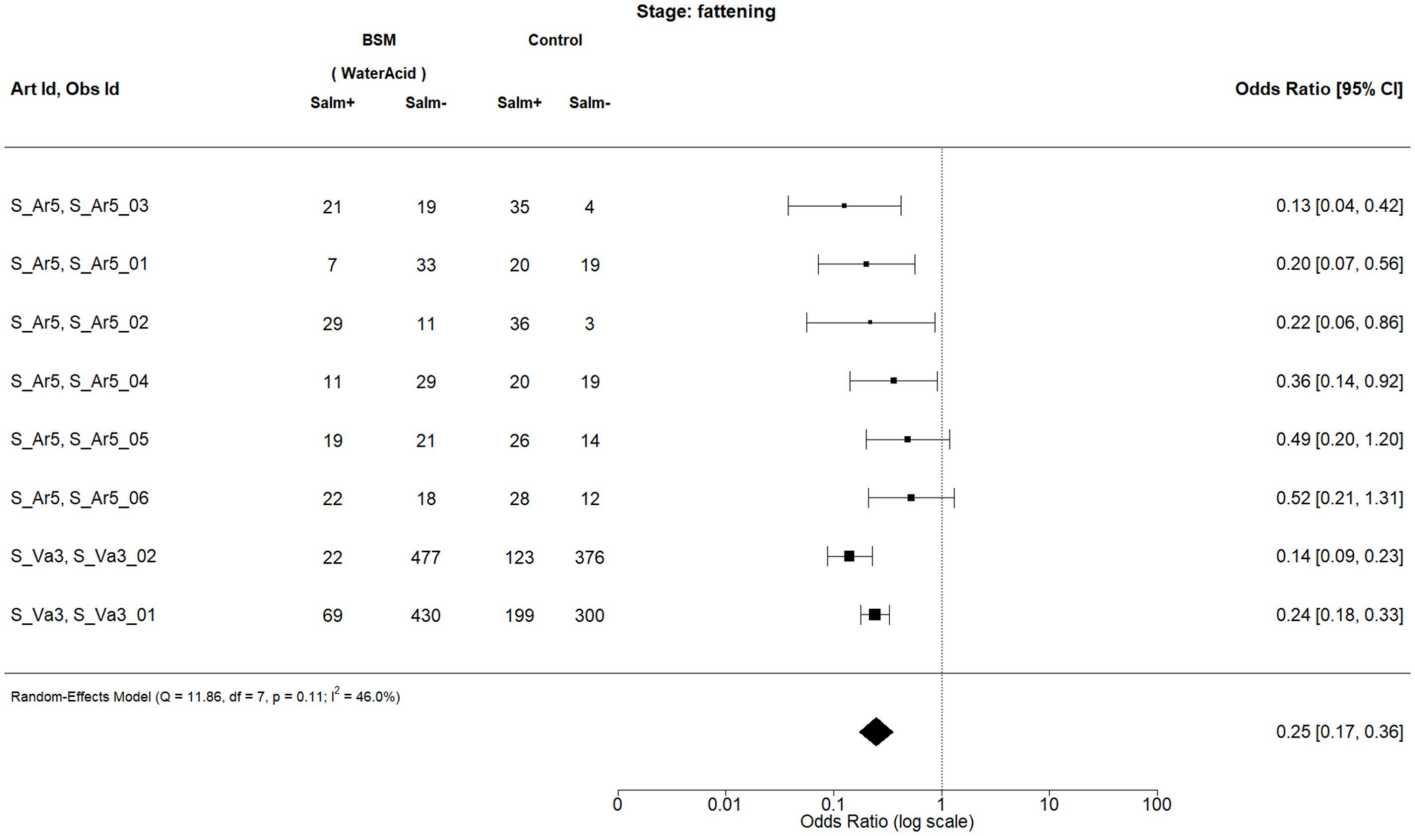
Forest plot of random-effects meta-analysis (with restricted maximum likelihood estimator of the amount of heterogeneity) for the biosecurity measure (BSM) water acidification for *Salmonella* spp. at the fattening stage. Each observation is encoded with internal BIOPIGEE designations for the article ID (Art Id) and the observation ID (Obs Id). Where available, the number of *Salmonella* spp. positive (‘Salm+’) and negative (‘Salm−’) samples for the BSM and the control condition, as stated in the corresponding publication, are provided. Each square indicates the odds ratio (OR) for one observation, with the size of the square indicating the weight with which that observation contributed to the summary value of the meta-analysis. The whiskers on the squares indicate the 95% confidence intervals (CIs) for each observation. The diamond at the bottom indicated the summary value of the meta-analysis, indicating a summary OR of 0.25 with a 95% CI of 0.17–0.36.

When considering the production stage, two BSMs met the inclusion criteria for meta-analysis, being the acidification of feed (FeedAcid|AcidUse|FeedWaterBedding; Figure 2) and the acidification of water (WaterAcid|AcidUse|FeedWaterBedding; Figure 3). The observations and the corresponding articles are designated with unique identifiers throughout the forest plots presented in this analysis. A list of the identifiers and information on the corresponding publication (title, author, country of study, etc.) can be found in Supplementary Table S6.

For feed acidification, there were 19 observations from three publications, that is, S_Ar5, which corresponds to (29), S_Cr2 (30) and S_Wi6 (31) (Figure 2). All three publications dealt with pigs in Spain and were published between 2007 and 2013. The summary estimate for the OR due to feed acidification is 0.26, 95% CI: 0.17–0.39. The heterogeneity statistic *I*^2^ has a point estimate of 48.7%, indicating a moderate heterogeneity between the observations.

For water acidification, there were eight observations from two publications: S_Ar5 and S_Va3 (32) (Figure 3). While the first publication was from Spain in 2013, the latter was from The Netherlands in 2001. The summary estimate for the OR due to water acidification is 0.25, 95% CI: 0.17–0.36. The *I*^2^ is 46.0%, indicating a moderate heterogeneity between the observations.

- Ignoring the production stage

**Figure 4:**
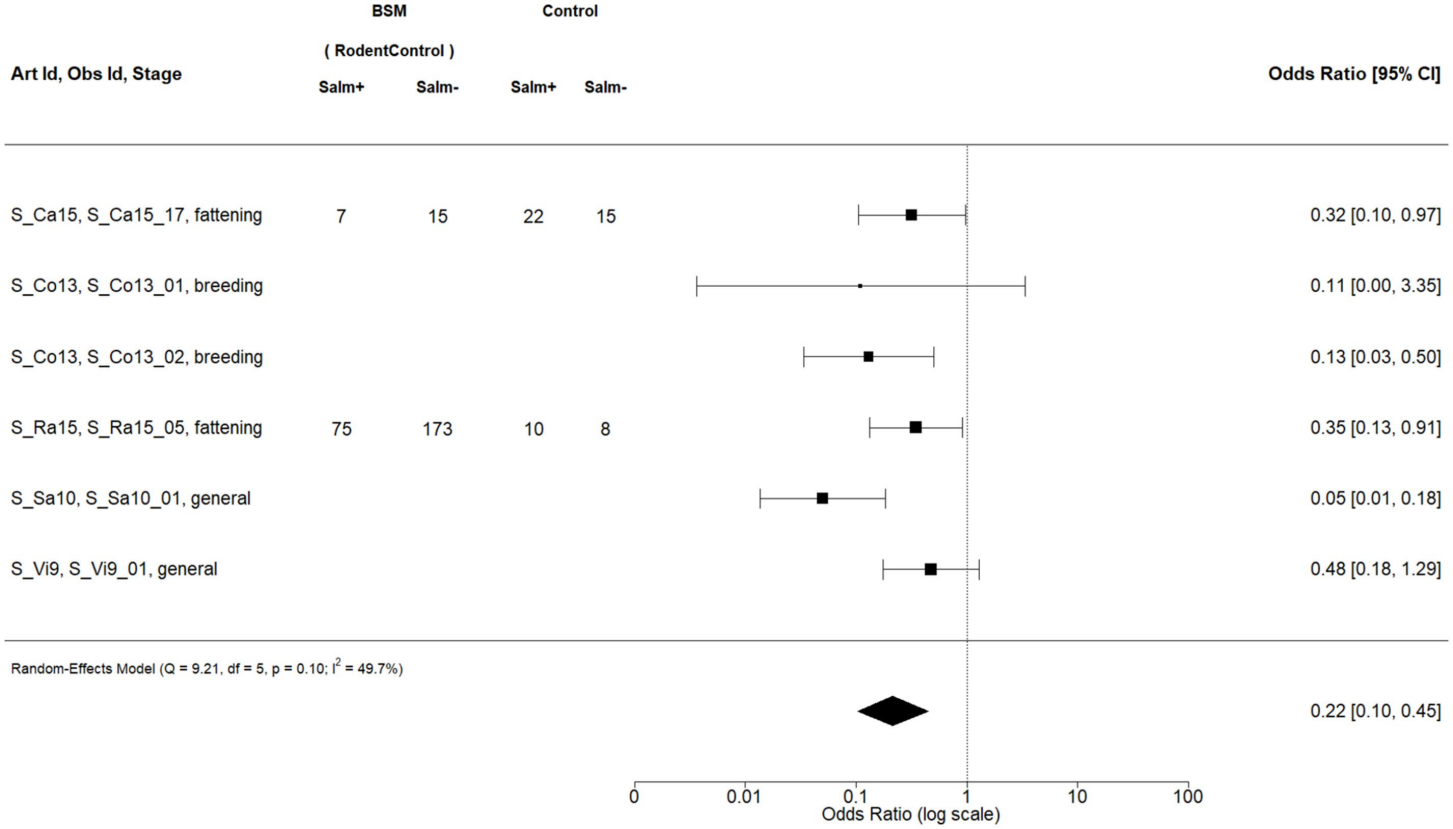
Forest plot of random-effects meta-analysis (with restricted maximum likelihood estimator of the amount of heterogeneity) for the biosecurity measure (BSM) rodent control for *Salmonella* spp. ignoring the production stage. Each observation is encoded with internal BIOPIGEE designations for the article ID (Art Id), the observation ID (Obs Id), and the production stage. Where available, the number of *Salmonella* spp. positive (‘Salm+’) and negative (‘Salm−’) samples for the BSM and the control condition, as stated in the corresponding publication, are provided. Each square indicates the odds ratio (OR) for one observation, with the size of the square indicating the weight with which that observation contributed to the summary value of the meta-analysis. The whiskers on the squares indicate the 95% confidence intervals (CI) for each observation. The diamond at the bottom indicated the summary value of the meta-analysis, indicating a summary OR of 0.22 with a 95% CI of 0.10–0.45.

**Figure 5:**
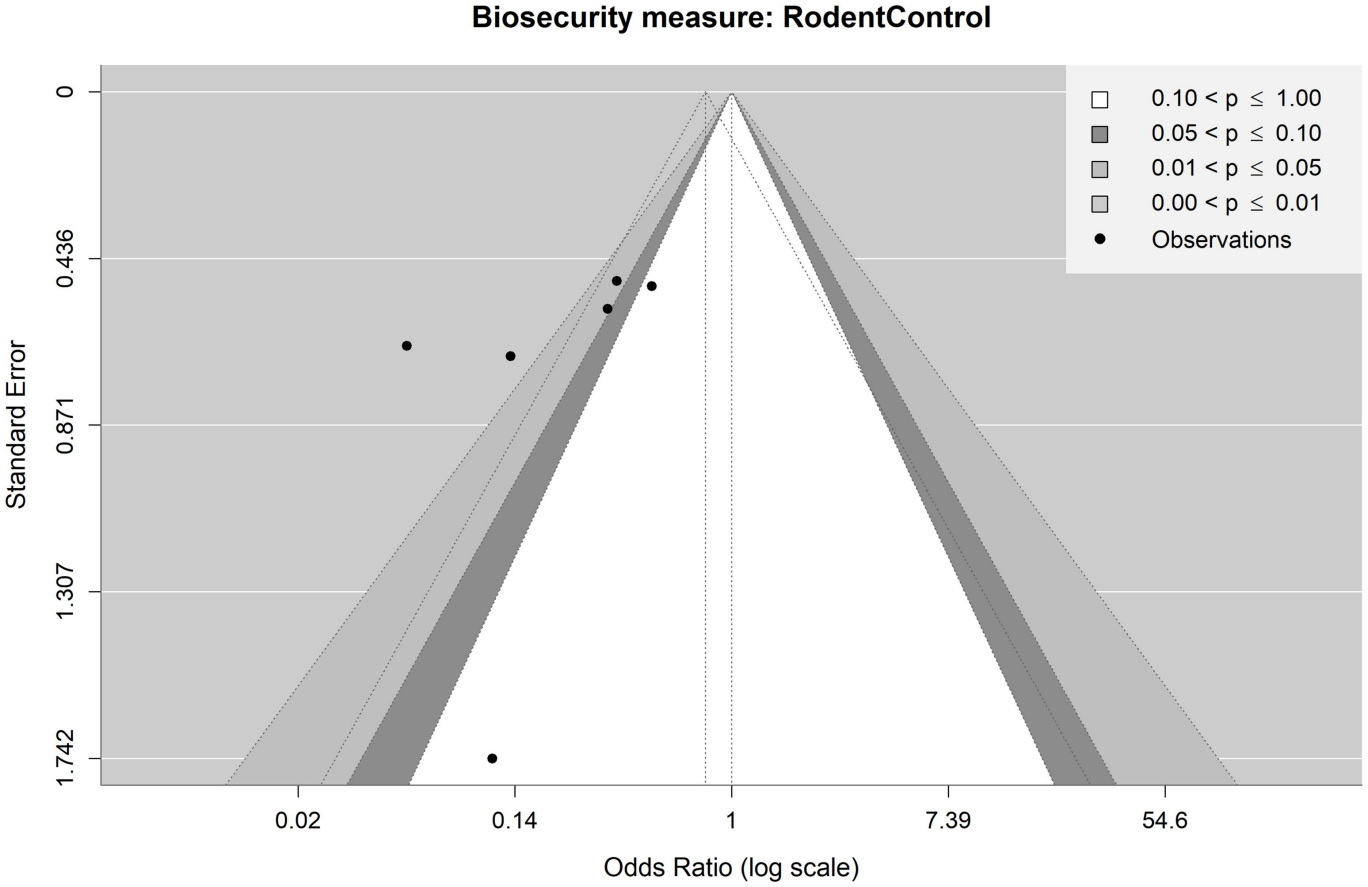
Contour-enhanced funnel plot for observations included in the meta-analysis on the mitigating effect of rodent control on *Salmonella* spp. infection in pig production, ignoring the stage. Each point is an observation from the literature. The shaded areas help identify whether observations lie above or under statistical significance. The funnel with dotted lines indicates how the observations are scattered around the summary OR of 0.22.

**Figure 6:**
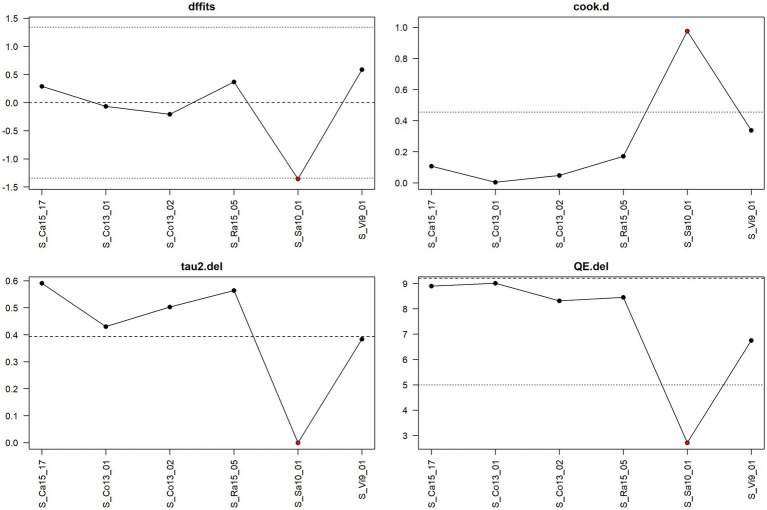
Diagnostic plot for sensitivity analysis for the meta-analysis on the mitigating effect of rodent control on *Salmonella* spp. infection ignoring the stage in pig production.

When pooling all observations for a BSM, regardless of the production stage, five BSMs had sufficient data for meta-analysis: acidification of feed and water, all-in and all-out production (AiAo|AiAo|MixingPigs), disinfection (Disinfection|Disinf|CleanDisinf), and rodent control (RodentControl|PestControl|OtherAnimals). Acidification of feed and water, along with rodent control, demonstrated statistically significant protective effects. Note that in this section, we only show the forest plot (Figure 4), the funnel plot (Figure 5), and the sensitivity analysis (Figure 6) for rodent control. The forest plots for the remaining four BSMs can be found in the Supplementary material (Supplementary Figure S3). As noted above, funnel plots (Supplementary Figure S4) and sensitivity analysis (Supplementary Figure S5) were only provided for the BSMs which demonstrated statistically significant protective effects, in this case of ignoring the production stage which means acidification of feed and acidification of water.

For feed acidification, 21 observations from five publications were finally included. That is, in addition to the aforementioned observation for the fattening stage from S_Ar5, S_Cr2 and S_Wi6, there were now observations from S_Ef1, which is (33) and considers acidification generally throughout the EU and from S_Ru5 which is (34) and considers feed acidification at the weaning stage in Italy. The summary estimate for the OR was 0.27, 95% CI 0.18–0.41. The heterogeneity statistic I^2^ was 62.2%, indicating a substantial heterogeneity between the observations.

One more observation was included for water acidification compared to the analysis considering the production stage, which came from S_Ru5 considering water acidification at the weaning stage in Italy, published in 2018. The summary estimate for the OR is 0.23, 95% CI: 0.16–0.34. The heterogeneity statistic *I*^2^ was 44.1%, indicating a moderate heterogeneity between the observations.

For rodent control, there were six observations from five publications (Figure 4): S_Ca15 (35) considers fattening pigs on Reunion Island, an overseas department of France in the Indian Ocean; S_Co13 (36) considers breeding pigs in Portugal, S_Ra15 (37) considers finishing pigs in Canada, whereas S_Sa10 (38) and S_Vi9 (39) consider pigs in Spain. The summary estimate for the OR was 0.22, 95% CI: 0.10–0.45. The heterogeneity statistic *I*^2^ was 49.7%, indicating a moderate statistical heterogeneity between the observations.

For all-in and all-out production, there were six observations from six publications (see Supplementary Figure S3 A1): S_Al19 (40) for pigs in Germany in 2000, S_Ca15 (35) for pigs on Reunion Island in 2010, S_Da15 (41) for pigs in the US in 1997, S_Do12 (42) for pigs in Poland in 2015, S_Ef1 (33) for pigs in the EU in 2011, and S_St6 (43) for pigs in Denmark in 2001. The summary estimate for the OR was 0.79 (95% CI: 0.52–1.18). The heterogeneity statistic (*I*^2^) was 19.6%, suggesting low statistical heterogeneity. However, some heterogeneity exists, given the varied geographical locations and study periods. One observation (S_Da15_01) had a wide confidence interval due to a zero value in its 2 × 2 table. Removing this observation increased *I*^2^ to 29.3%, indicating borderline to moderate heterogeneity. With a small number of observations and a *p*-value of 0.39 for the heterogeneity test, the precision of the heterogeneity estimate was low, suggesting the true *I*^2^ might be even higher than 29.3%.

For disinfection, there were eight observations from six publications (see Supplementary Figure S3B): S_Al19 (40) for pigs in Germany in 2000, S_Ca15 (35) on Reunion Island in 2010, S_Fa8 (44) in France in 2003, S_Ma12 (45) in the UK in 2017, S_St6 (43) for pigs in Denmark in 2001, and S_Va8 (46) in The Netherlands in 2001. The summary estimate for OR was 1.03, 95% CI: 0.46–2.28. The heterogeneity statistic *I*^2^ of 94.3% indicated considerable heterogeneity.

- Bias analysis

**Figure 7:**
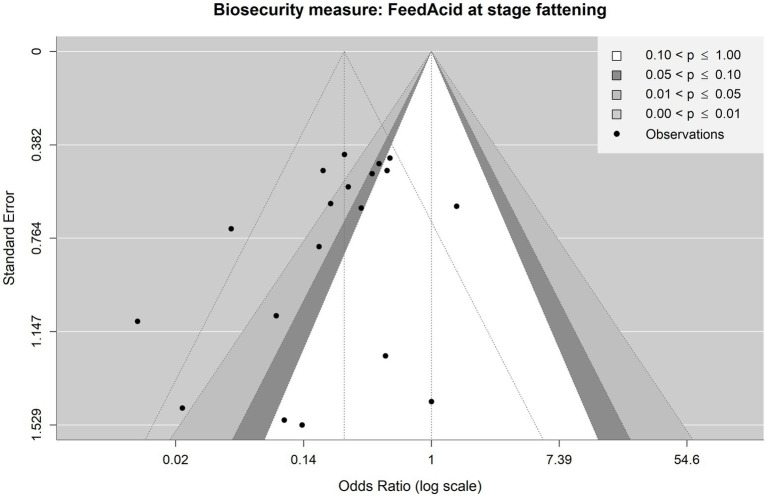
Contour-enhanced funnel plot for observations included in the meta-analysis on the mitigating effect of feed acidification on *Salmonella* spp. Infection at the fattening stage in pig production. Each point is an observation from the literature. The shaded areas help identify whether observations lie above or under statistical significance. The funnel with dotted lines indicates how the observations are scattered around the summary OR of 0.26.

First, we consider the meta-analyses, which considered the production stage. Figure 7 shows the funnel plot for the observations concerning feed acidification at the fattening stage. Visual inspection of the funnel with the dotted lines (i.e., the funnel plot centred at the summary OR of 0.26) suggests a possible small-study bias: there are three observations at the bottom (i.e., observations with high standard errors and, therefore, smaller sample sizes) left-hand side from the vertical line (i.e., showing an effectivity against *Salmonella* spp. with lower than summary OR, meaning a relatively high effectivity relative to the summary OR). However, there is only one corresponding small study observation at the bottom right-hand side with higher than summary OR. This visually suggestive asymmetry between more publications of small studies reporting high effectivity and fewer with low effectivity is in contrast to the result of Egger’s test, which does not provide evidence of asymmetry in the funnel plot and hence possible publications bias (*p* = 0.0623) at the 0.05 significance level. Still, as the *p*-value is close to the cut-off of 0.05, it may be interpreted as a tendency that there might be some bias present.

However, taking the statistical significance of the individual observations into account (the contoured plot with the shaded funnel centred at OR = 1), no particular asymmetry in terms of statistical significance is evident upon visual inspection: 9 observations are lying in the areas of statistical significance while 10 lies in the area of no statistical significance. From this perspective, there appears to be no publication bias based on statistical significance.

**Figure 8:**
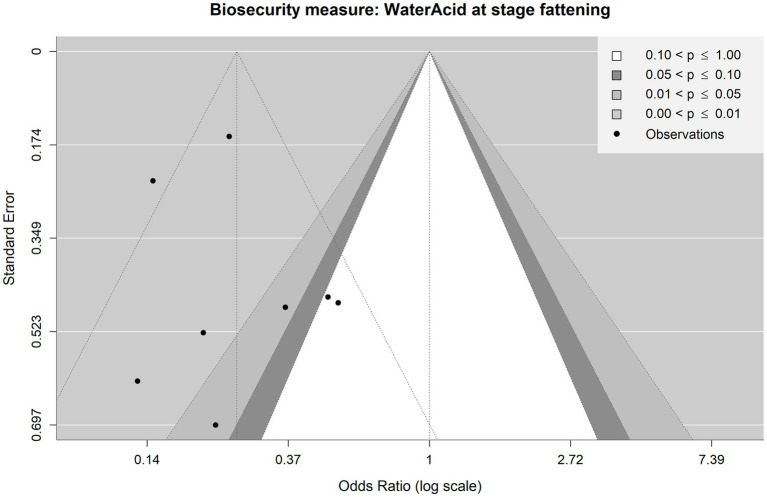
Contour-enhanced funnel plot for observations included in the meta-analysis on the mitigating effect of water acidification on *Salmonella* spp. infection at the fattening stage in pig production. Each point is an observation from the literature. The shaded areas help identify whether observations lie above or under statistical significance. The funnel with dotted lines indicates how the observations are scattered around the summary OR of 0.25.

Figure 8 shows the funnel plot for water acidification at the fattening stage. Visual inspection of the dotted funnel plot suggests a possible small-study bias, with the two observations on the bottom left-hand side having no counterparts on the right-hand side. However, Egger’s test again provided no evidence for funnel plot asymmetry and, hence, publication bias (*p* = 0.5779). It should be noted that in this case, there are less than 10 observations in the meta-analysis. This situation is where Egger’s test can only detect a substantial small-study bias (47). Visual inspection of the contoured funnel plot suggests an asymmetry concerning the significance of the observations (six observations show statistical significance, whereas two observations do not show it), which suggests that there might be a publication bias due to underreported non-significant observations.

Then, we considered the meta-analyses which ignored the production stage. Figure 5 shows the funnel plot for the meta-analysis on rodent control when ignoring the production stage. Visual inspection shows an apparent asymmetry. There are only publications left to the vertical lines of the dotted funnel. A small-study bias is suggested by a single observation with a relatively high standard error left from the perpendicular line with no corresponding observations on the right. However, Egger’s test did not provide evidence for asymmetry (*p* = 0.3320). As noted above, Egger’s test for less than 10 observations is somewhat limited in its informative value. In terms of sensitivity, two observations were non-significant, whereas four observations were significant. Given the small number of observations, there is no apparent bias in terms of significance.

Visual inspection of the funnel plot for acidification of feed ignoring the production stage (Supplementary Figure S4A) indicates a considerable small-study bias, corroborated by Egger’s test (*p* < 0.0001). The contoured funnel plot detects no large asymmetry in terms of statistically significant (11 observations) vs. non-significant (11 observation) data points.

The funnel plot for water acidification ignoring the production stage (Supplementary Figure S4B) shows only one observation in the “small-study” area on the bottom left-hand side and none on the bottom right-hand side. However, these low numbers in observations do not allow us to draw any conclusions. In line with this result Egger’s test did not provide evidence for funnel asymmetry and hence publication bias (*p* = 0.9508).

A large bias with respect to the statistical significance of the individual observations is present as only two observations show non-significant results, while seven observations show significant results, hinting at a possible publication bias for non-significant observations.

- Sensitivity analysis

First, we looked at the meta-analyses which considered the production stage.

**Figure 9:**
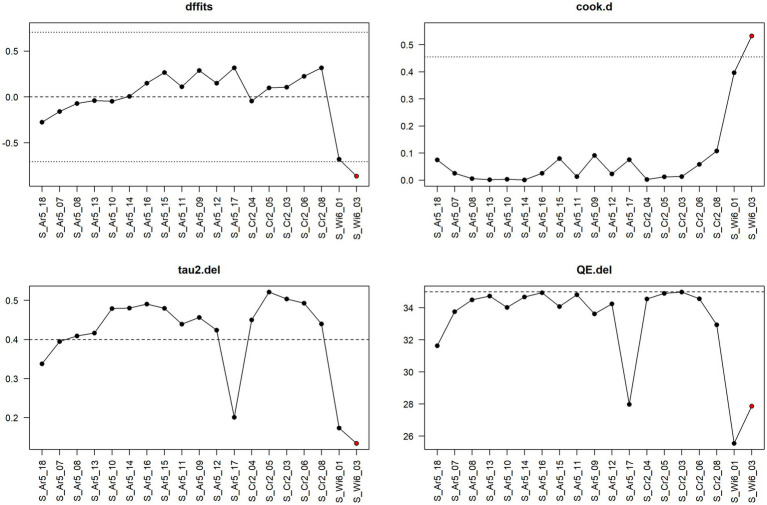
Diagnostic plot for sensitivity analysis for the meta-analysis on the mitigating effect of feed acidification on *Salmonella* spp. infection at the fattening stage in pig production.

Figure 9 shows the result of sensitivity analysis for feed acidification at the fattening stage based on a leave-one-out approach. That is, one observation at a time is left out, and the meta-analysis is re-run to see how the results change. In particular, the values of the following four measures are considered under leaving out one observation: dffits (the studentised difference in fits), cook.d (Cook’s distances), tau2.del (estimate of τ^2^—a measure of between-study variance—the variance of the effect when leaving one particular study out), and QE.del (test statistic for (residual) heterogeneity when leaving one particular study out). The author of the metafor package chose cut-off values for dffits and cook.d values to select whether a corresponding observation might be considered an “influential” one, that is, an observation to which the meta-analytical result is, to a certain extent, sensitive. The cut-off for dffits is defined by values which are larger than $3\cdot \sqrt{p/k-p}$ , where p is the number of model coefficients (in our case with the random effects model, we always have p = 1), and k is the number of observations. In the case of Figure 9 and feed acidification at the fattening stage, k = 19; hence, the cut-offs are about ± 0.71. The cut-off for cook.d is reached if the lower tail area of a chi-square distribution with p degrees of freedom cut-off by the Cook’s distance is larger than 50%.

The cut-offs for dffits and cook.d are indicated in the sensitivity analysis plots as dotted lines. If leaving out one observation leads to meta-analytical results which exceed the cut-offs for dffits and/or cook.d, the corresponding point in the sensitivity plot is indicated in red. Hence, red points indicate that the corresponding observations could be interpreted as being considerably influential.

Thus, Figure 9 shows that the observation S_Wi6_03 is the only one that has a considerable influence, and one also sees that leaving it out decreases considerably the heterogeneity as measured by tau2.del as well as QE.del.

**Figure 10:**
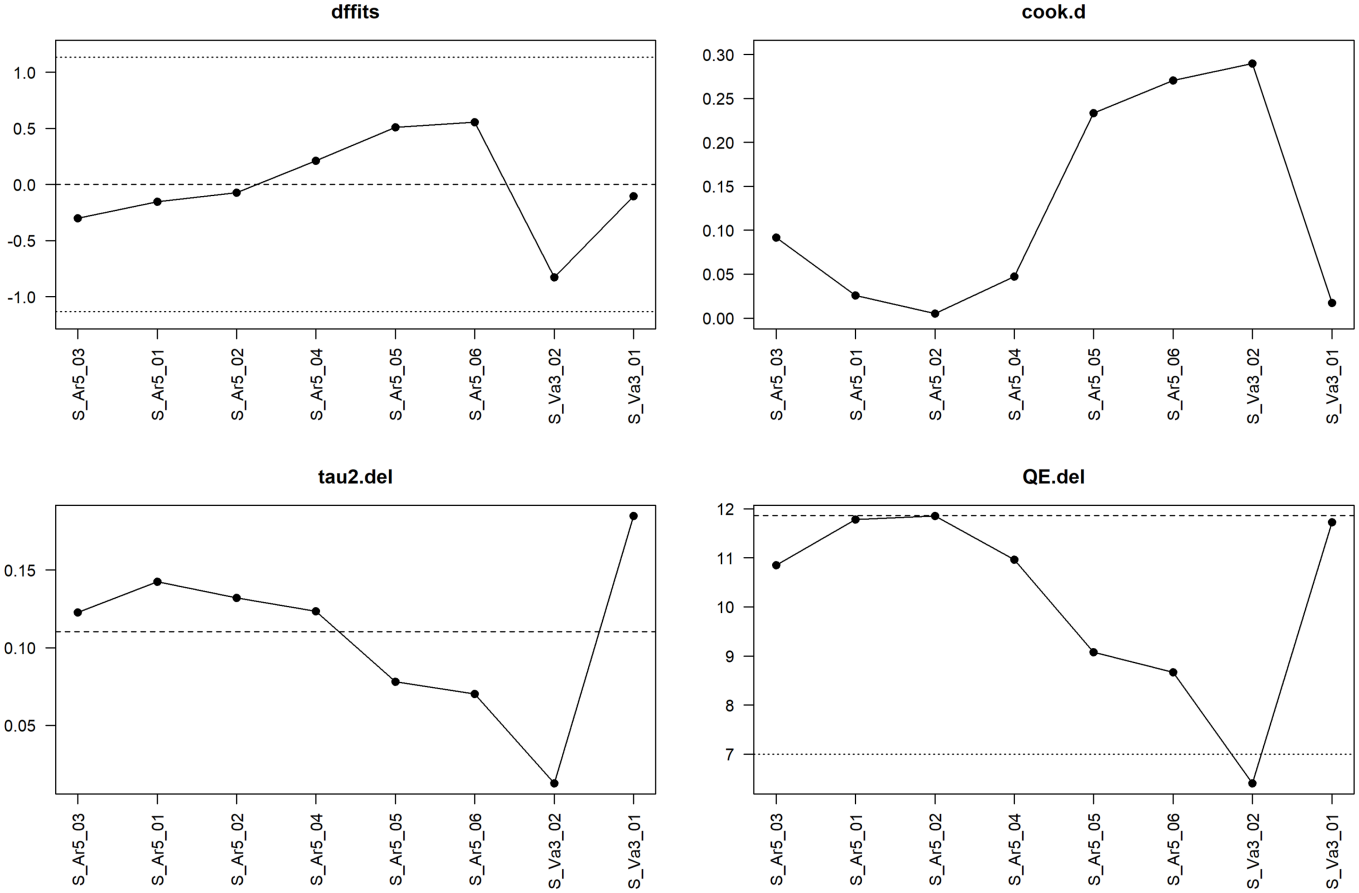
Diagnostic plot for sensitivity analysis for the meta-analysis on the mitigating effect of water acidification on *Salmonella* spp. infection at the fattening stage in pig production.

Figure 10 shows the sensitivity analysis for water acidification at the fattening stage. Here no single observation is considered influential according to the considered measures. Note that the dotted line in the panel for the measure QE.del is not a cut-off for potentially influential observations but one of two horizontal reference lines drawn at the test statistic based on the case that all observations are considered (i.e., without leaving one observation out) and at k–p, i.e., the degrees of freedom of the test statistic. In this case, the test statistic in question is the Q-statistic, which is also shown in Figure 3 and has the value 11.86 (dashed line in Figure 10), and the degrees of freedom are 7 (dotted line in Figure 10).

Then, we looked at the meta-analyses which ignored the production stage.

The sensitivity plot in Figure 6 indicated that the observation S_Sa10_01 considerably exceeds the cook.d cut-off, and leaving it out considerably lowers the heterogeneity of the meta-analysis.

### 3.3 Hepatitis E virus

The database search retrieved 311 hits for HEV, of which 21 articles were screened by full text. Finally, data was extracted from four articles published between 2014 and 2021 (median = 2017.5, IQR = 4.75), from which 10 protective BSMs were found (Table 3). Two BSMs, quarantining pigs and disinfecting vehicles, were reported in two articles, while the others were reported in only one article. All BSMs reduced the odds of an observational unit being HEV-positive. They were attributed 50/50 to external and internal BSMs. There was insufficient information to run a meta-analysis on the BSMs for HEV.

### 3.4 Risk of bias analysis

**Figure 11:**
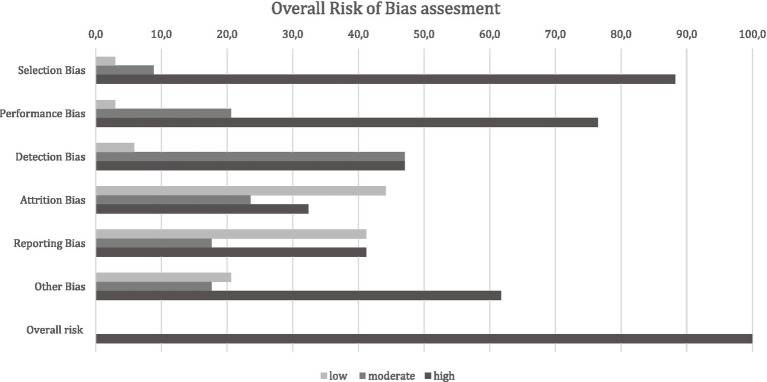
Risk of bias ratings per bias domain for all studies in the final selection stage (*n* = 32). The numbers shown are percentages of articles for each rating by domain.

The outcome of the RoB analysis classified all studies in the final selection stage (observational studies *n* = 27, experimental *n* = 5) to be at a high overall RoB (Figure 11). The high overall rating of bias risk was mainly driven by the high scores in the selection bias (87.5%), performance bias- (75%), and “other bias” (59.4%) domains (Supplementary Table S4). The high rating in the selection bias domain, especially in observational (i.e., mainly cross-sectional) study designs, was based on the lack of or unclear random selection of animals or animal groups and even more so by missing consideration of potential confounders in the respective studies. In the context of performance bias, the collection of samples in the respective studies was either unclear, not described, or performed non-blinded. The main signalling question in ‘other bias’ concerning conflict of interest led to the high rating in this domain. The detailed ratings of each bias domain for all studies in the final selection stage are presented in Supplementary Table S5.

## 4 Discussion

### 4.1 Identified BSMs in the context of specific pathogen control

#### 4.1.1 Identified BSMs in the context of *Salmonella* control

Only two BSMs had sufficient data to perform a meta-analysis with respect to *Salmonella* spp. control and considering the production stage: acidification of water and feed. The meta-analysis revealed that farms that did not apply acidification of feed or water had about 4 times the odds of being *Salmonella* spp. favourable than farms which used acidification. This outcome was primarily based on observations made during the fattening stage and was, therefore, only “evidence-based” for this production stage. However, if one considers that some evidence from feed acidification at other stages of production found in the literature points in the same direction (33, 34), and if one considers the general hypothesis that acidification of the intestinal tract should prevent the colonisation of *Salmonella* spp. in the gastrointestinal tract of pigs (32), then acidification could be an effective BSM against *Salmonella* spp. at all stages throughout the pig production line. However, the studies in this analysis often lacked details on acid concentrations and application methods (to water or feed) and typically measured pathogen burden by bacteria count in pig tissues. As such, further research on the effects of organic acids on *Salmonella* spp. in feed and water would strengthen the evidence supporting their use as an effective BSM for controlling *Salmonella* spp. in pigs.

When ignoring the production stage, the meta-analysis showed one additional BSM to be effective against *Salmonella* spp., namely rodent control. The analysis showed that the reduction of the odds for *Salmonella* spp. in pigs for this BSM is similar to the one found for acidification. Rodents can acquire and carry *Salmonella* spp. infections for several months. They are considered highly efficient vectors and amplifiers of *Salmonella* spp. on farms. Due to their high carriage rates and rapid reproduction (48, 49). Mouse droppings can contain up to 10^4^ CFU/g of *Salmonella* spp., and a single mouse can shed 100 faecal pellets per day (50). The importance of rodent control, specifically of rodent bait, on pig farms in limiting *Salmonella* spp. was recently confirmed in a cross-sectional study performed within the biosecurity practices for pig farming across Europe (BIOPIGEE) project (51).

#### 4.1.2 Identified BSMs in the context of HEV control

There was not enough data in the included publications to perform a meta-analysis on BSMs for HEV. Therefore, the literature on this pathogen is discussed here qualitatively. The included publications report several external BSMs to be effective in preventing HEV from entering farms. The external BSM quarantining pigs may prevent direct and indirect transmission between pigs of different origins (52, 53). A European study of biosecurity measures to control HEV that was published after the current reviewing process was finalised, also found a quarantine area as a key biosecurity measure related to a lower HEV risk (54). Quarantine is a common practice before the introduction of gilts to the herd (55), and gilts are often purchased at an age category in which HEV is most prevalent (fattening period (6),). Therefore, quarantining may prevent transmission to other gilts and sows on the farm.

Indirect transmission of HEV between farms may be facilitated by vehicles, rodents, or people that enter multiple farms (15). Studies have found a significant reduction in the odds of a positive HEV farm when disinfecting vehicles, having a fence around the farm (53), and wearing farm-specific clothes or boots (20), which may all prevent the indirect transmission of HEV. Municipal water is more protective for HEVs than private wells. Possibly, private wells can be HEV contaminated, for instance, via run-off of water or manure (56). HEV is known to be prevalent (and probably survive) in water (57), and pig infection occurs orally (58). Still, more research is needed on how wells can become contaminated with HEV and whether water from the nozzle inside the pig barns is HEV contaminated.

Five BSMs are related to internal farm biosecurity, with the associated main categories being cleaning and disinfection, and avoiding mixing of pigs. Distances exceeding 0.8 meters between the manure pit and slatted floor, extended downtime periods, and frequent cleaning significantly increase the likelihood of farms testing negative for HEV. These measures align with strategies aimed at interrupting HEV transmission within farms by minimizing direct contact and faecal exposure. The effectiveness of implementing specific biosecurity measures to control HEV in pig farms may vary based on infection status, age group affected, farm type, and other farm-specific epidemiological factors (15). For instance, on farms with sporadic HEV outbreaks of the implementation of external measures may prevent new outbreaks from coming from outside. However, internal biosecurity measures may be more effective because the within-farm and farm-level prevalence of HEV are both high in majority of the European countries (6).

#### 4.1.3 Risk of bias

The result of an overall high RoB in all studies in the final article selection requires caution regarding the use and interpretation of the results presented in our study. Here, it must be noted that the applied approach for assessing RoB was strict and comprehensive and that this outcome does not necessarily invalidate the results and conclusions of these studies. However, the result does highlight recurring issues that need to be addressed to aid future research in the context of BSM application and their effectiveness. Particularly in the selection of farm operations or animal groups, the consideration of potential confounders in connection with the outcome of the efficacy of BSMs, the sampling design, and the reporting of negative results ought to receive closer attention. Two other factors that contributed strongly to the rating of a high bias were the randomisation process in the selection bias domain and blinding in the detection and performance bias domain, especially in observational studies. Another aspect was that a statement about conflicts of interest was often missing in primary research articles, which also contributed to the outcome of a high risk of bias in several publications. However, in many cases, this was not a requirement by scientific journals at the time of publication.

The overall outcome of a high RoB for all studies emphasises that studies conducted on the effectiveness of BSMs often fail to reach the required quality, and reported results are problematic when it comes to a true meta-analytical approach. This may stem from the need for mandatory preregistration and protocol preparation in observational studies, leading to publication bias and selective outcome reporting. Moreover, the high RoB also reflects the absence of harmonised study designs and insufficient empirical evidence to support causal relationships between BSMs and pathogen reduction.

### 4.2 Generalisations and cross-protective effect of some BSMs across pathogens

The leading and potentially most effective BSMs for *Salmonella* spp. and HEV were all related to preventing indirect contact between pig herds. Indirect contact between herds is possible via persons, and the BSMs related to such indirect contact were either related to showering, wearing farm-specific clothes and/or boots, or a combination of these two BSMs. According to a review on biosecurity in pig production (13), hand-washing and outwear changes are probably effective for many pathogens to prevent transmission into a new herd. A higher level of biosecurity would include a compulsory shower (13). Experimental studies showed that showering and changing outwear could prevent Foot-and-Mouth disease virus transmission between infected and susceptible pigs (59). Nevertheless, a study in 2008 in Belgian pig herds reported that only 2% of pig farmers used showering before entering. This low percentage may apply to other countries as well (60). Since BSMs related to humans entering the farm have shown to be effective for multiple pathogens (38, 59), complete showering and changing outwear could be advised to farmers aiming to improve their level of biosecurity.

Acidification of feed and water was found as a protective measure in relation to *Salmonella* spp. presence on farms. However, these measures are only relevant if *Salmonella* is already present, yet they cannot prevent the introduction of *Salmonella* on farms. Therefore, when veterinarians use the current meta-analysis to advise their clients about *Salmonella* control, they must first assess whether the focus must be on prevention of introduction or reduction in prevalence. If the primary focus is on preventing *Salmonella* introduction, a farm-specific assessment of potential breaches in external biosecurity for all production stages is advised. The effectiveness of acidification to prevent HEV infection is irrelevant, as viruses cannot colonise tissues, and HEV primarily infects hepatocytes of the liver, instead of enterocytes of the gastrointestinal tract like *Salmonella* spp. (61).

#### 4.2.1 Counterintuitive associations

While cleaning and disinfection are established, parts of *Salmonella* spp. control, when aggregating the data we found, there was no evidence of a positive effect on *Salmonella* spp. prevalence in pens/farms. The forest plot (Supplementary Figure S3B) revealed a split amongst the observations: four indicated a statistically non-significant protective effect, while four suggested disinfections as a risk factor. The two observations with the largest sample size belong to the four observations identifying disinfection as a risk factor. However, these two observations came from the same publication (S_MA12 (45)) and are therefore not independent. The following issues contributed to the fact that the aggregated data on disinfection only provided an inconclusive result. Of the six articles investigating the role of disinfection on *Salmonella* spp. prevalence on farms, only one actively detailed the procedure for disinfecting pens in their study. As many of the articles also relied on farmers’ self-reporting their protocols, the introduction of information bias may explain the results of these studies.

### 4.3 Limitations and strengths

#### 4.3.1 Limitations

This systematic review identified biosecurity measures (BSMs) likely to reduce *Salmonella* spp. or HEV occurrence on farms. However, many BSMs were supported by only a single observation in one article, preventing an accurate assessment of their true effectiveness due to potential bias in included studies. The scarcity of observations per BSM in the literature may be due to publication bias, favouring the publication of novel findings over studies that confirm or refute existing evidence (11). Also, BSMs incorporated in multiple studies may have been assessed differently per study (i.e., the question or answer options were different), leading to some variation of the results between studies. Finally, it is essential to acknowledge that some information on BSMs against our pathogens of interest may have been overlooked for several reasons. For instance, this study considered only peer-reviewed publications and only articles available in the native language of at least one of the study authors, which may cause an information bias for “non-usual” European languages. Additionally, case reports describing the effect of depopulation in eradicating a pathogen from a herd (62) were not included in the meta-analysis as the effect of this measure could not be quantified despite depopulation being potentially a highly effective measure (63). Moreover, publication bias may have resulted in missing data on the ineffectiveness of certain BSMs. This bias can occur in meta-analyses and literature reviews, leading to gaps in knowledge. By restraining our literature search to only published and peer-reviewed articles, we aimed to target high-quality research. However, this approach may have excluded unpublished data or grey literature, potentially leading to an overrepresentation of positive findings, as negative results are less likely to be published in peer-reviewed journals.

#### 4.3.2 Strengths

In systematic quantitative reviews and meta-analyses, RoB assessment is crucial but often absent in traditional narrative or scoping reviews, representing the vast majority of publications on farm biosecurity. This study is likely the first to include a RoB assessment of primary studies on specific BSMs. It adds a layer of scrutiny to their effectiveness in reducing on-farm pathogen occurrence. Additionally, this review introduced an innovative use of AI software for screening titles and abstracts, alongside manual reviewing, to reduce the time to screen articles without losing screening sensitivity. ASReview has been used in more than 10 systematic reviews since its launch in 2021 (64). The decision about when to stop screening abstracts using ASReview was recently investigated and was proposed to be carried out after at least 10% of the abstracts is screened and 50 consecutive abstracts are marked as irrelevant by the reviewer (21). Our screening of 13% of abstracts and stopping after 100 irrelevant abstracts can therefore be seen as a safe and conservative approach, minimizing the risk of missing relevant articles. Although the sensitivity of the method depends on the clarity of the research question, the choice of relevant articles at the start of the reviewing process, and the reliability of the researcher that does the screening, we also traditionally screened all articles, so the articles that may have been missed using the AI software could be included using the other method. Additionally, research has shown that medical scientific researchers also falsely in- and exclude almost 11% of studies, showing that traditional reviewing is not better *per se* (65). Our combination of the two methods may be ideal.

Majority of the final publications in our study were observational studies, which were inherently prone to bias and confounding. This impaired assessing a causal relationship between intervention measures and pathogen reduction (66, 67).

However, through the rigorous application of the RoB methodology (see Methodology section for comprehensive details), the conclusions drawn from our study maintained their robustness and validity.

### 4.4 Recommendations and future outlook

In general, the literature examined here provided limited evidence-based knowledge about the control of *Salmonella* spp., its introduction, and its spread on farms. There remains a lack of scientific evidence on areas such as the method of animal purchase or feed storage. These cannot be supplemented with findings on protection from HEV, for which evidence was even more scarce. The categorisation of the effectiveness of BSMs in this review helped to highlight which measures should be investigated further as a matter of top priority. In addition, accurate reporting of the methodology and results of experimental and observational studies examining the efficacy of BSMs against pathogens on farms would further improve the evidence base and allow for further prioritisation of specific BSMs.

This review, highlights the following:

- There was a lack of knowledge of BSMs, particularly against HEV.
- Further research should examine two of the BSMs found to be non-significant in the meta-analysis of the “ignoring production stage” approach, that is, all-in and all-out production and disinfection for *Salmonella* spp. This may settle the question of whether these BSMs are truly effective, while requiring less effort compared to other BSMs with less data available for evidence synthesis.
- The cost-effectiveness of implementing food and water acidification, based on systematic and standardised application of acids, as well as rodent control measures on farms, should also be assessed urgently to provide farmers with the knowledge and tools for implementation.
- Available literature often lacks quality and carries a high bias risk. Future research on BSMs in swine production and peer-review processes should prioritise standardised reporting. To further improve the scientific evidence regarding (farm) animal and zoonotic diseases epidemiology and the effectiveness of respective BSMs, standardized/harmonized study designs based on guidelines (including checklists such as COHERE (68) may foster the quality of studies in this field, reduce risk of bias and facilitate comparability of BSMs between studies). Additionally, using guidelines such as STROBE-vet (69) will help reduce bias when reporting the study design and participant selection.

In summary, further research is essential to assess BSMs’ efficacy in reducing pathogen burden on pig farms, enhancing food safety, and minimizing public health risks. Implementing studies within national control programs, enhancing sample sizes, and incorporating power-based estimates can improve evidence quality. This review also highlights the need for more studies on preventive measures as well as field and experimental studies that can confirm the protectiveness of the BSMs that have not been covered in this review. For instance, comparing various cleaning and disinfection methods and addressing water contamination within farms.

Moreover, it is worth mentioning that the results of this literature review were used to generate two self-assessment checklists that aim to support pig farmers in identifying possible gaps in their on-farm implemented biosecurity towards *Salmonella* spp. and HEV. These checklists are open-access and available in eight European languages (70).

In conclusion, our systematic review and meta-analysis emphasise the importance of dedicating more effort and conducting further research to truly understand the effectiveness of BSMs against zoonotic pathogens in swine farms. This study provides a starting point by identifying BSMs that should be given priority for additional investigation, as well as those that could offer benefits for use on farms.

## Data Availability

The original contributions presented in the study are included in the article/Supplementary material, further inquiries can be directed to the corresponding author/s.
