# Supplementary figures and images for: Biosecurity measures reducing Salmonella spp. and hepatitis E virus prevalence in pig farms—a systematic review and meta-analysis
Source: Front Vet Sci. 2024 Dec 23;11:1494870. doi: 10.3389/fvets.2024.1494870 (PMC11701885; doi:10.3389/fvets.2024.1494870)

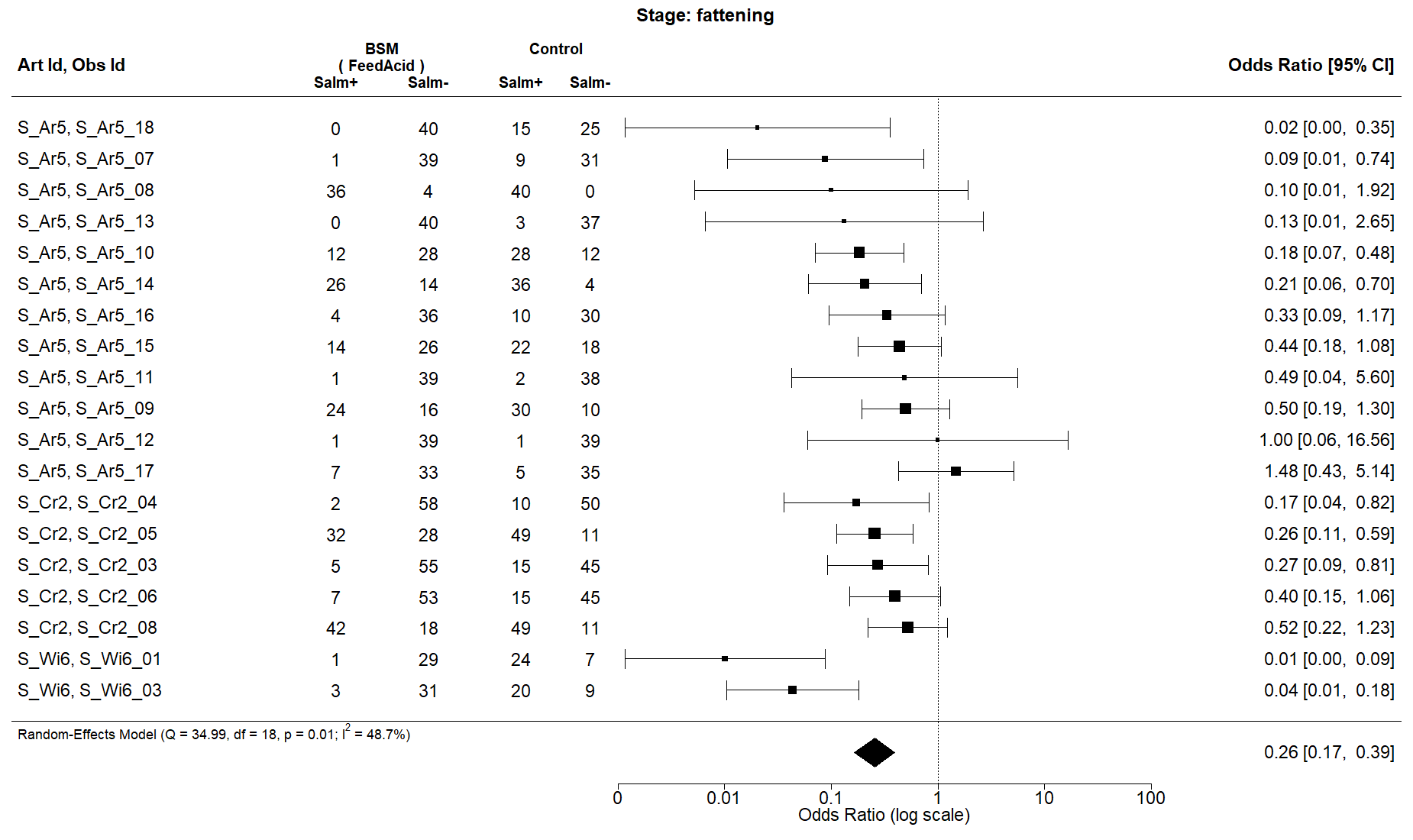

Supplement: Supplementary file 1 [file Data_Sheet_1.zip › ConsiderStage 5 or more obs/Forest_FeedAcid_with_stage.png]

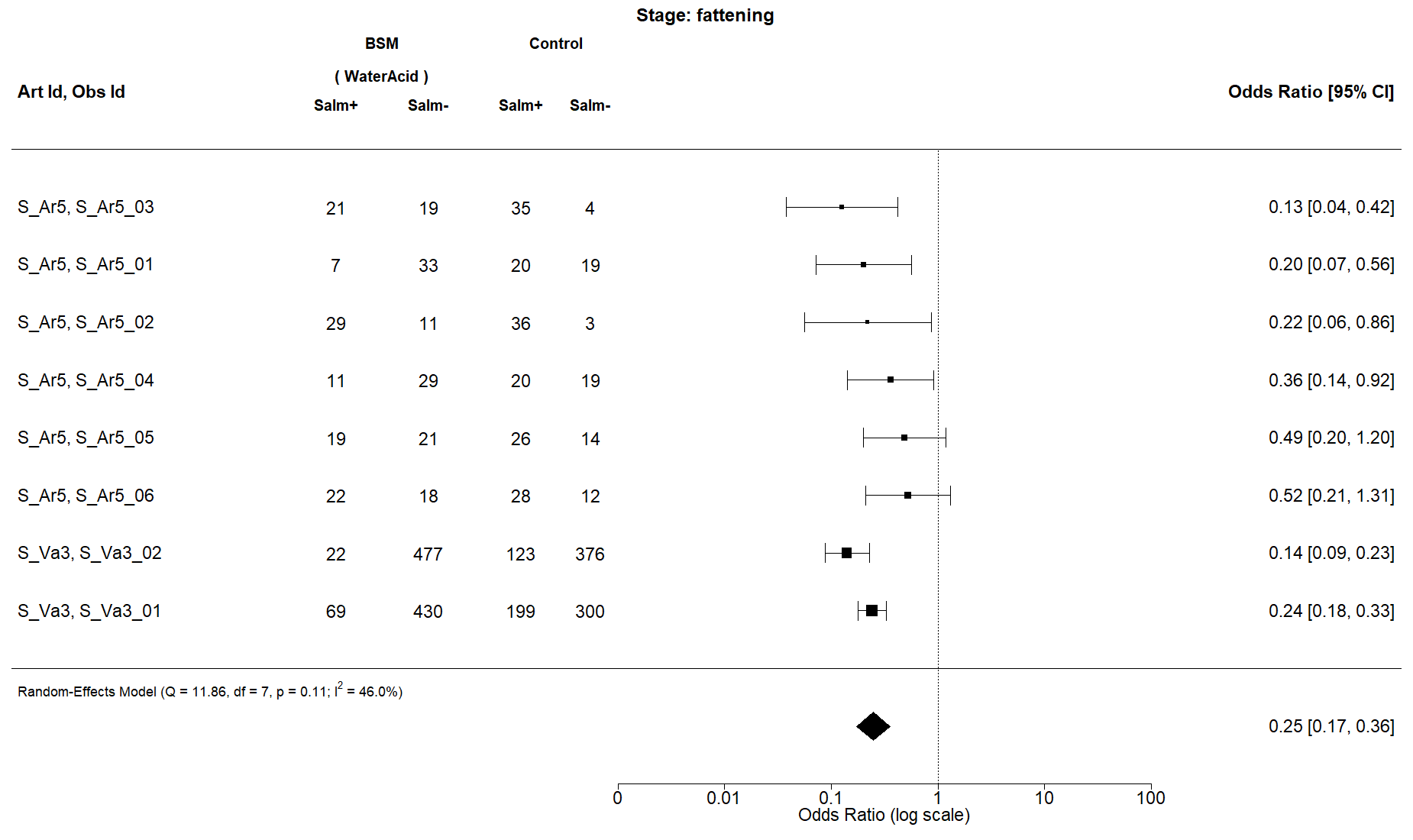

Supplement: Supplementary file 1 [file Data_Sheet_1.zip › ConsiderStage 5 or more obs/Forest_WaterAcid_with_stage.png]

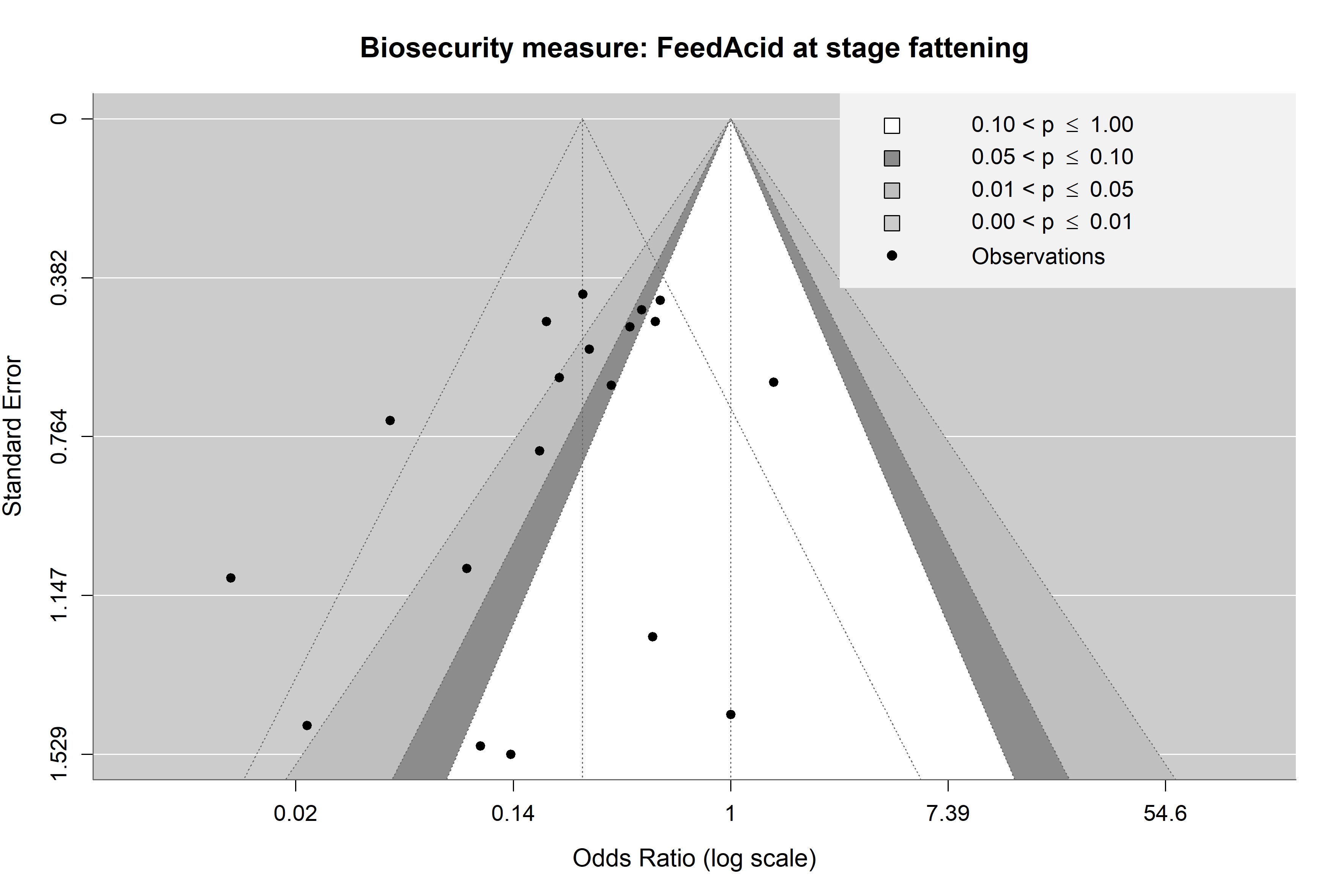

Supplement: Supplementary file 1 [file Data_Sheet_1.zip › ConsiderStage 5 or more obs/Funnel_FeedAcid_at_stage_fattening.png]

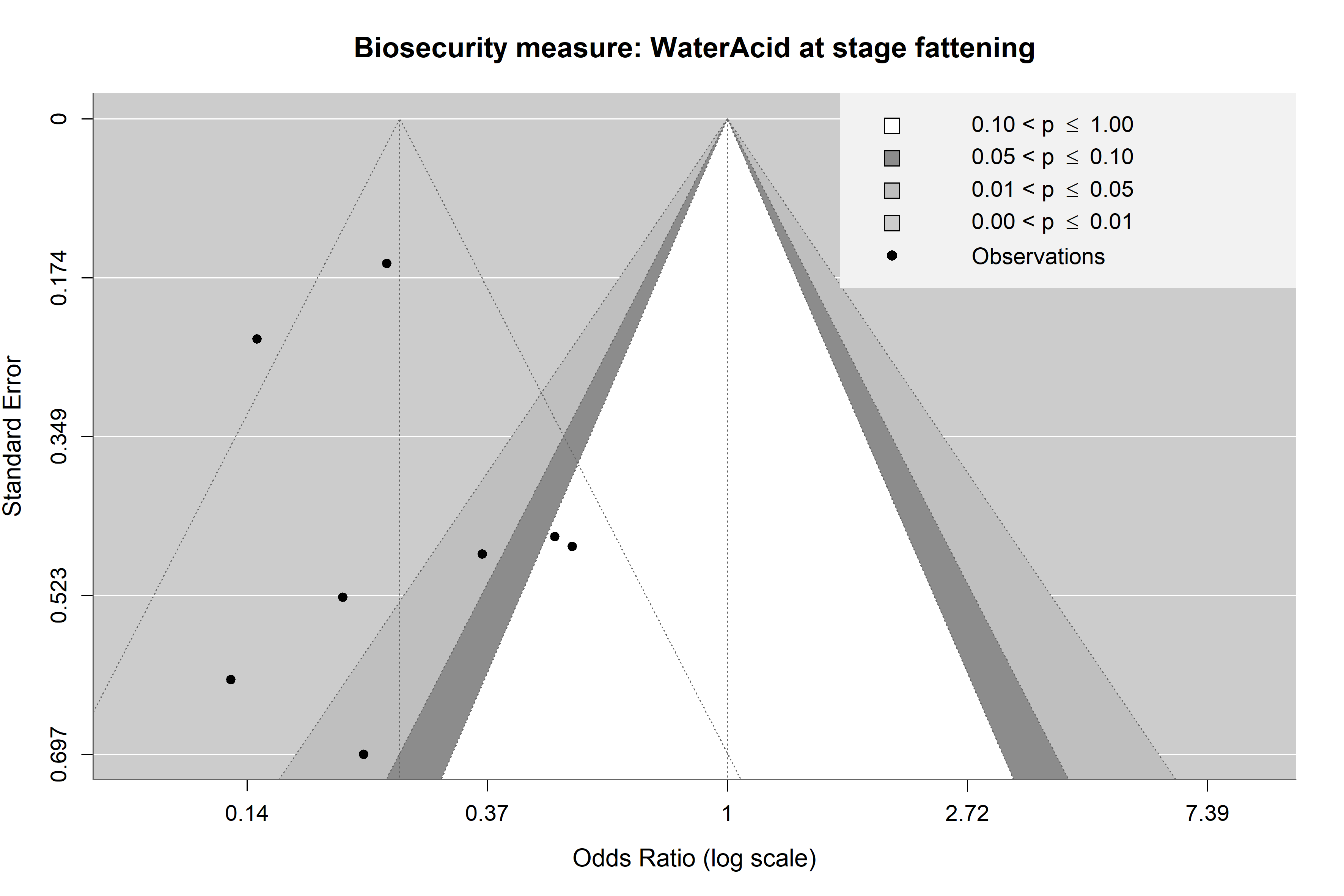

Supplement: Supplementary file 1 [file Data_Sheet_1.zip › ConsiderStage 5 or more obs/Funnel_WaterAcid_at_stage_fattening.png]

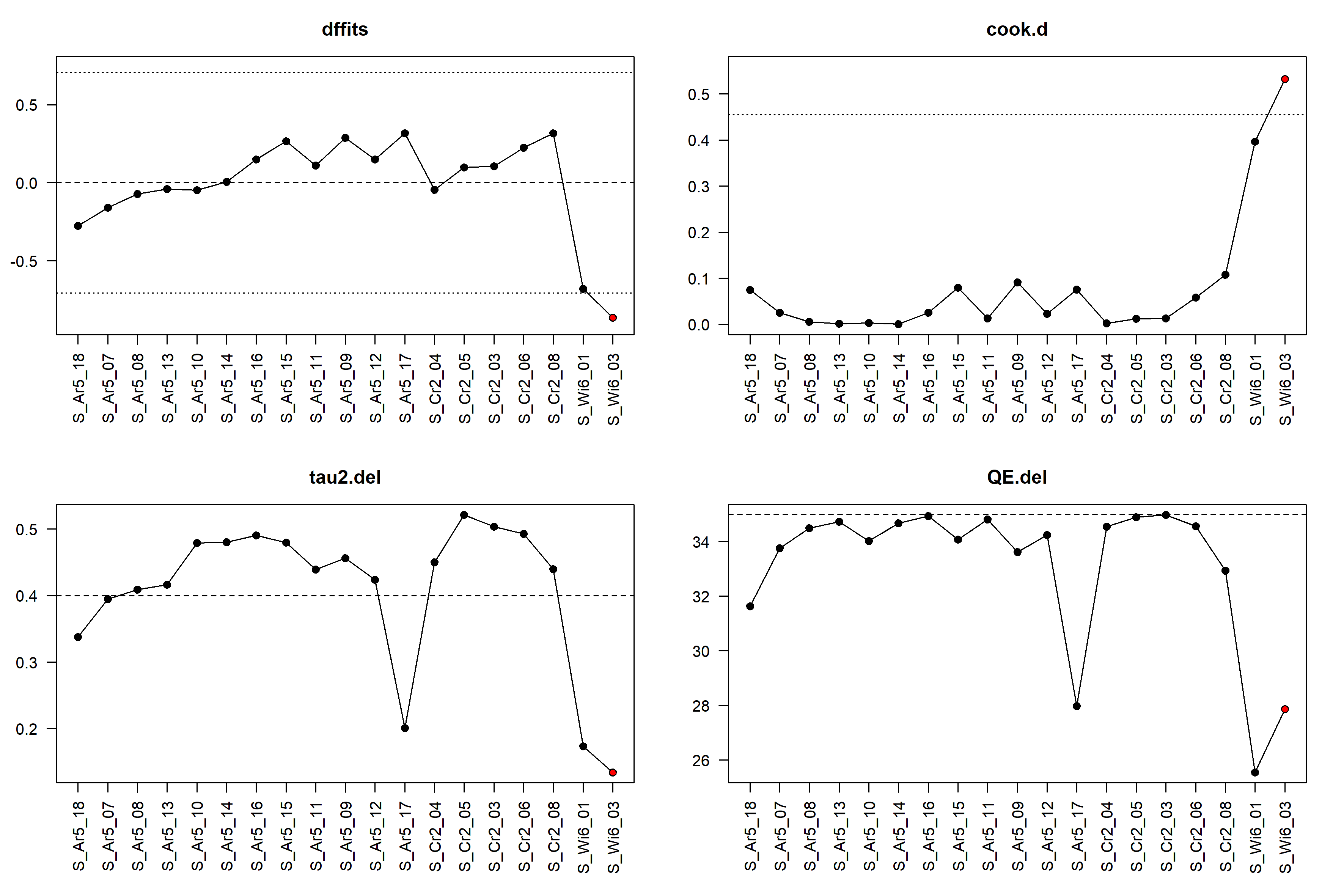

Supplement: Supplementary file 1 [file Data_Sheet_1.zip › ConsiderStage 5 or more obs/Sensitivity_FeedAcid_at_stage_fattening.png]

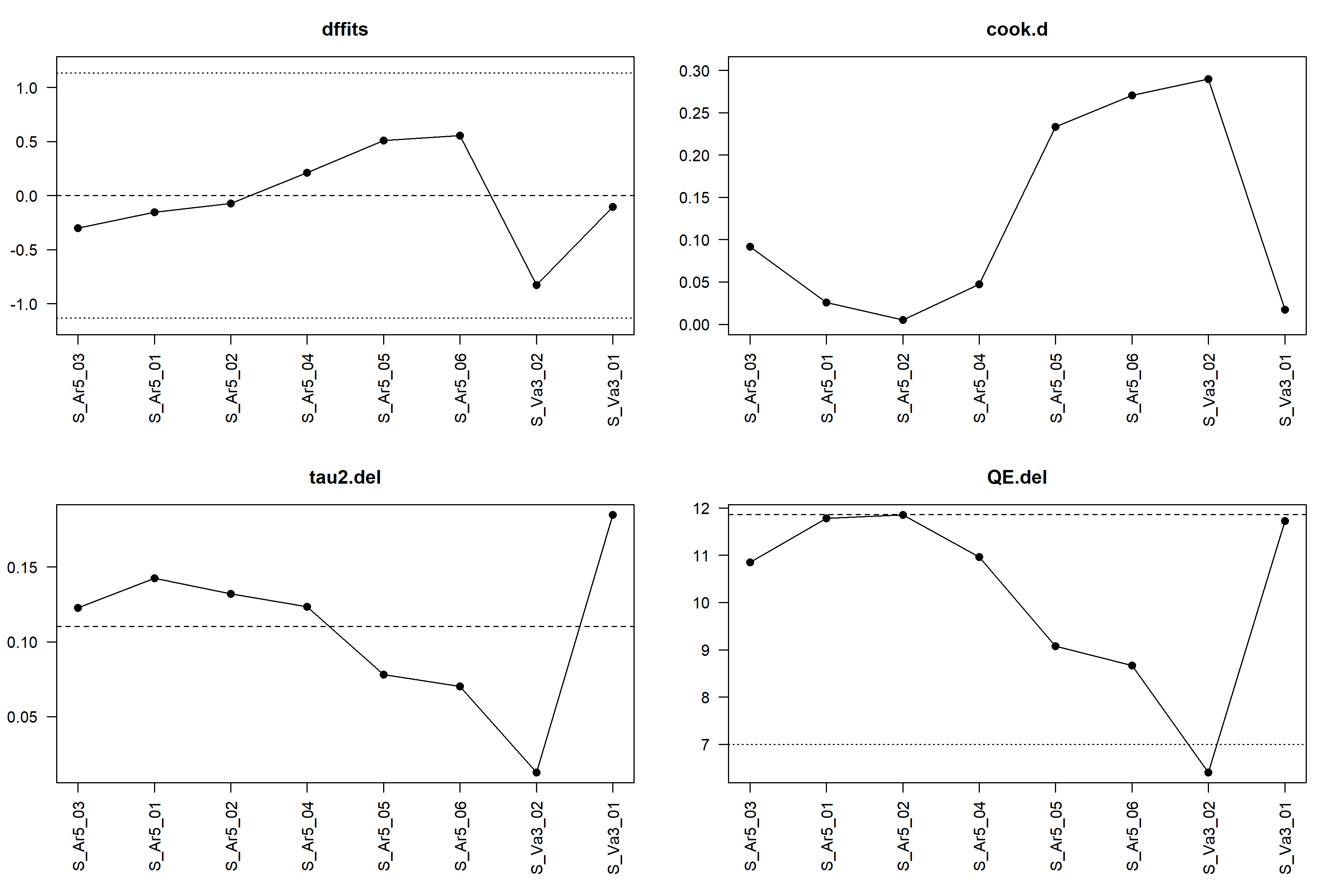

Supplement: Supplementary file 1 [file Data_Sheet_1.zip › ConsiderStage 5 or more obs/Sensitivity_WaterAcid_at_stage_fattening.png]

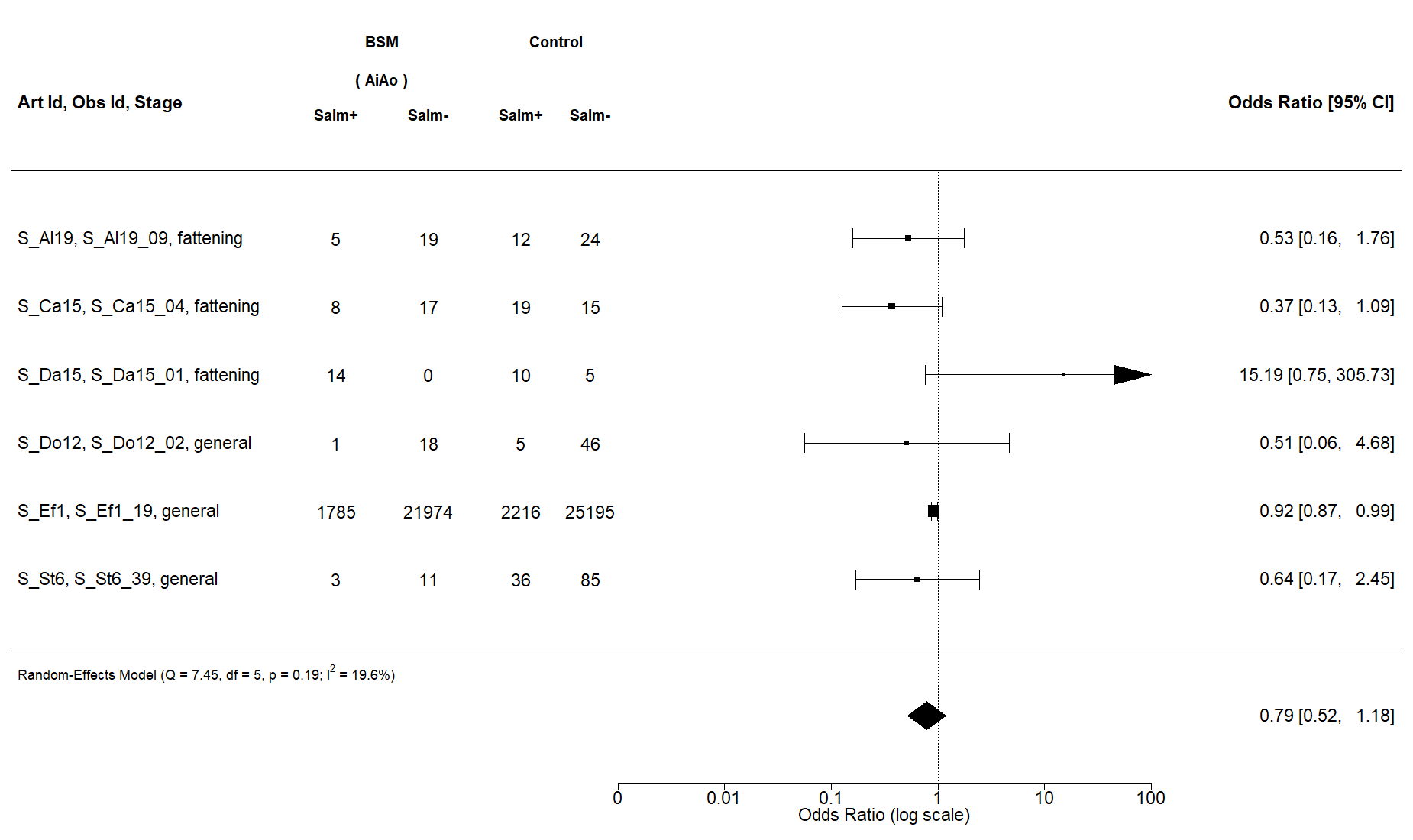

Supplement: Supplementary file 1 [file Data_Sheet_1.zip › IgnoreStage 5 or more obs/Forest_AiAo_ignore_stage.png]

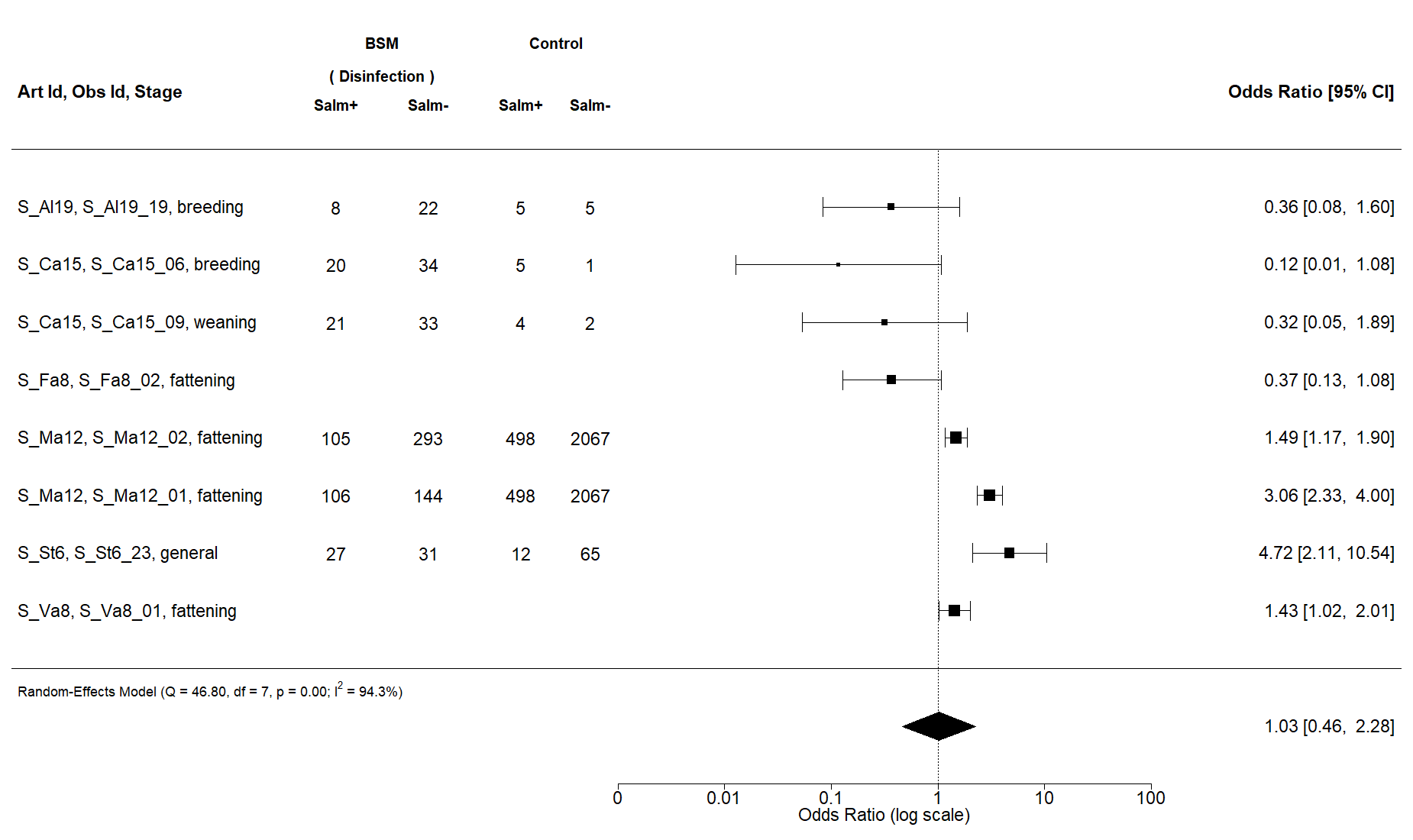

Supplement: Supplementary file 1 [file Data_Sheet_1.zip › IgnoreStage 5 or more obs/Forest_Disinfection_ignore_stage.png]

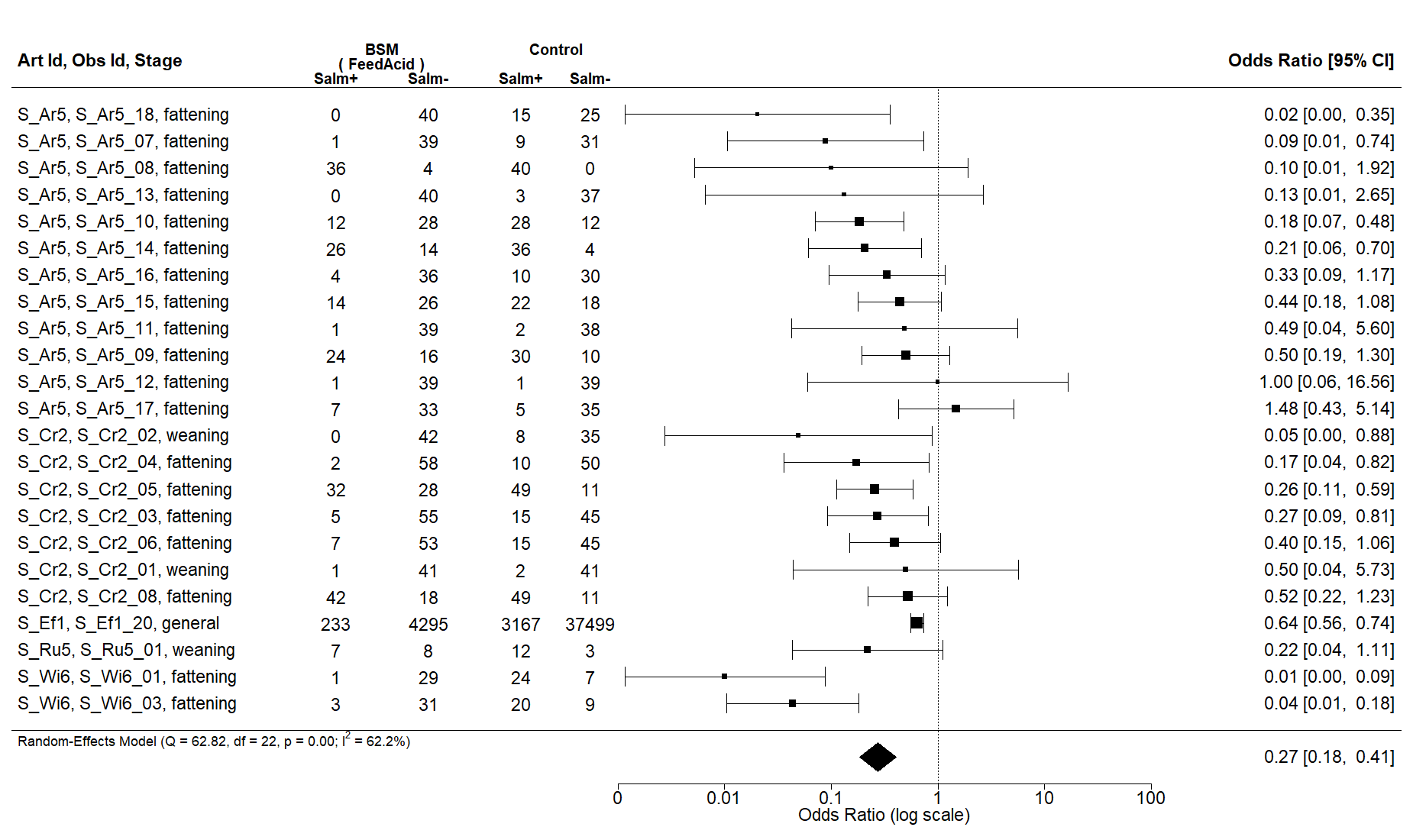

Supplement: Supplementary file 1 [file Data_Sheet_1.zip › IgnoreStage 5 or more obs/Forest_FeedAcid_ignore_stage.png]

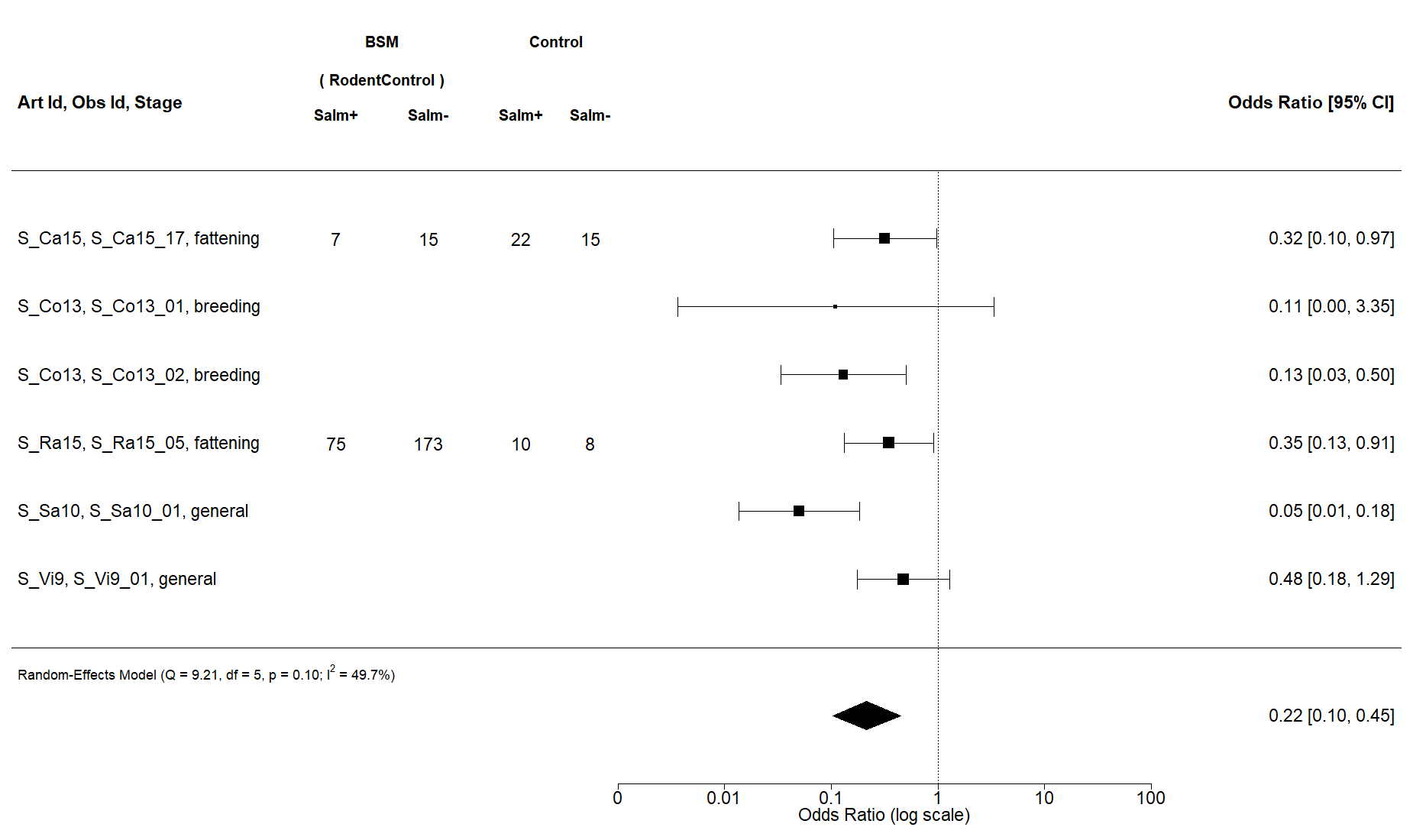

Supplement: Supplementary file 1 [file Data_Sheet_1.zip › IgnoreStage 5 or more obs/Forest_RodentControl_ignore_stage.png]

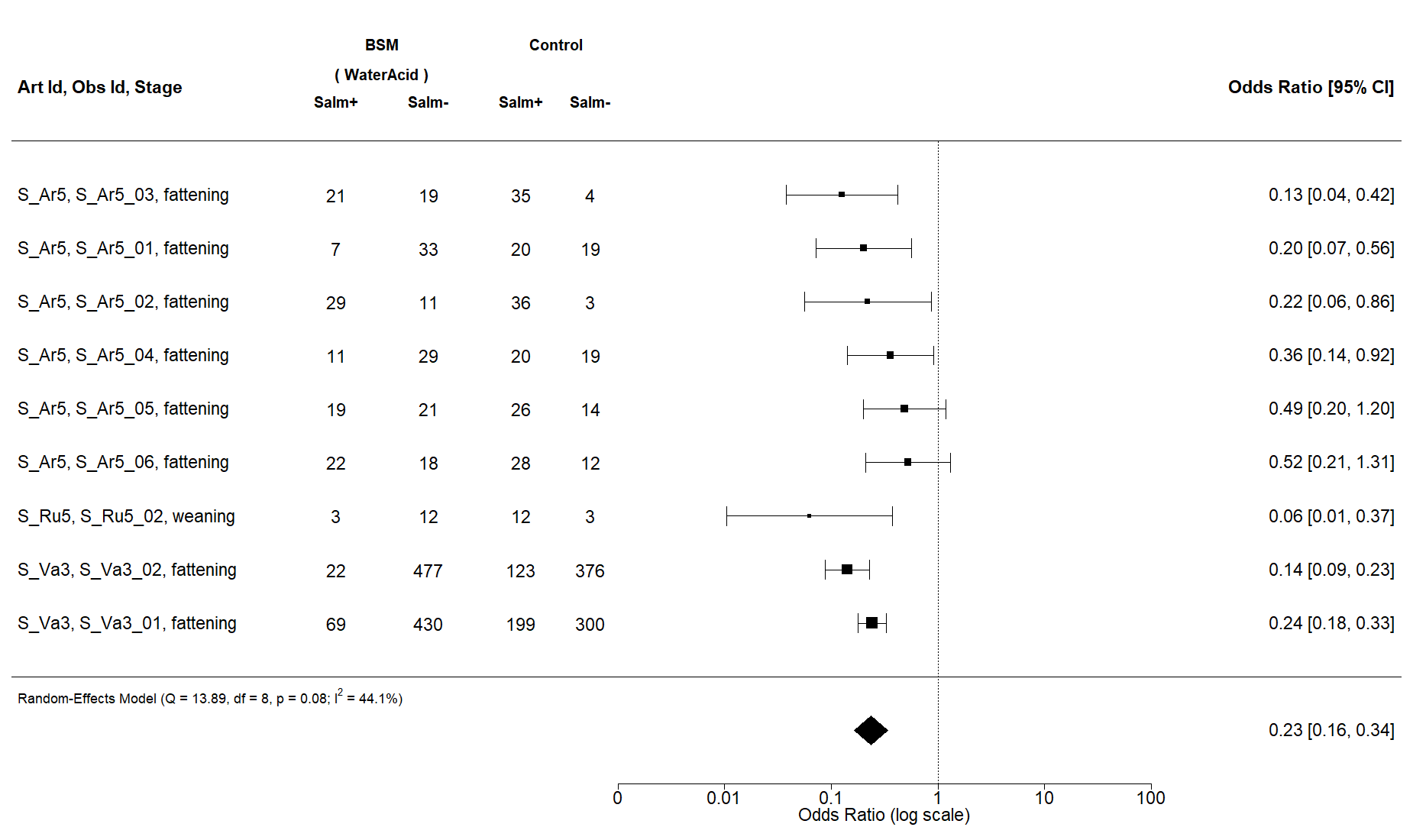

Supplement: Supplementary file 1 [file Data_Sheet_1.zip › IgnoreStage 5 or more obs/Forest_WaterAcid_ignore_stage.png]

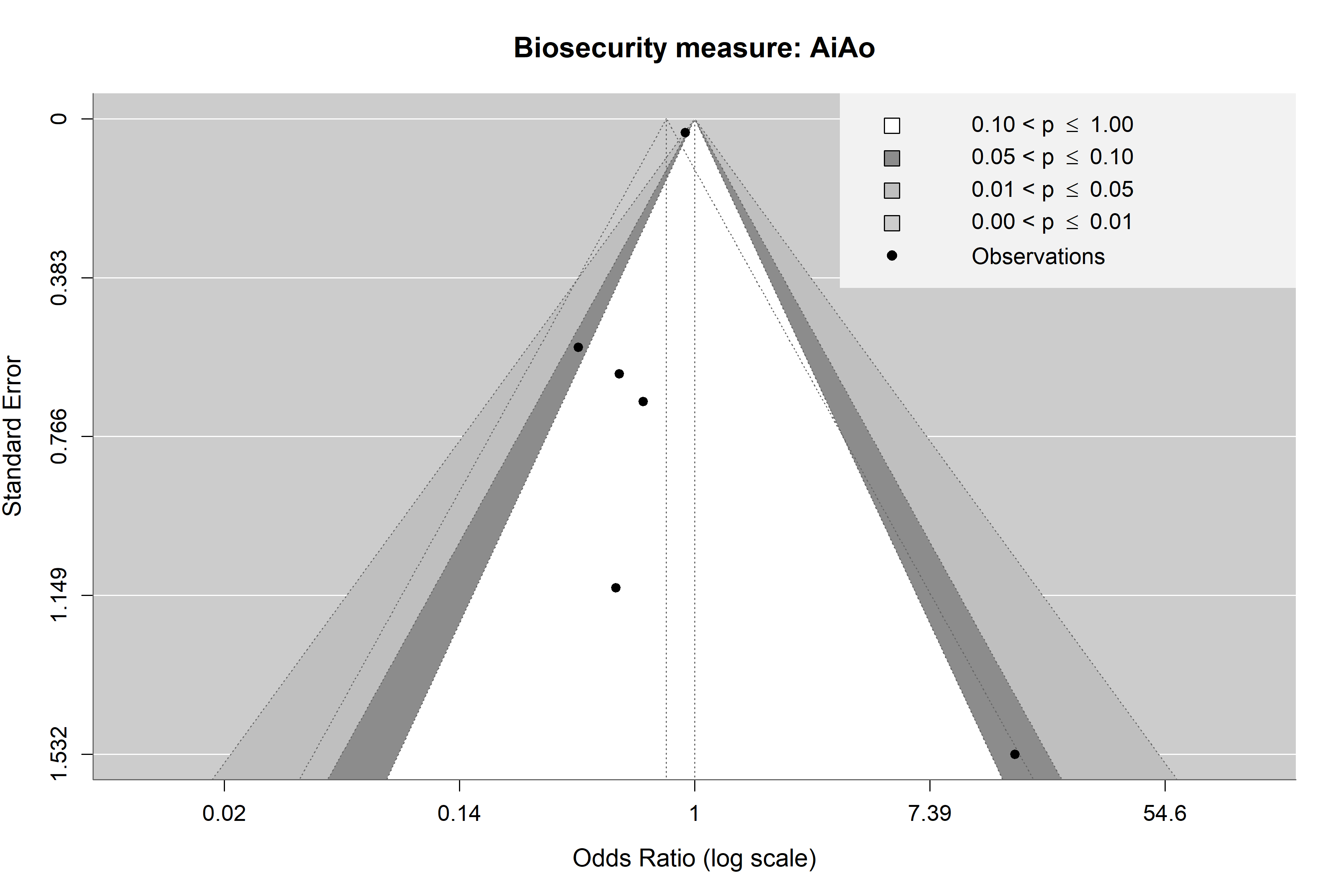

Supplement: Supplementary file 1 [file Data_Sheet_1.zip › IgnoreStage 5 or more obs/Funnel_AiAo_ignore_stage.png]

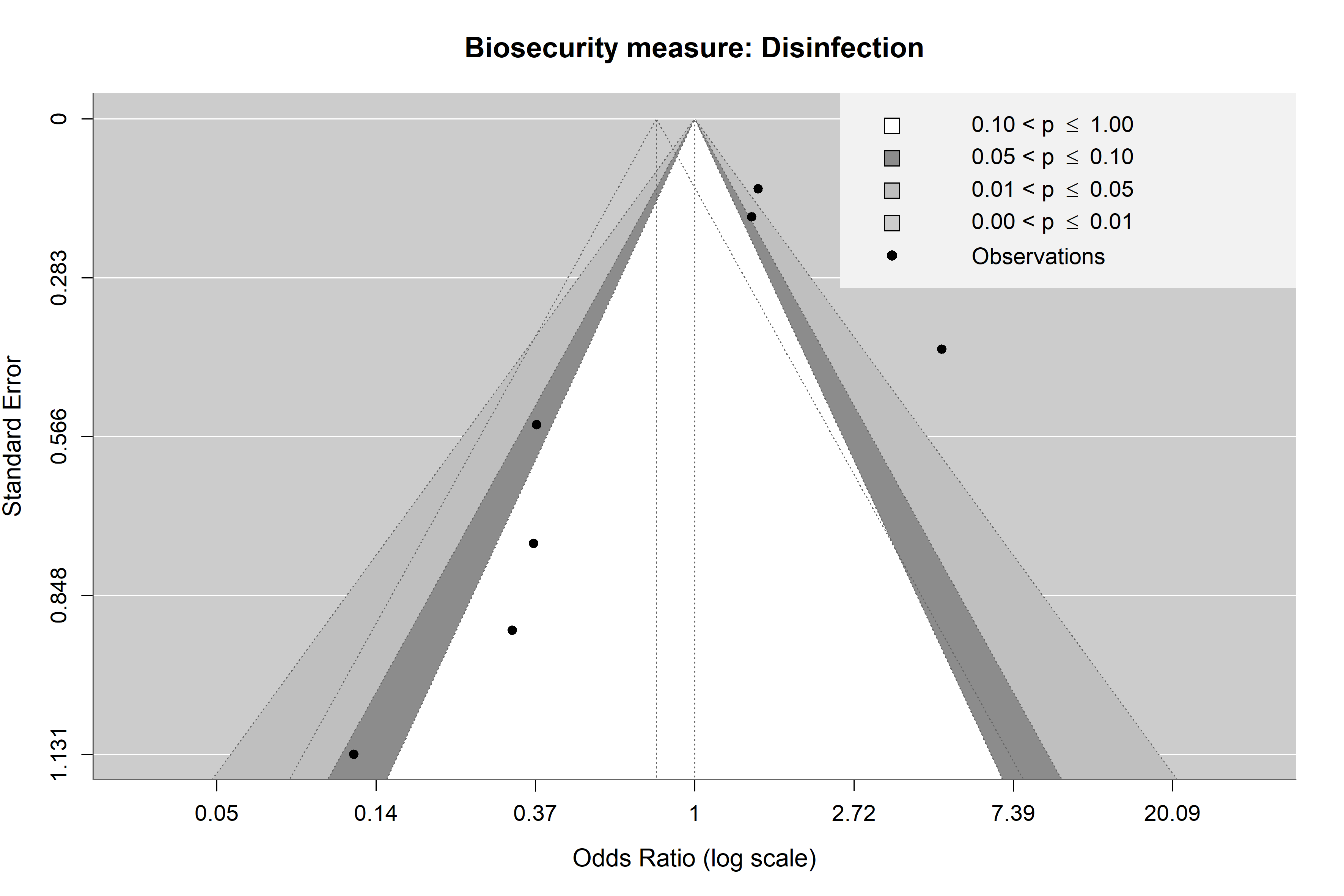

Supplement: Supplementary file 1 [file Data_Sheet_1.zip › IgnoreStage 5 or more obs/Funnel_Disinfection_ignore_stage.png]

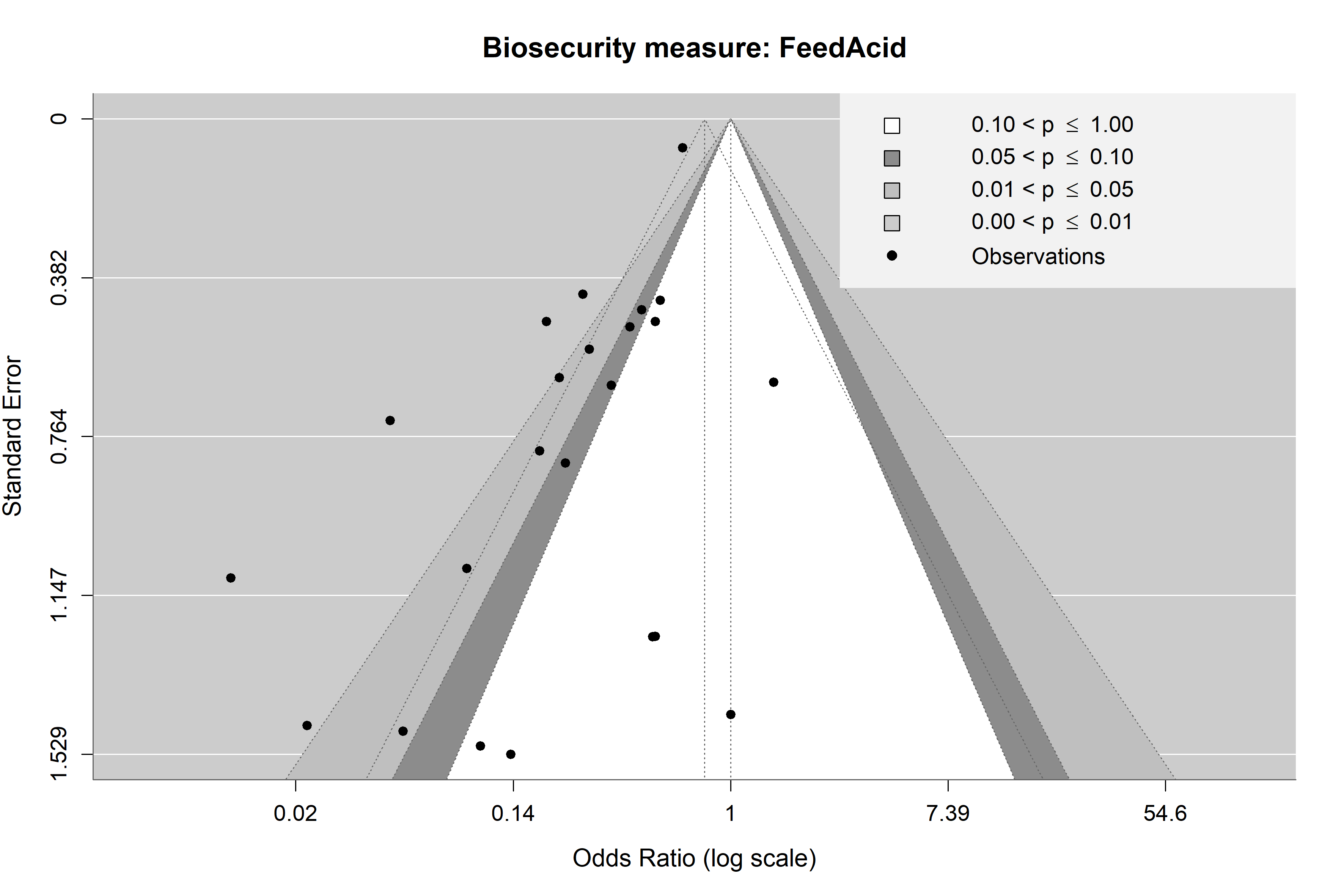

Supplement: Supplementary file 1 [file Data_Sheet_1.zip › IgnoreStage 5 or more obs/Funnel_FeedAcid_ignore_stage.png]

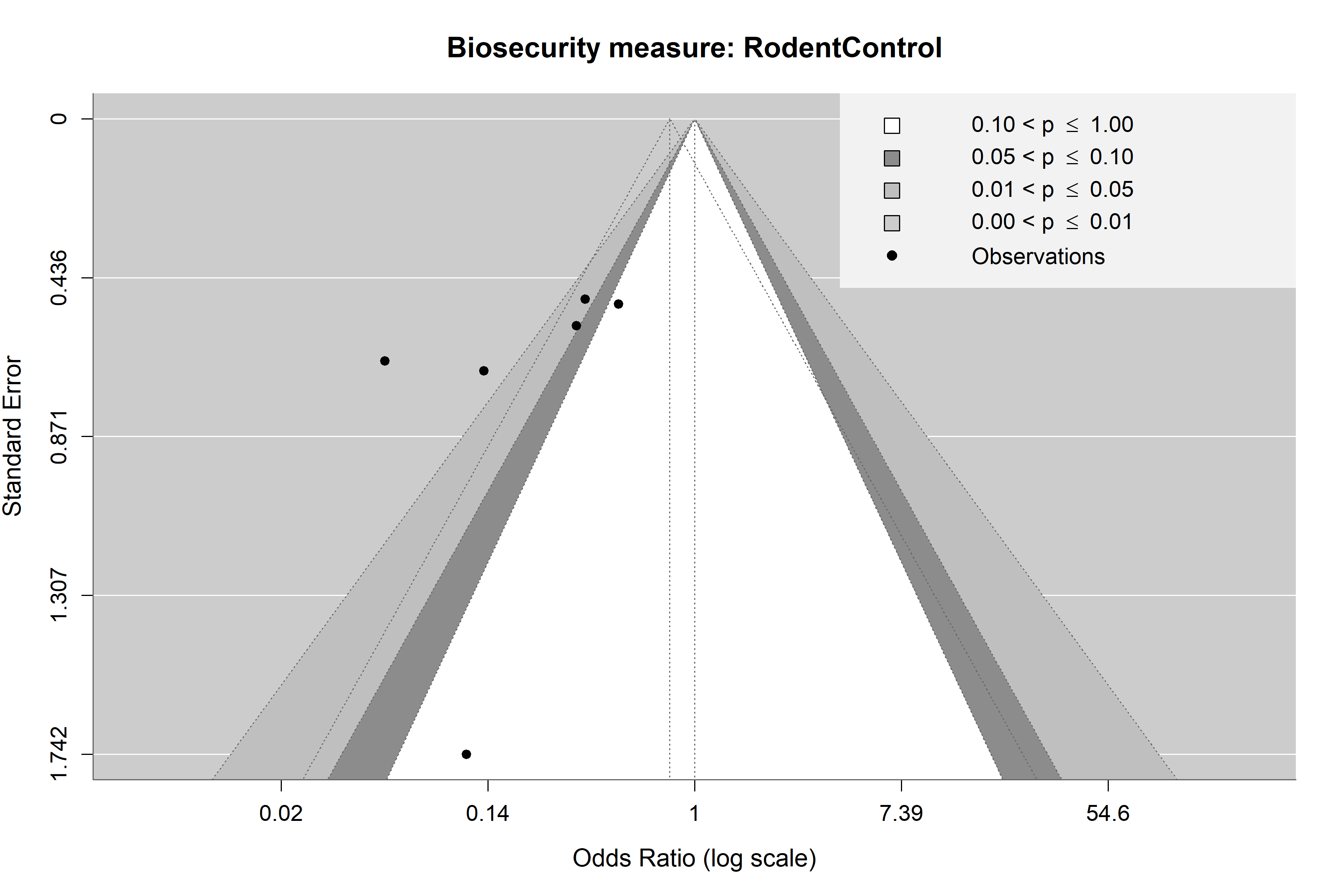

Supplement: Supplementary file 1 [file Data_Sheet_1.zip › IgnoreStage 5 or more obs/Funnel_RodentControl_ignore_stage.png]

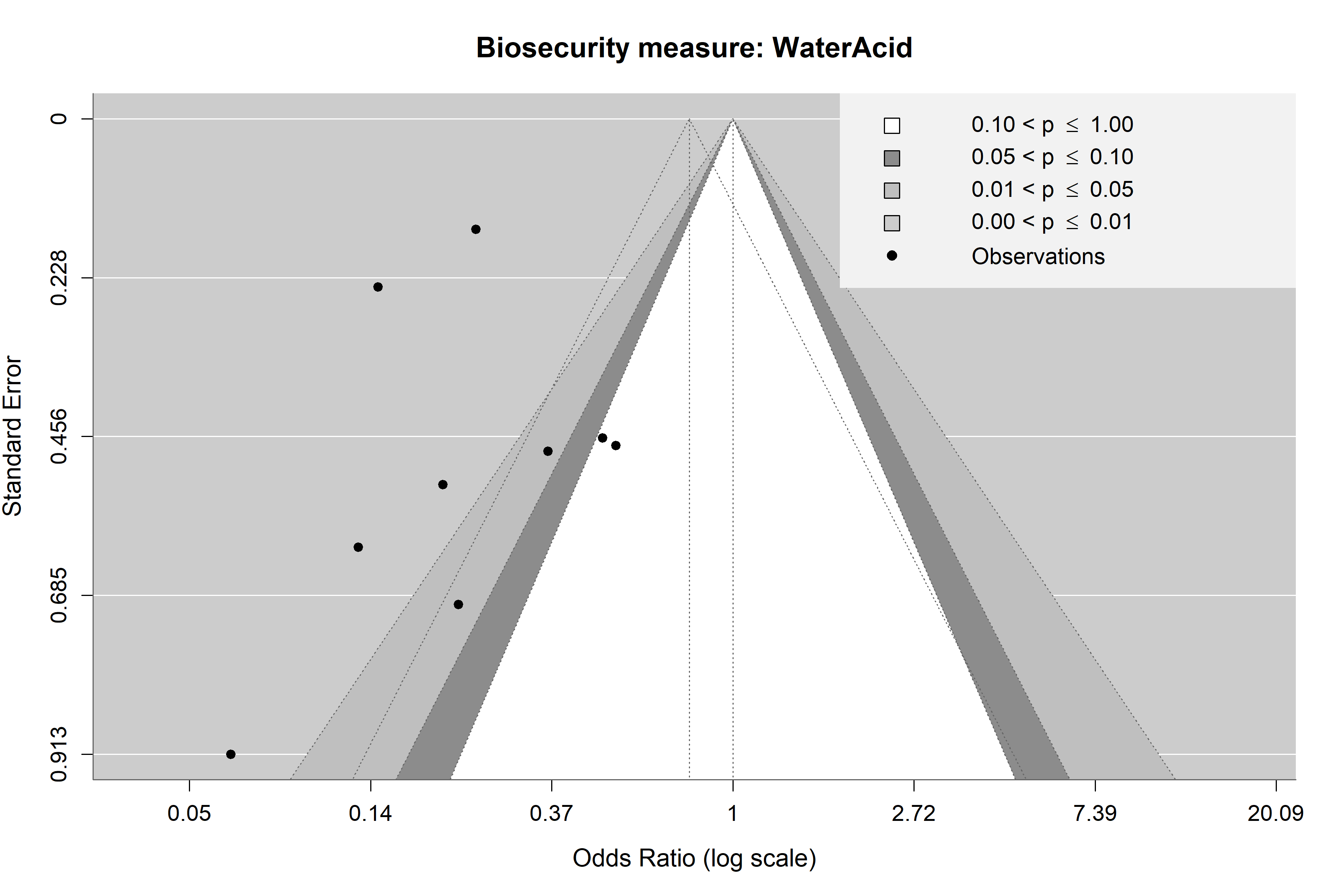

Supplement: Supplementary file 1 [file Data_Sheet_1.zip › IgnoreStage 5 or more obs/Funnel_WaterAcid_ignore_stage.png]

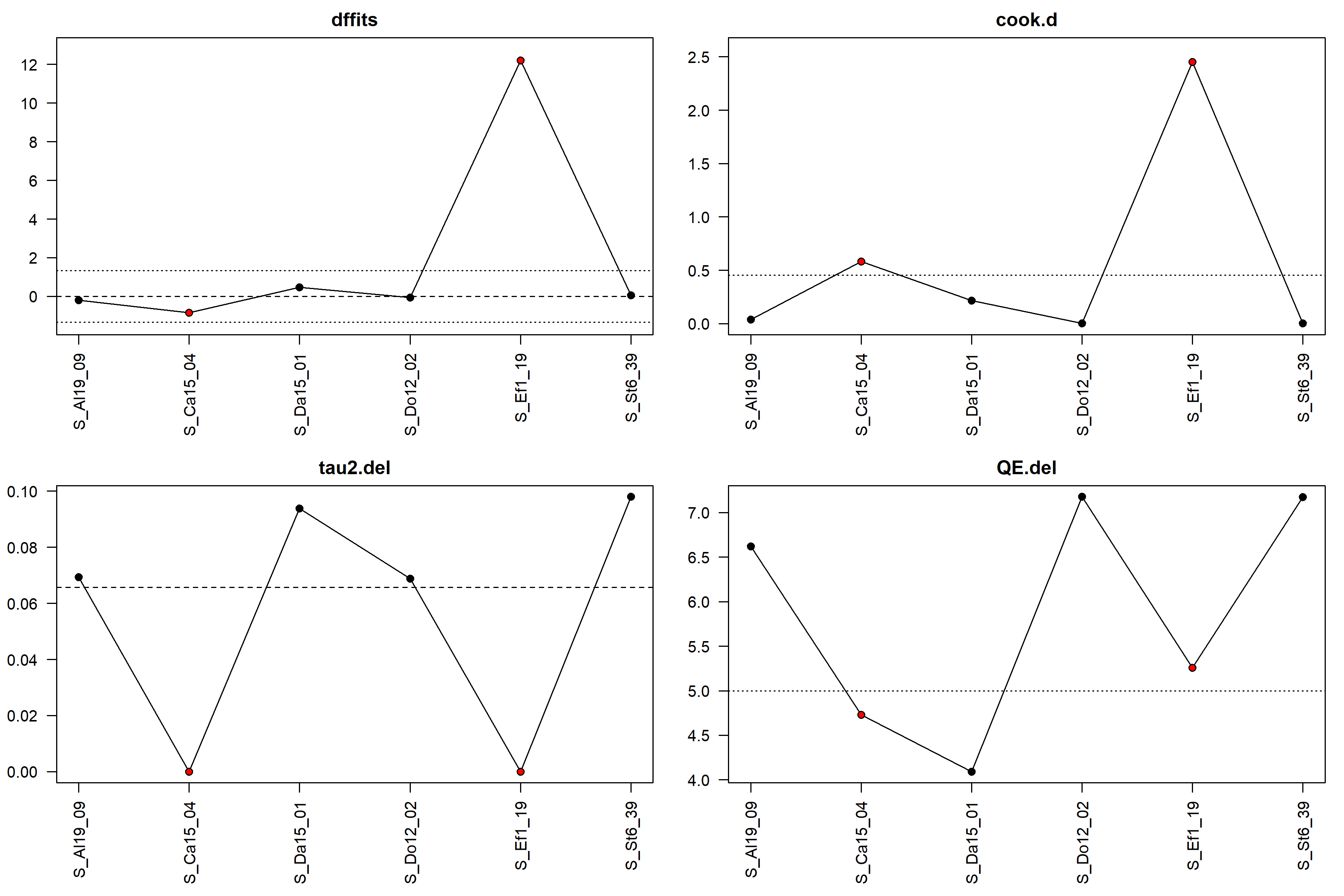

Supplement: Supplementary file 1 [file Data_Sheet_1.zip › IgnoreStage 5 or more obs/Sensitivity_AiAo_ignore_stage.png]

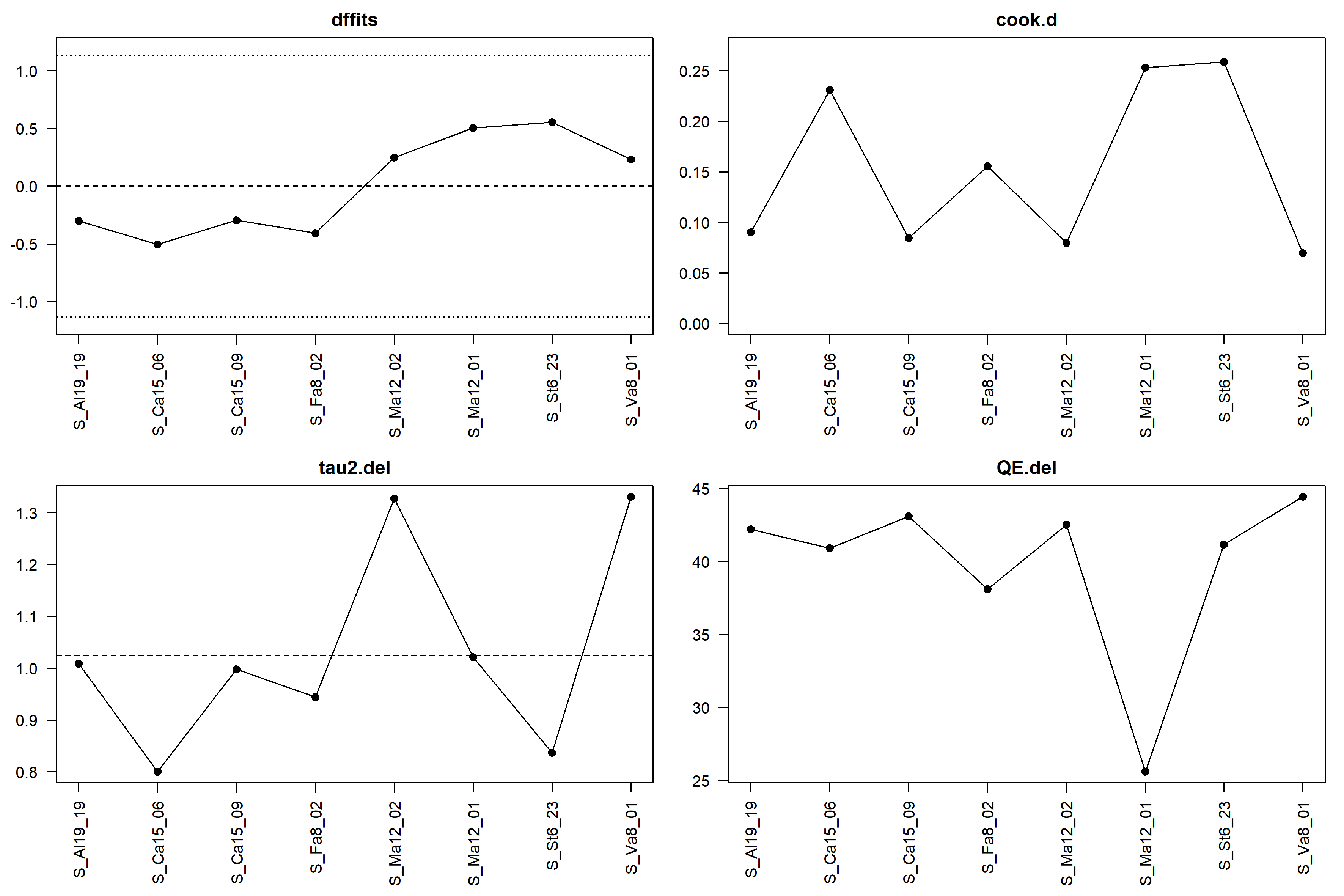

Supplement: Supplementary file 1 [file Data_Sheet_1.zip › IgnoreStage 5 or more obs/Sensitivity_Disinfection_ignore_stage.png]

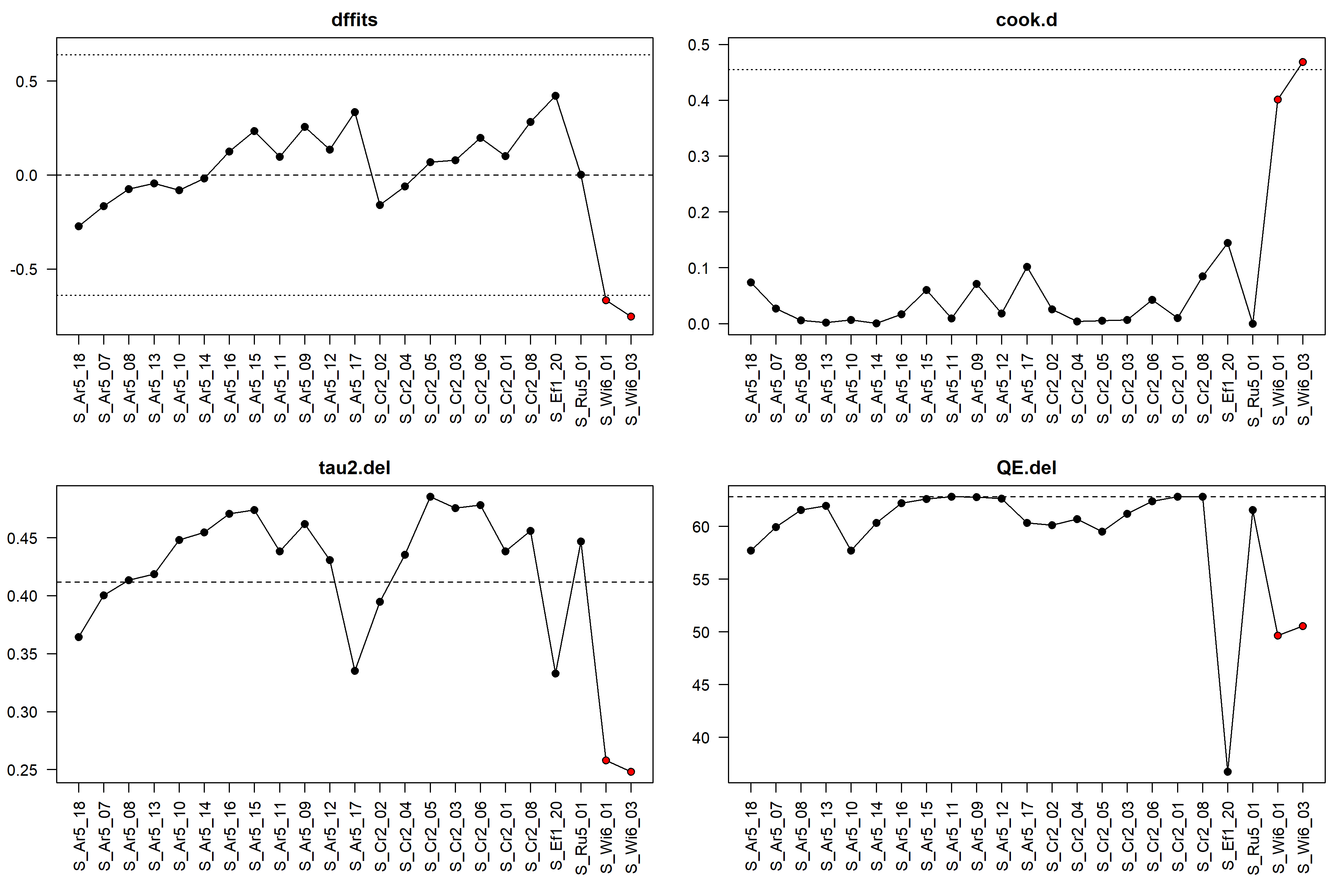

Supplement: Supplementary file 1 [file Data_Sheet_1.zip › IgnoreStage 5 or more obs/Sensitivity_FeedAcid_ignore_stage.png]

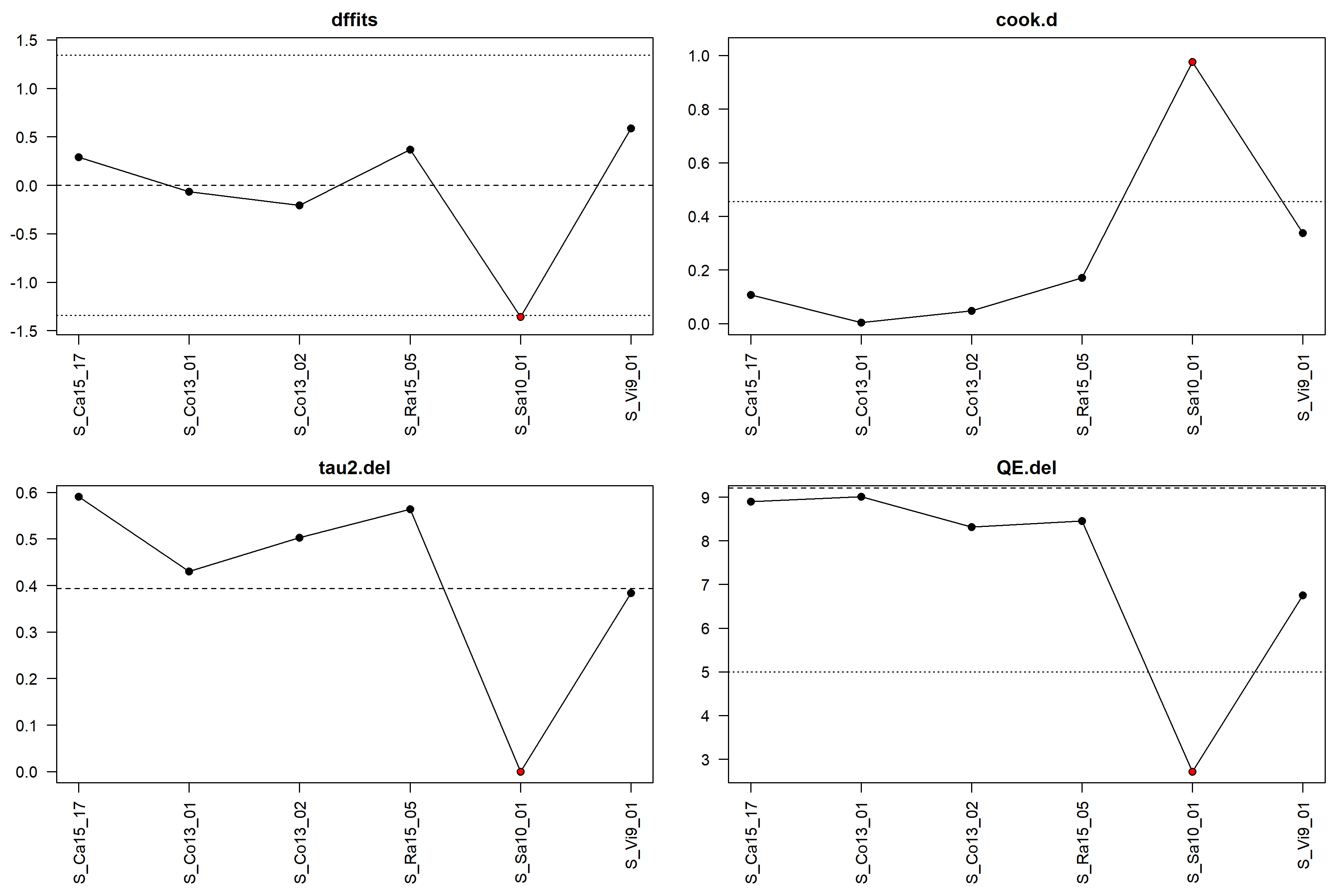

Supplement: Supplementary file 1 [file Data_Sheet_1.zip › IgnoreStage 5 or more obs/Sensitivity_RodentControl_ignore_stage.png]

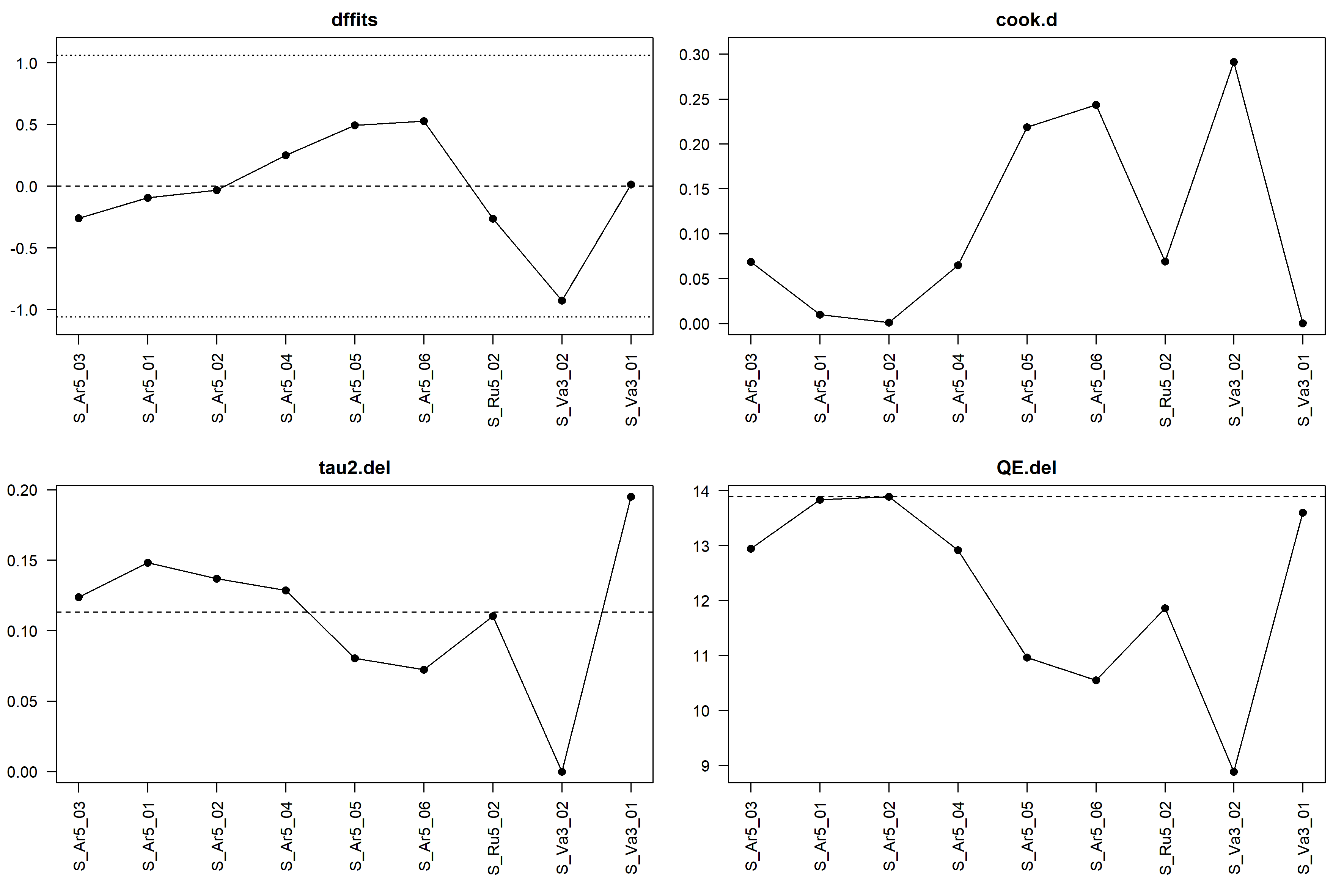

Supplement: Supplementary file 1 [file Data_Sheet_1.zip › IgnoreStage 5 or more obs/Sensitivity_WaterAcid_ignore_stage.png]

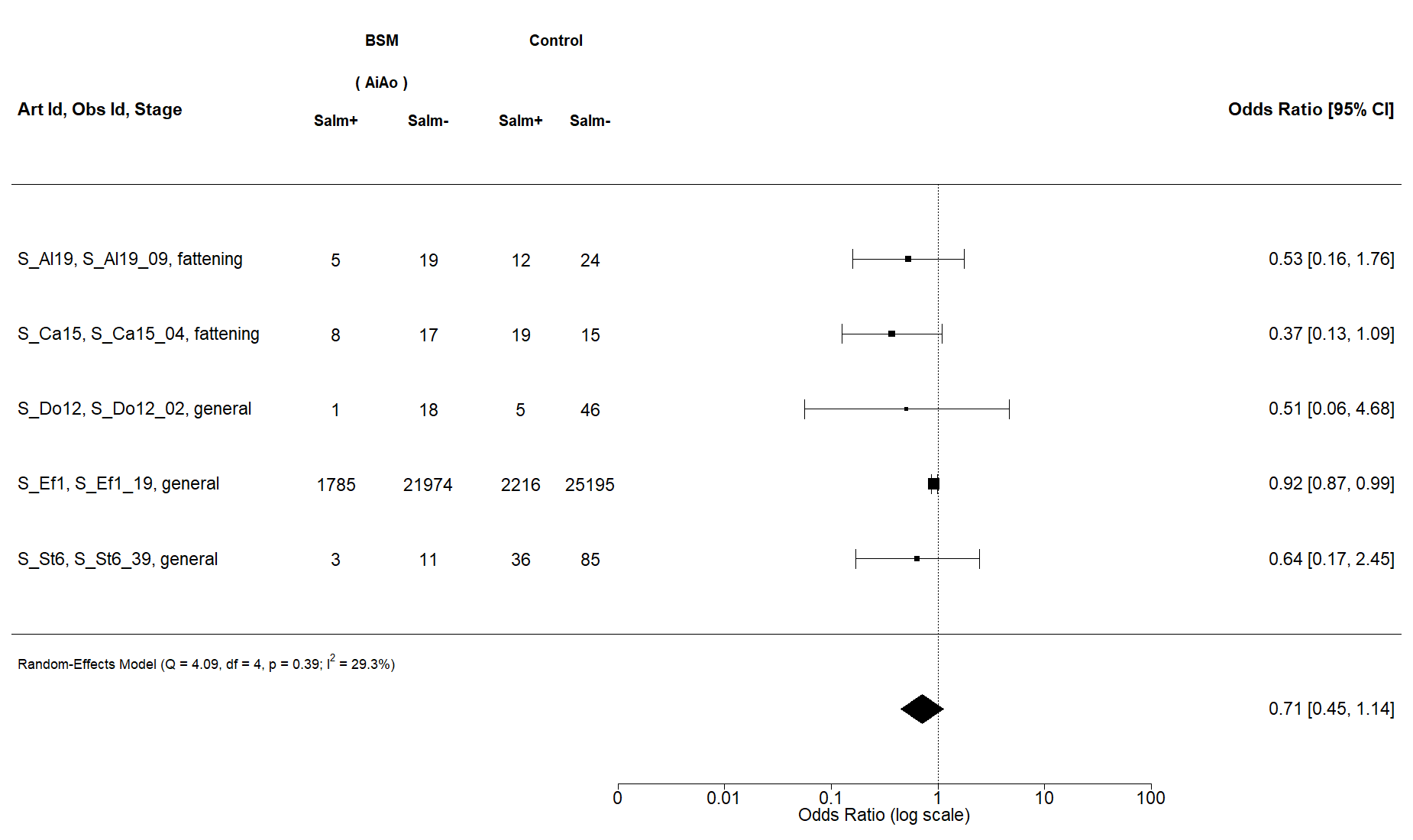

Supplement: Supplementary file 1 [file Data_Sheet_1.zip › IgnoreStage 5 or more obs_WITHOUT_S_Da15/Forest_AiAo_ignore_stage.png]
