# Supplementary material for: Biosecurity measures reducing Salmonella spp. and hepatitis E virus prevalence in pig farms—a systematic review and meta-analysis
Source: Front Vet Sci. 2024 Dec 23;11:1494870. doi: 10.3389/fvets.2024.1494870 (PMC11701885; doi:10.3389/fvets.2024.1494870)
Supplement: Supplementary file 2 [file Data_Sheet_2.docx]

# Supplementary Material for Huber et al. (2024) Biosecurity measures reducing Salmonella spp. and hepatitis E virus prevalence in pig farms: A systematic review and meta-analysis (Front. Vet. Sci. 11:1494870)

**Supplementary Table S1**: Keywords used in the search definition, and the search terms which were used in the final query submitted to the search engines. No time limits were applied to the search query.
^#^Only one pathogen was searched at a time across all three databases.

| **Search Terms** |
| --- |
|  |
| (Salmonella OR Salmonellosis) / (HEV OR “Hepatitis E”) |
| **AND** |
| (pig OR swine OR piglet OR sow OR boar OR farrow OR wean OR porcin* OR pork OR "sus scrofa domestica") |
| **AND** |
| (biosecurity OR hygien* OR "risk factor" OR intervent* OR prevent* OR reduc* OR biosafety OR "case control") |
| **OR** |
| (human OR vehicle OR purchase OR feed OR water OR bed OR mixing OR clean OR disinfect* OR equipment OR pest) |
| OR (stock worker OR animal keeper OR personnel OR staff OR visitor) |
| OR (other animal OR insect OR pet OR pest OR purchase OR carcass OR deadstock OR replace) |
| OR (transport OR truck OR feed deliver*) |
| OR (floor OR manure OR pit OR slat) |
| OR (boot OR glove OR fomite OR tool) |
| OR (water AND purification OR acidification OR source) |
| OR (feed AND storage OR quality OR supply) |
| OR (hygiene lock OR quarantine) |
| OR (all-in all-out OR pig-flow) |
| **AND** |
| (farm OR herd OR barn OR pig production OR operation) |
| **NOT** |
| vaccin* |
| **NOT** |
| review |
| **NOT** |
| (Asia OR South America OR Chine OR Vietnam OR Nigeria OR Phillipine OR Korea OR Argentina OR Brazil OR Thailand) |
| **NOT** |
| (Modelling OR Scenario) |

**Supplementary Figure 1**: The taxonomy used in BIOPIGEE organize the biosecurity measures (BSMs).


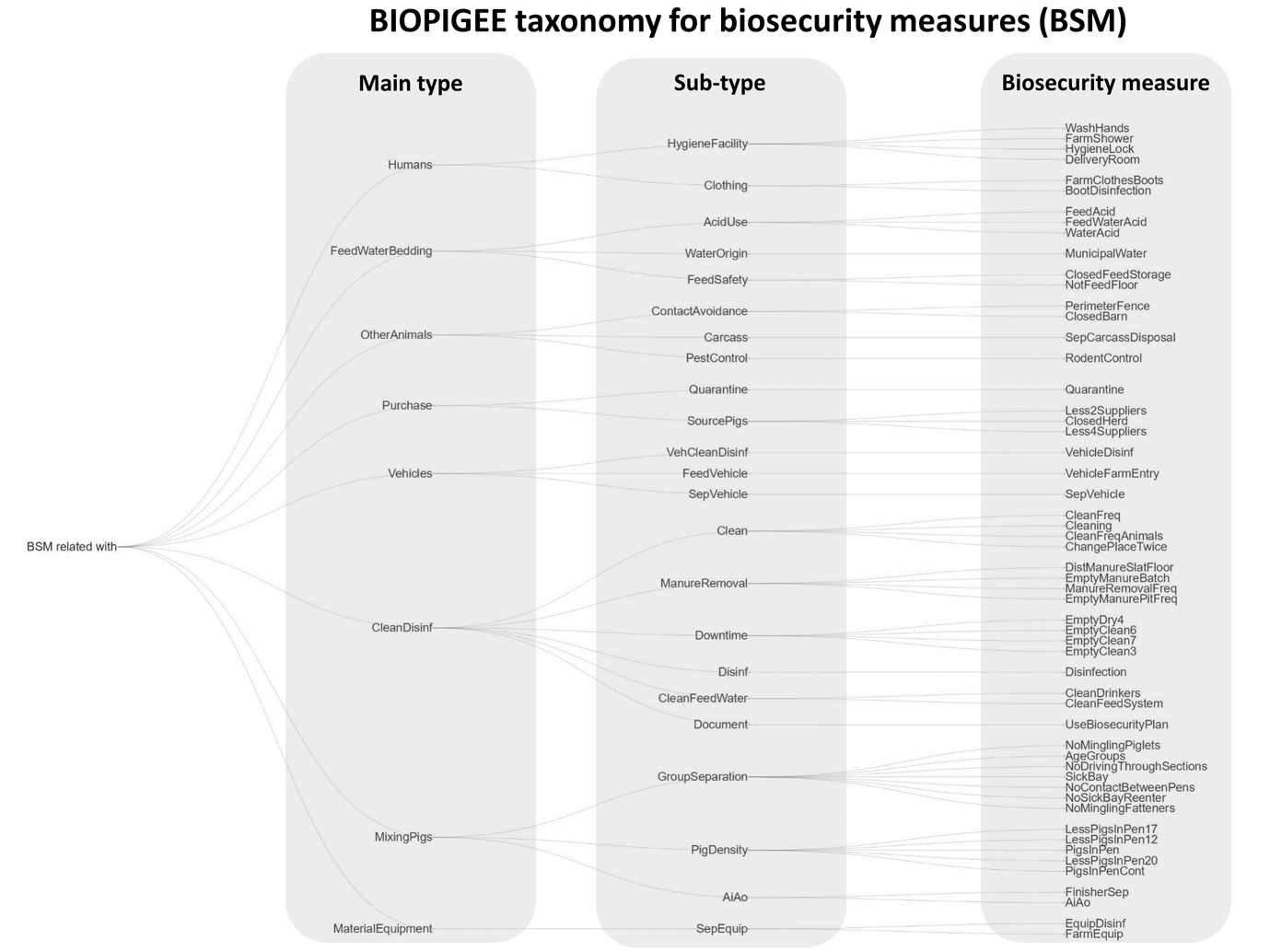


**Supplementary Figure S2:** Number of observations for each main type of biosecurity by pathogen.


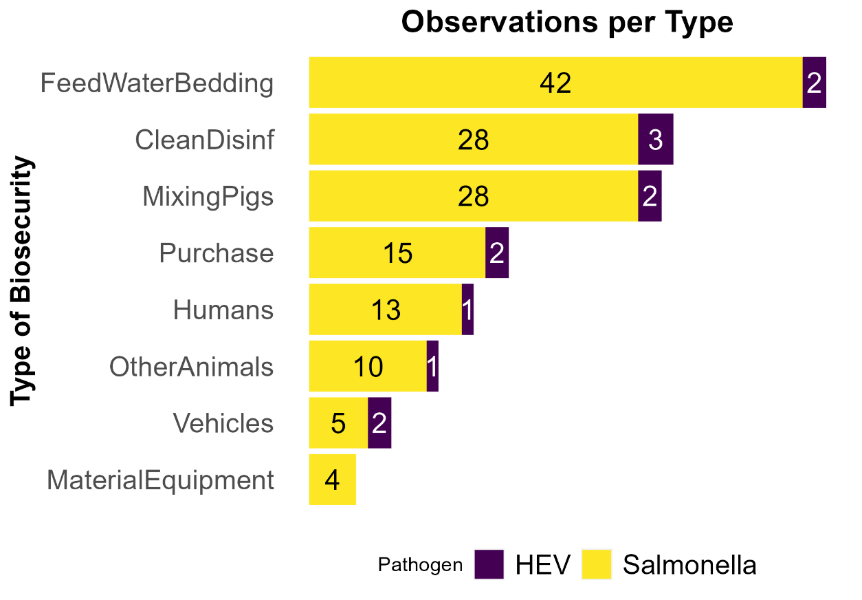


**Supplementary Table S2:** Mask including the signalling questions applied to assess the risk of bias for observational studies modified after Kim et al., J Clin Epidemiol 2013 Vol. 66 Issue 4, Pages 408-14.

|  |  |  |  |  | **Domain-Judgement** |  |
| --- | --- | --- | --- | --- | --- | --- |
| **Signalling questions (SQ)** |  | **SQ - Judgement** |  | High risk | some/unclear risk | Low risk |
| **Selection Bias** | Yes/probably yes | No/probably no | Unclear |  |  |  |
| **Selection of participants**: *(Could the selection of farms/herds/individuals bias the outcome?)* |  |  |  |  |  |  |
| Was the farm randomly selected according to the study question? |  |  |  |  |  |  |
| Was the farm type described/defined? |  |  |  |  |  |  |
| Were the animals, animal groups randomly selected according to the study question? |  |  |  |  |  |  |
| Were the group characteristics defined? |  |  |  |  |  |  |
| Is the subsample representative for the population of interest? |  |  |  |  |  |  |
| **Confounding variables:** (*Inadequate confirmation/consideration of confounding variable*s*?)* |  |  |  |  |  |  |
| Did the study consider relevant confounding factors in the study design, sample/data collection potentially biasing the outcome? |  |  |  |  |  |  |
| Did the study consider relevant confounding factors regarding the effect estimate of the extracted biosecurity measure? |  |  |  |  |  |  |
| **Performance Bias:** | Yes/probably yes | No/probably no | Unclear |  |  |  |
| *Could prior knowledge of study setting or assignment to biosecurity measure bias data collection and outcome?* |  |  |  |  |  |  |
| Did researchers rule out any impact from prior knowledge concurrent interventions/biosecurity measures? |  |  |  |  |  |  |
| Were the samples collected in a blinded manner? |  |  |  |  |  |  |
| **Detection Bias:** | Yes/probably yes | No/probably no | Unclear |  |  |  |
| *Could prior knowledge of the study setting or the assignment to a biosecurity measure bias the measurements/analysis and outcome?* |  |  |  |  |  |  |
| Were the samples analysed in a blinded manner? |  |  |  |  |  |  |
| If measures to ensure blinded analysis are described- was the intended blinding effective |  |  |  |  |  |  |
| Were outcomes assessed/defined using valid and reliable measures, implemented consistently across all study participants? |  |  |  |  |  |  |
| **Attrition Bias:** | Yes/probably yes | No/probably no | Unclear |  |  |  |
| *Could the “exclusion of data” or drop out of individuals during the study or prior to the analysis bias the results?* |  |  |  |  |  |  |
| Was the entire outcome of sample analysis considered in subsequent data analysis? |  |  |  |  |  |  |
| If some data or outcome was not analysed – was this explained transparently? |  |  |  |  |  |  |
| Was missing data due to drop out of study individuals/groups/herds handled appropriately in the analysis? |  |  |  |  |  |  |
| **Reporting bias:** | Yes/probably yes | No/probably no | Unclear |  |  |  |
| *did authors report only certain outcomes to support their main points?* |  |  |  |  |  |  |
| Was the whole outcome (including negative results) of the study reported? |  |  |  |  |  |  |
| If not, did the authors give an appropriate/transparent explanation for it? |  |  |  |  |  |  |
| Were the potential outcomes prespecified by the researchers? |  |  |  |  |  |  |
| If yes, were these prespecified outcomes reported? |  |  |  |  |  |  |
| **Other biases:** | Yes/probably yes | No/probably no | Unclear |  |  |  |
| Does the study include a clear conflict of interest statement? |  |  |  |  |  |  |
| Connection between funding source (esp. industry) and the outcome of the study? |  |  |  |  |  |  |
|  |  |  |  | High risk |  |  |
| Overall Judgement |  |  |  | some/unclear risk |  |  |
|  |  |  |  | Low risk |  |  |

**Supplementary Table S3:** Mask including the signalling questions applied to assess the risk of bias for experimental studies modified after Higgins et al., BMJ 2011;343:d5928.

|  |  |  |  |  | | **Domain-Judgement** | |  |
| --- | --- | --- | --- | --- | --- | --- | --- | --- |
| **Signaling questions (SQ)** | **SQ - Judgement** |  |  | High risk | some/unclear risk | | Low risk | |
| **Selection Bias:** | Yes/probably yes | No/probably no | Unclear |  | |  | |  |
| **Random sequence:** *(concerning the randomization process)* |  |  |  |  | |  | |  |
| Was a randomized sequence generated according to the study question? (e.g., random number table, automated randomization) |  |  |  |  | |  | |  |
| Was the allocation/assignment to an intervention/biosecurity measure reasoned and described? |  |  |  |  | |  | |  |
| Were the group characteristics defined? |  |  |  |  | |  | |  |
| Is the subsample representative for the population of interest? |  |  |  |  | |  | |  |
| **Allocation concealment:** *(concerning allocation to intervention/BSM)* |  |  |  |  | |  | |  |
| Was the concealment (blinding) of allocations before or during the study adequate? |  |  |  |  | |  | |  |
| **Confounding variables:** (*Inadequate confirmation/consideration of confounding variable*s*?)* |  |  |  |  | |  | |  |
| Did the study consider relevant confounding factors in the study design, sample/data collection potentially biasing the outcome? |  |  |  |  | |  | |  |
| Did the study consider relevant confounding factors regarding the effect estimate of the extracted biosecurity measure? |  |  |  |  | |  | |  |
| **Performance Bias:** | Yes/probably yes | No/probably no | Unclear |  | |  | |  |
| *Could knowledge of the study setting, the application of additional biosecurity measures or the assignment to a biosecurity measure bias the data collection/outcome?)* | |  |  |  | |  | |  |
| Did researchers rule out any impact from prior knowledge concurrent interventions/biosecurity measures? |  |  |  |  | |  | |  |
| Were the samples collected in a blinded manner? |  |  |  |  | |  | |  |
| Was intervention applied equally on all groups? |  |  |  |  | |  | |  |
| **Detection Bias:** | Yes/probably yes | No/probably no | Unclear |  | |  | |  |
| *Could prior knowledge of the study setting, the application of a biosecurity measure or the assignment to a biosecurity measure bias the measurements/analysis?)* |  |  |  |  | |  | |  |
| Were the samples *analyzed* in a blinded manner (in the lab/other analysis)? |  |  |  |  | |  | |  |
| If measures to ensure blinded analysis are described- was the intended blinding effective |  |  |  |  | |  | |  |
| Were outcomes assessed/defined using valid and reliable measures, implemented consistently across all study participants? |  |  |  |  | |  | |  |
| **Attrition Bias:** | Yes/probably yes | No/probably no | Unclear |  | |  | |  |
| *Could the “exclusion of data” during the study or prior to the analysis bias the results?* |  |  |  |  | |  | |  |
| Was the total/all outcome of sample analysis considered in subsequent data analysis? |  |  |  |  | |  | |  |
| If some data or outcome was not analyzed – was this explained transparently? |  |  |  |  | |  | |  |
| Was missing data due to drop out of study individuals/groups/herds handled appropriately in the analysis? |  |  |  |  | |  | |  |
| **Reporting bias:** | Yes/probably yes | No/probably no | Unclear |  | |  | |  |
| *did authors report only certain outcomes to support their main points; selective reporting?* |  |  |  |  | |  | |  |
| Was the whole outcome (including negative results) of the study reported? |  |  |  |  | |  | |  |
| If not, did the authors give an appropriate/transparent explanation for it? |  |  |  |  | |  | |  |
| Were the potential outcomes pre-specified by the researchers? |  |  |  |  | |  | |  |
| If yes were these pre-specified outcomes reported? |  |  |  |  | |  | |  |
| **Other biases:** | Yes/probably yes | No/probably no | Unclear |  | |  | |  |
| Does the study include a clear conflict of interest statement? |  |  |  |  | |  | |  |
| Is there a possible connection between the funding source (esp. industries) and the outcome of the study? |  |  |  |  | |  | |  |
| Was there any other impact on the analysis (categorization) |  |  |  |  | |  | |  |
|  |  |  |  | High risk | |  | |  |
| Overall judgement |  |  |  | some/unclear risk | |  | |  |
|  |  |  |  | Low risk | |  | |  |

**Supplementary Table 4**: Risk of bias ratings per bias domain for all studies in the final selection stage (n =32; observational studies = 27, experimental studies = 5). Numbers are shown in percentages.

|  | **low** | **moderate** | **high** |
| --- | --- | --- | --- |
| **Selection Bias** | 3.1 | 9.4 | 87.5 |
| **Performance Bias** | 3.1 | 21.9 | 75.0 |
| **Detection Bias** | 6.3 | 46.9 | 46.9 |
| **Attrition Bias** | 40.6 | 25.0 | 34.4 |
| **Reporting Bias** | 40.6 | 18.8 | 40.6 |
| **Other Bias** | 21.9 | 18.8 | 59.4 |
| **Overall risk** |  |  | 100.0 |

**Supplementary Table S5**: Risk of Bias rating per risk of bias domain and study type (observational n = 27, experimental n = 5) for all studies included in the final selection for data extraction.

|  | **Selection Bias** | **Performance Bias** | **Detection Bias** | **Attrition Bias** | **Reporting Bias** | **Other bias** | **Overall risk** | **study type** |
| --- | --- | --- | --- | --- | --- | --- | --- | --- |
| Walachowski et al_2014 | high | moderate | low | high | high | low | high | Observ. |
| Meyer et al_2005 | high | high | high | high | high | high | high | Observ. |
| Altrock et al_2000 | high | high | moderate | low | low | high | high | Observ. |
| Beloeil et al_2004 | high | moderate | high | low | low | high | high | Observ. |
| van der Wolf et al_2001a | high | high | high | moderate | low | high | high | Exper. |
| Smith et al_2018 | moderate | high | high | moderate | low | low | high | Observ. |
| Stege et al_2001 | moderate | high | high | high | moderate | high | high | Observ. |
| Lopez-Lopez et al_2018 | high | high | high | low | high | low | high | Observ. |
| Davies et al_1997a | high | high | moderate | low | high | high | high | Observ. |
| Martelli et al_2017 | high | high | high | high | high | high | high | Observ. |
| Correia-Gomes et al_2012 | low | moderate | moderate | low | low | moderate | high | Observ. |
| Creus et al_2007 | high | moderate | moderate | low | low | moderate | high | Exper. |
| Davies et al_1997b | high | high | moderate | high | high | high | high | Observ. |
| Rajić et al_2007 | moderate | moderate | moderate | high | low | moderate | high | Observ. |
| Poljak et al_2007 | high | moderate | moderate | low | low | low | high | Observ. |
| Willamil et al_2011 | high | high | moderate | low | low | moderate | high | Exper. |
| Fablet et al_2003 | high | high | high | high | high | high | high | Observ. |
| Argüello et al_2013 | high | moderate | moderate | low | low | low | high | Exper. |
| Cardinale et al_2010 | high | high | high | moderate | moderate | high | high | Observ. |
| Cevallos-Almeida et al_2019 | high | high | high | moderate | moderate | moderate | high | Observ. |
| García-Feliz et al_2009 | high | high | high | high | moderate | high | high | Observ. |
| Ruggeri et al_2018 | high | high | high | moderate | low | high | high | Exper. |
| San Román et al_2018 | high | high | high | moderate | moderate | moderate | high | Observ. |
| Caruso et al_2017 | high | high | moderate | low | moderate | low | high | Observ. |
| Pavia et al_2021 | high | high | high | moderate | high | low | high | Observ. |
| Dors et al_2015 | high | high | moderate | low | low | high | high | Observ. |
| EFSA_2011 | high | high | moderate | low | low | high | high | Observ. |
| Twomey et al_2010 | high | high | high | high | high | high | high | Observ. |
| van der Wolf et al_2001b | high | low | low | moderate | high | high | high | Observ. |
| Vico et al_2011 | high | high | moderate | high | high | high | high | Observ. |
| Wilkins et al_2010 | high | high | moderate | high | high | high | high | Observ. |
| Gotter et al_2012 | high | high | moderate | low | high | high | high | Observ. |

**Supplementary Figure S3**: Forest plots of random-effects meta-analysis (with restricted maximum likelihood estimator for the amount of heterogeneity) for different biosecurity measures (BSMs): all-in all-out production with all observations (A1), all-in all-out production without the observation with extremely wide confidence interval (A2), disinfection (B), feed acidification (C) and water acidification (D). These meta-analyses are for *Salmonella* spp*.* and ignore the production stage which the observations are considering. Each observation is encoded with internal BIOPIGEE designations for the article ID (Art Id), the observation ID (Obs Id) and the production stage. Where available the number of *Salmonella* spp*.* positive (‘Salm+’) and negative (‘Salm-‘) samples for the BSM and the control condition as stated in the corresponding publication are provided. Each square indicates the odds ratio (OR) for one observation, with the size of the square indicating the weight with which that observation contributed to the summary value of the meta-analysis. The whiskers on the squares indicate the 95% confidence intervals (CI) for each observation. The diamond at the bottom indicates the summary value of the meta-analysis for the OR of each BSM.


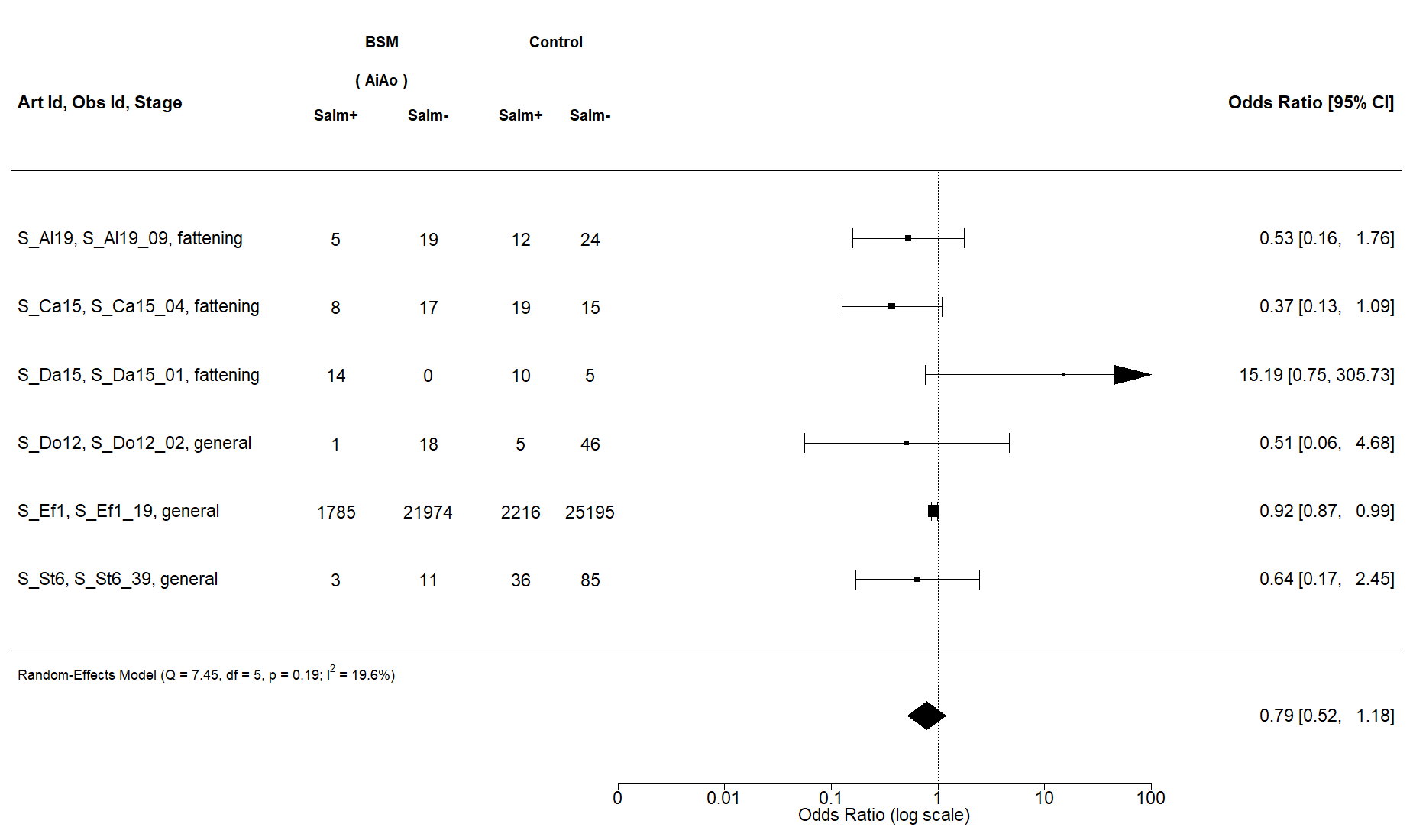


A1. Forest plots of random-effects meta-analysis (with restricted maximum likelihood estimator for the amount of heterogeneity) for the BSM “all-in all-out production (AiAo)” for Salmonella spp. and ignoring the production stage. Note the extremely wide confidence interval from observation “S_Da15_01”.

#####
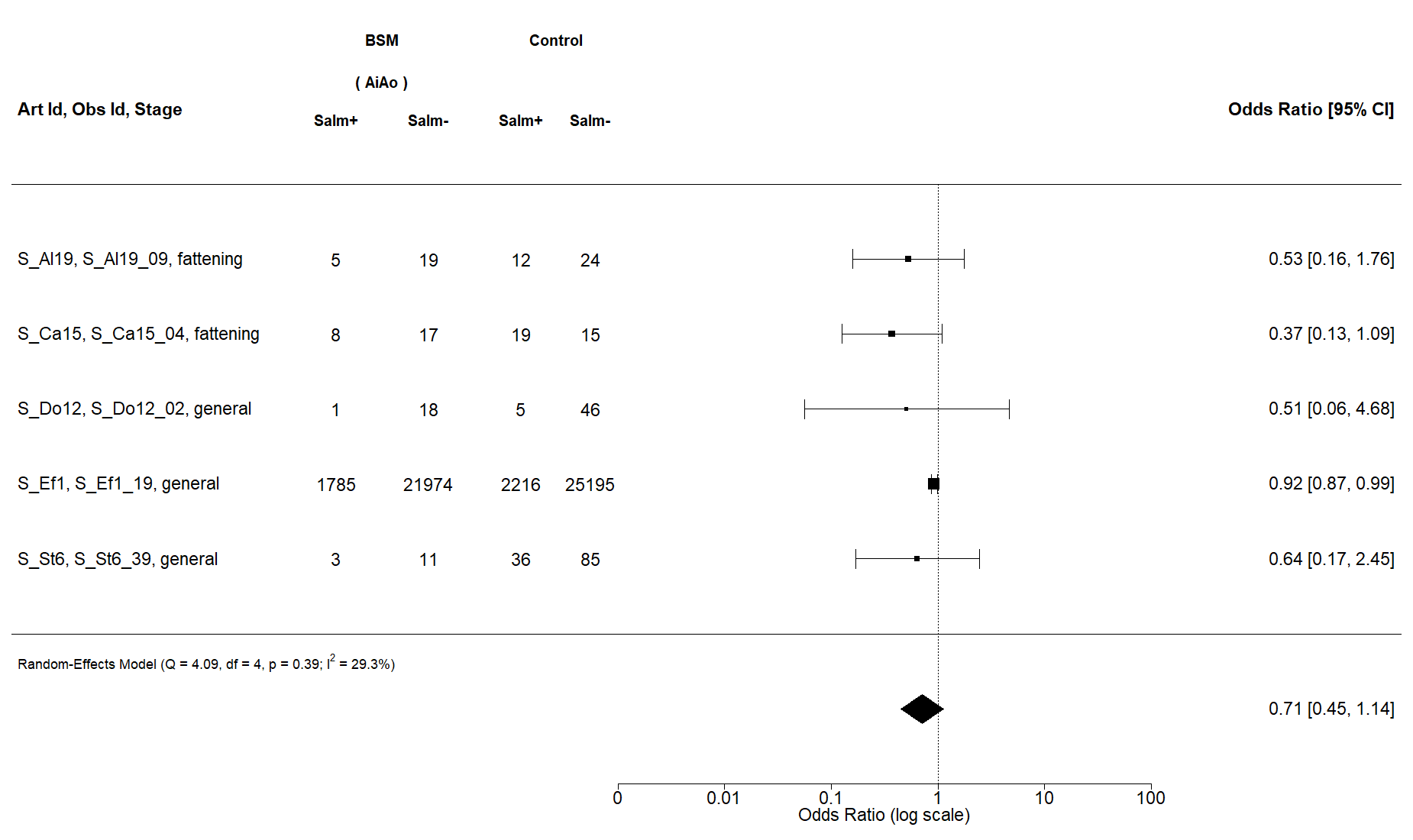


A2. Forest plots of random-effects meta-analysis (with restricted maximum likelihood estimator for the amount of heterogeneity) for the BSM “all-in all-out production (AiAo)” for Salmonella spp. and ignoring the production stage. Here the observation “S_Da15_01” with the extreme wide confidence interval from Supplementary Figure 3A1 has been removed.


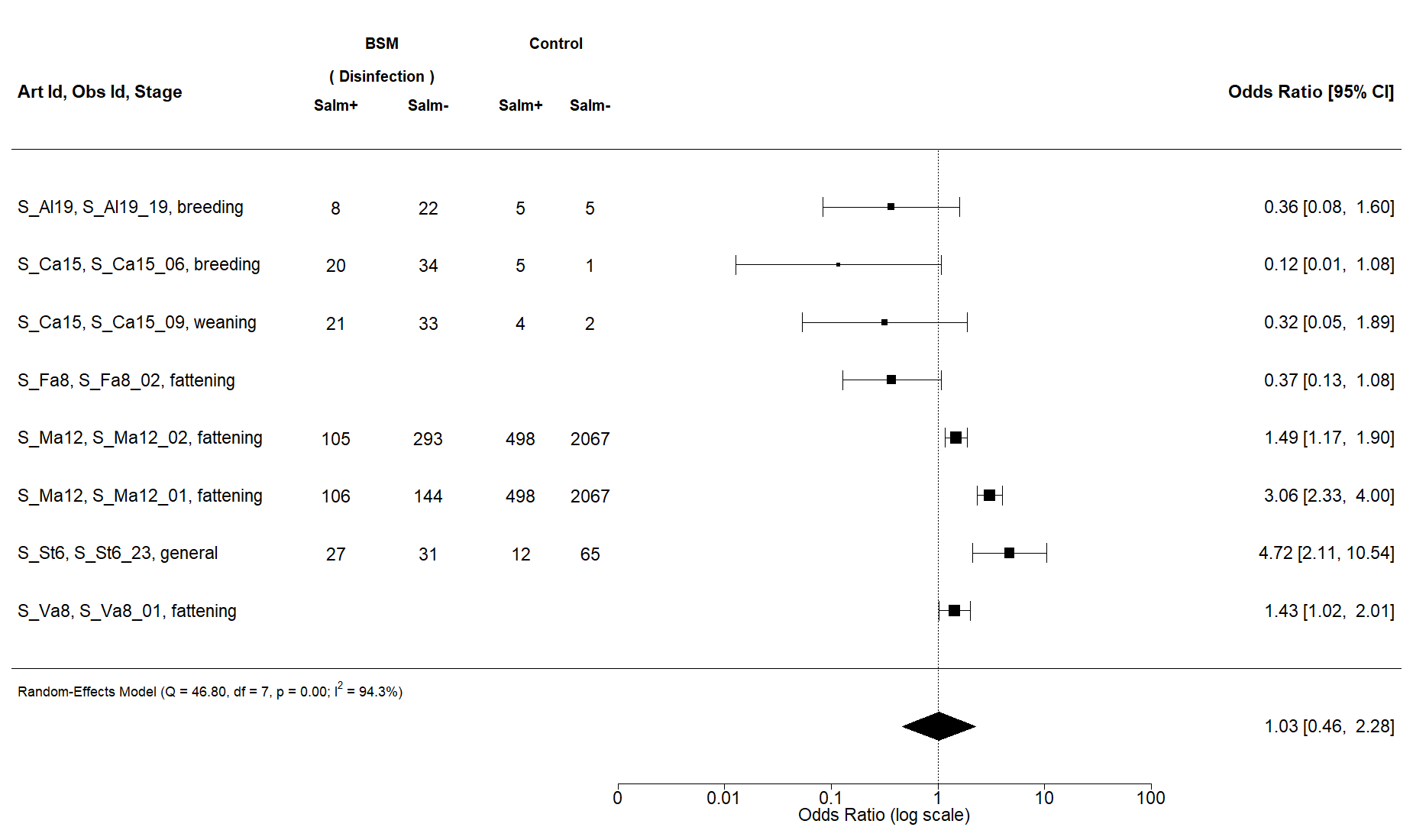


B. Forest plots of random-effects meta-analysis (with restricted maximum likelihood estimator for the amount of heterogeneity) for the BSM “disinfection” for Salmonella spp. and ignoring the production stage.


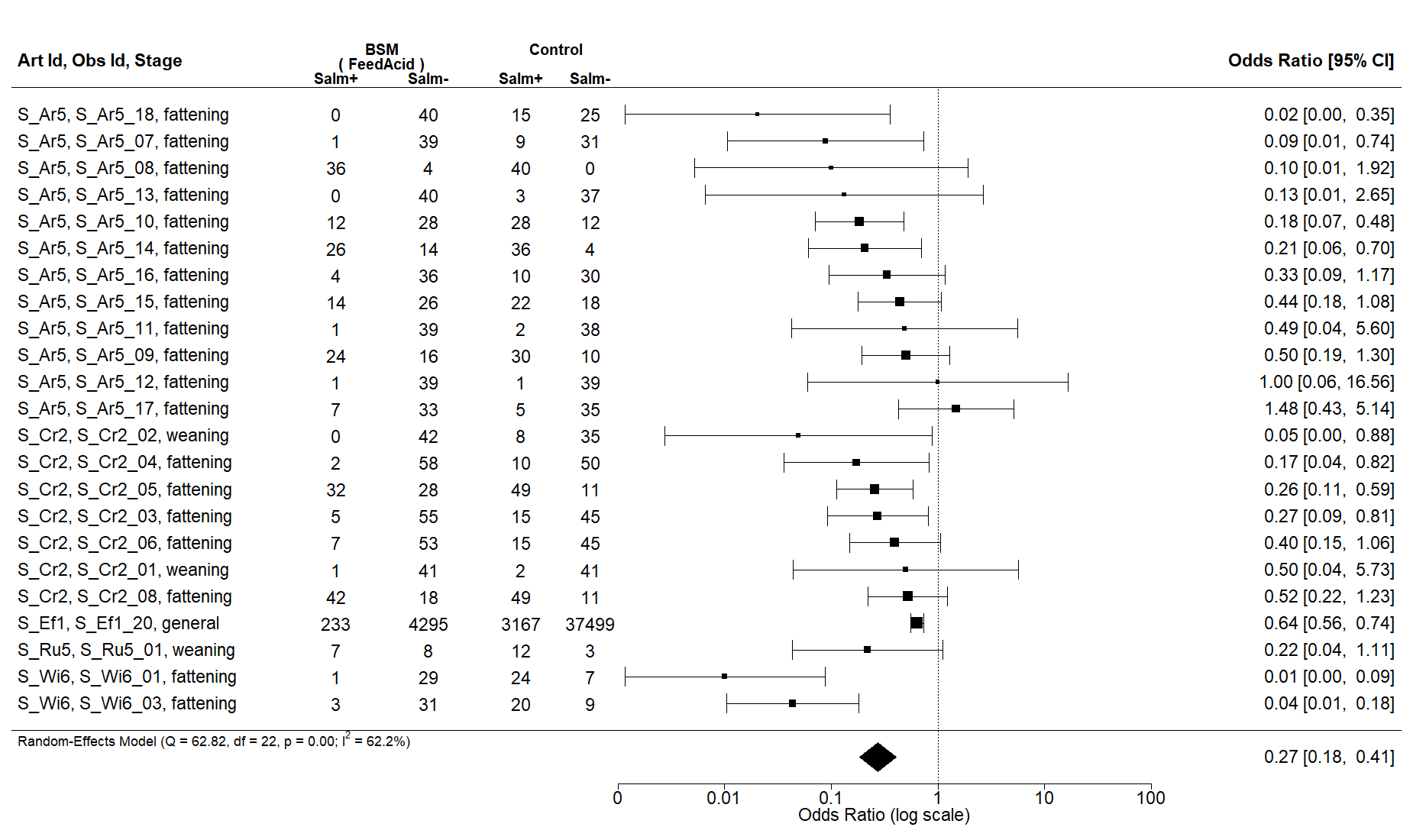


C. Forest plots of random-effects meta-analysis (with restricted maximum likelihood estimator for the amount of heterogeneity) for the BSM “feed acidification” for Salmonella spp. and ignoring the production stage.


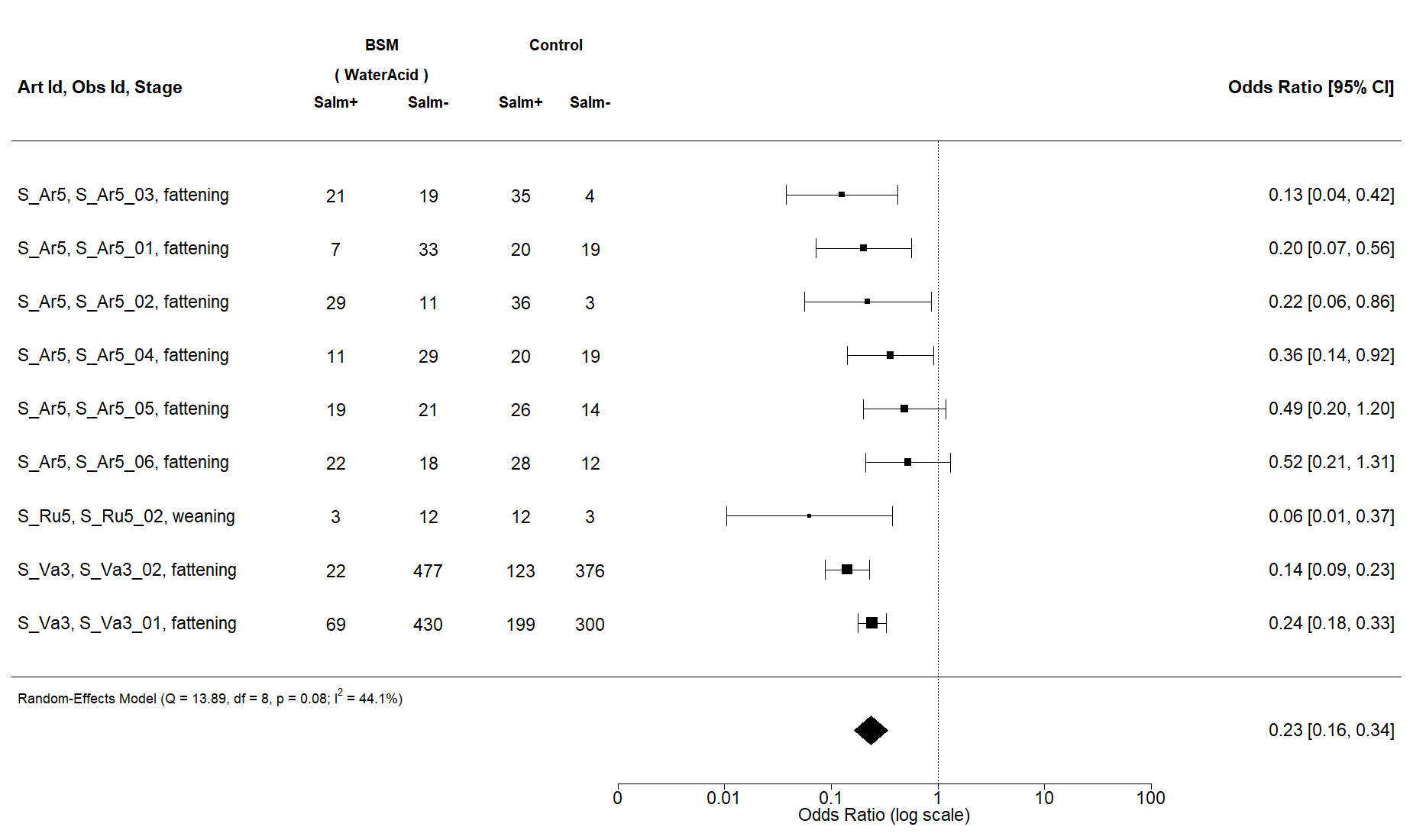


D. Forest plots of random-effects meta-analysis (with restricted maximum likelihood estimator for the amount of heterogeneity) for the BSM “ water acidification” for Salmonella spp. and ignoring the production stage.

**Supplementary Figure S4:** Contour-enhanced funnel plot for observations included in the meta-analysis on the mitigating effect of feed acidification (A) and water acidification (B) on *Salmonella* spp. infection ignoring the stage in pig production. Each point is an observation from the literature. The shaded areas help identify whether observations lie above or under statistical significance. The funnel with dotted lines indicates how the observations are scattered around the summary OR.


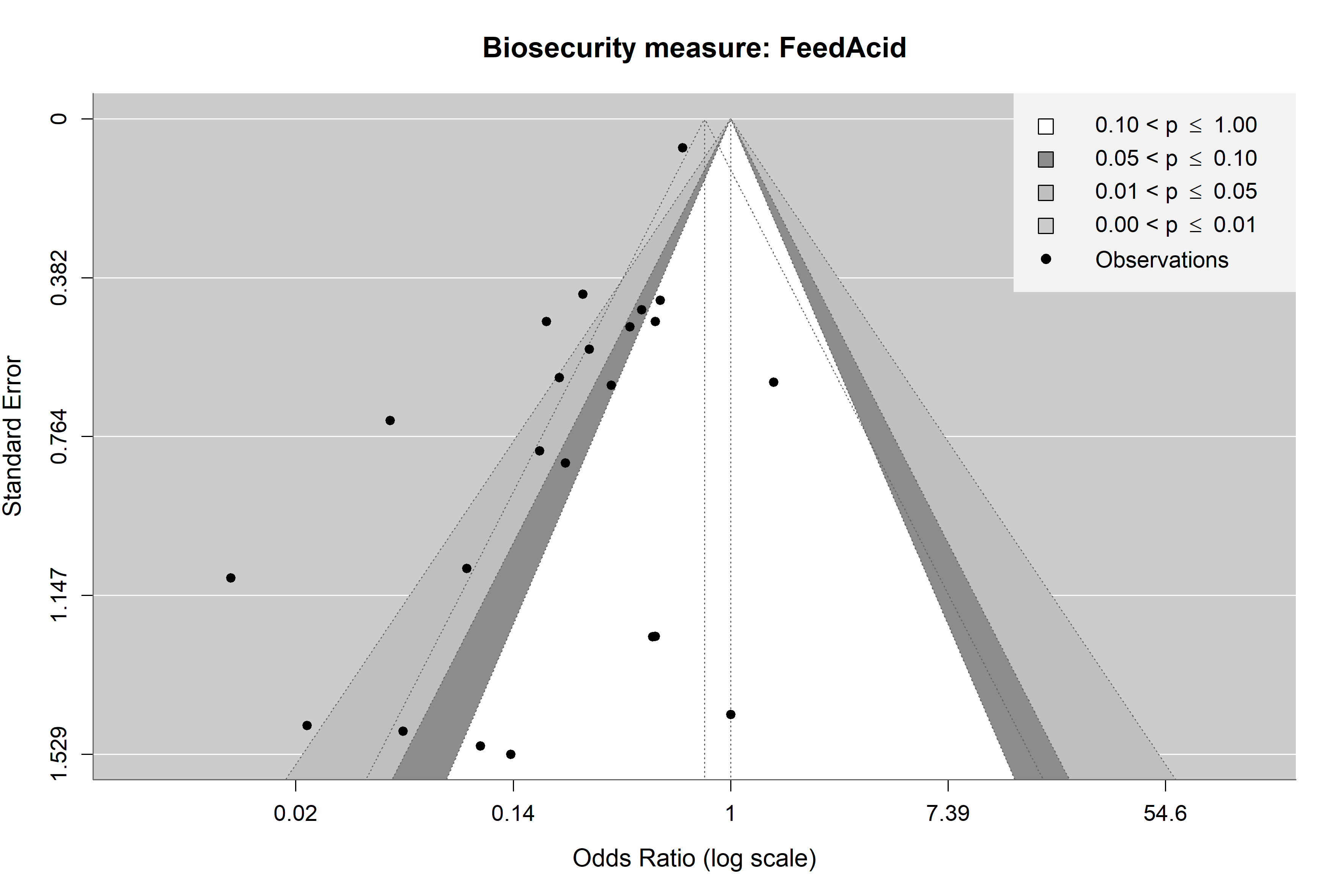
*A. For acidification of feed the dotted funnel centres at an OR of 0.27. Egger’s test provided evidence for funnel plot asymmetry and hence publication bias (p<0.0001).*


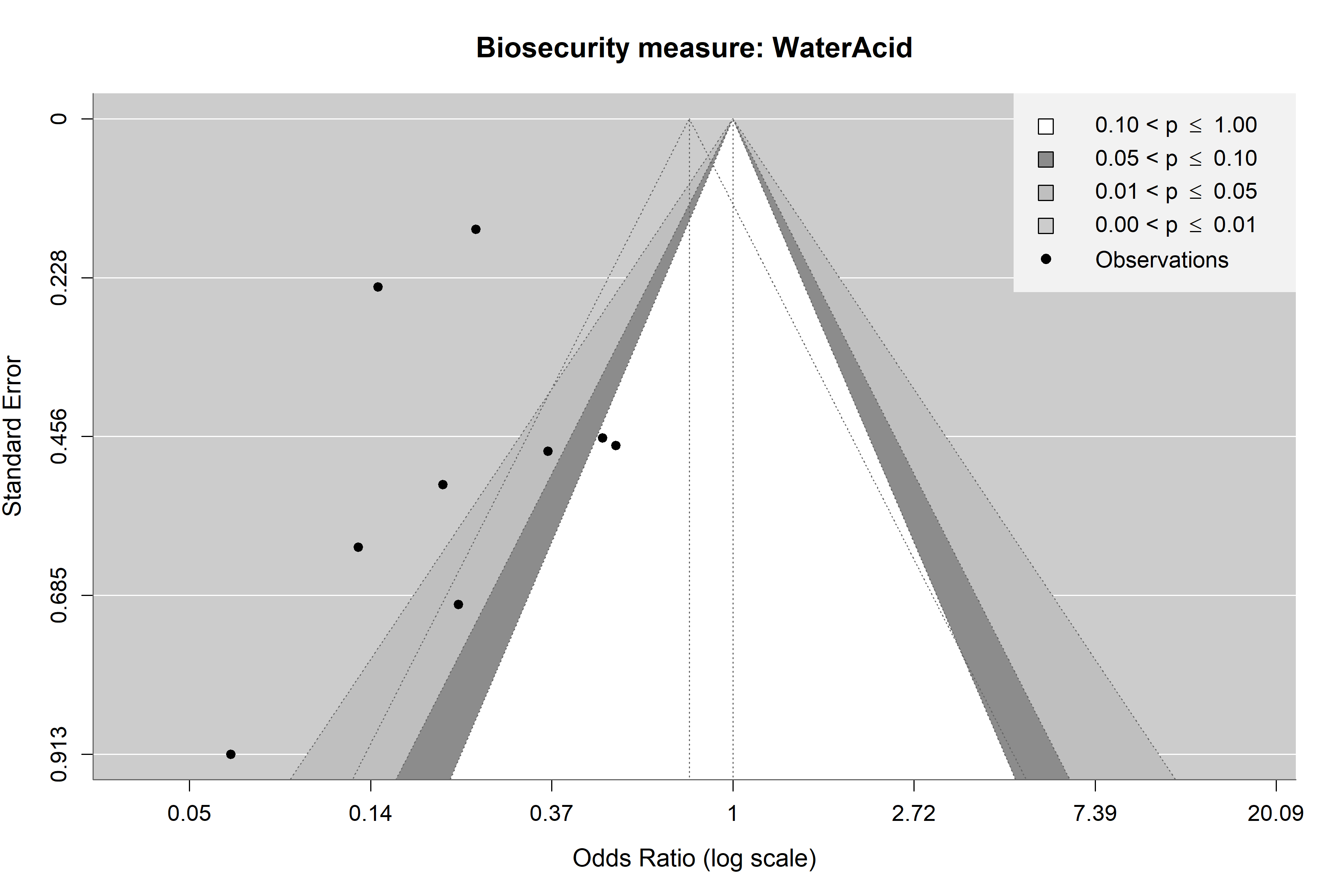
*B. For acidification of water the dotted funnel centres at an OR of 0.23. Egger’s test did not provide evidence for funnel plot asymmetry and hence publication bias (p=0.9508).*

**Supplementary Figure S5: Diagnostic plot for sensitivity analysis** for observations included in the meta-analysis on the mitigating effect of feed acidification (A) and water acidification (B) on *Salmonella* spp. infection ignoring the stage in pig production.


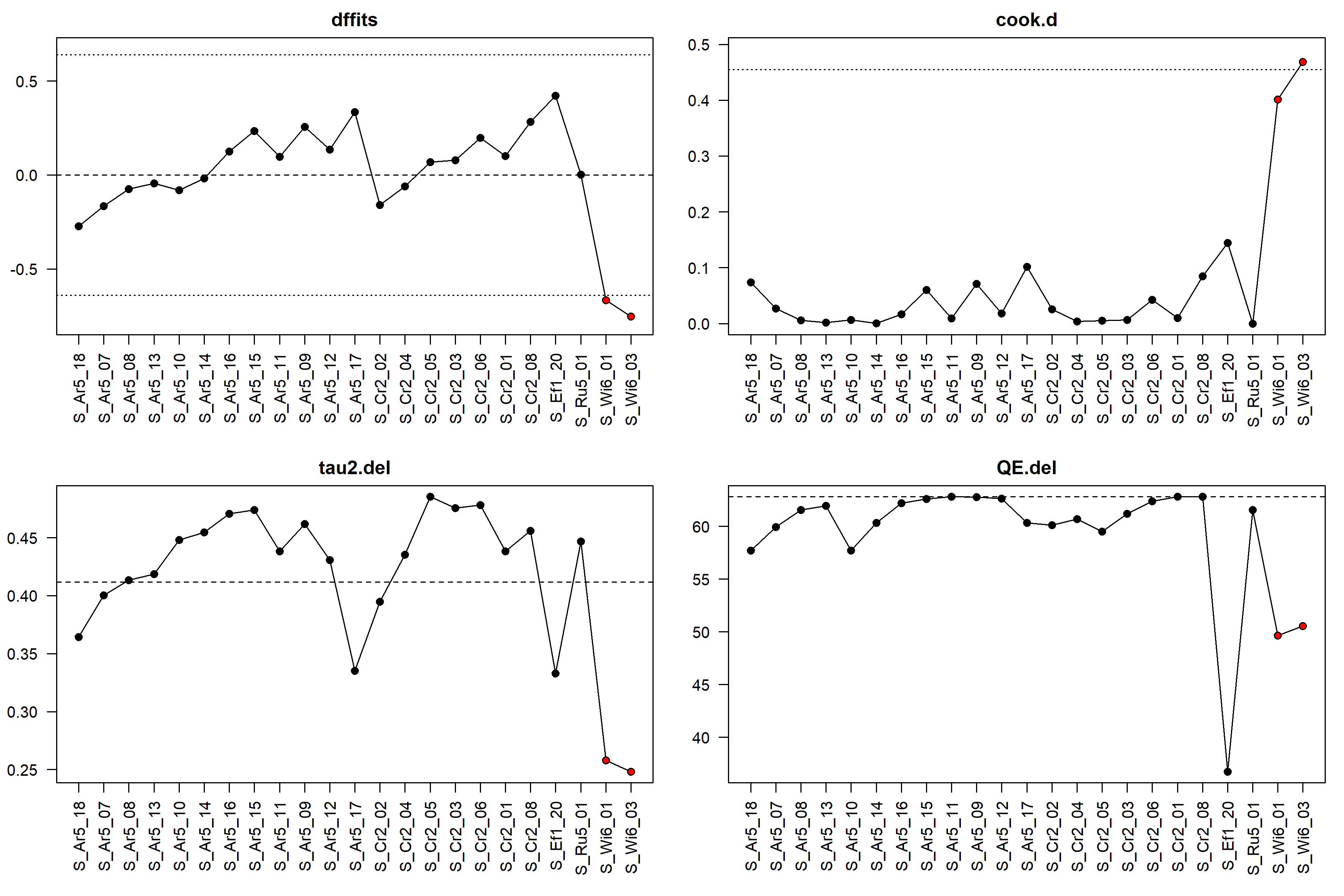


*A. For acidification of feed two observation are considered influential on the outcome of the meta-analysis: S_Wi6_01 and S_Wi6_03. Both lead to dffits values below the lower dffits cut-off but only S_Wi6_03 lead to an excess of the cook.d cut-off. Leaving each of these two observations out reduce the heterogeneity of the meta-analysis.*


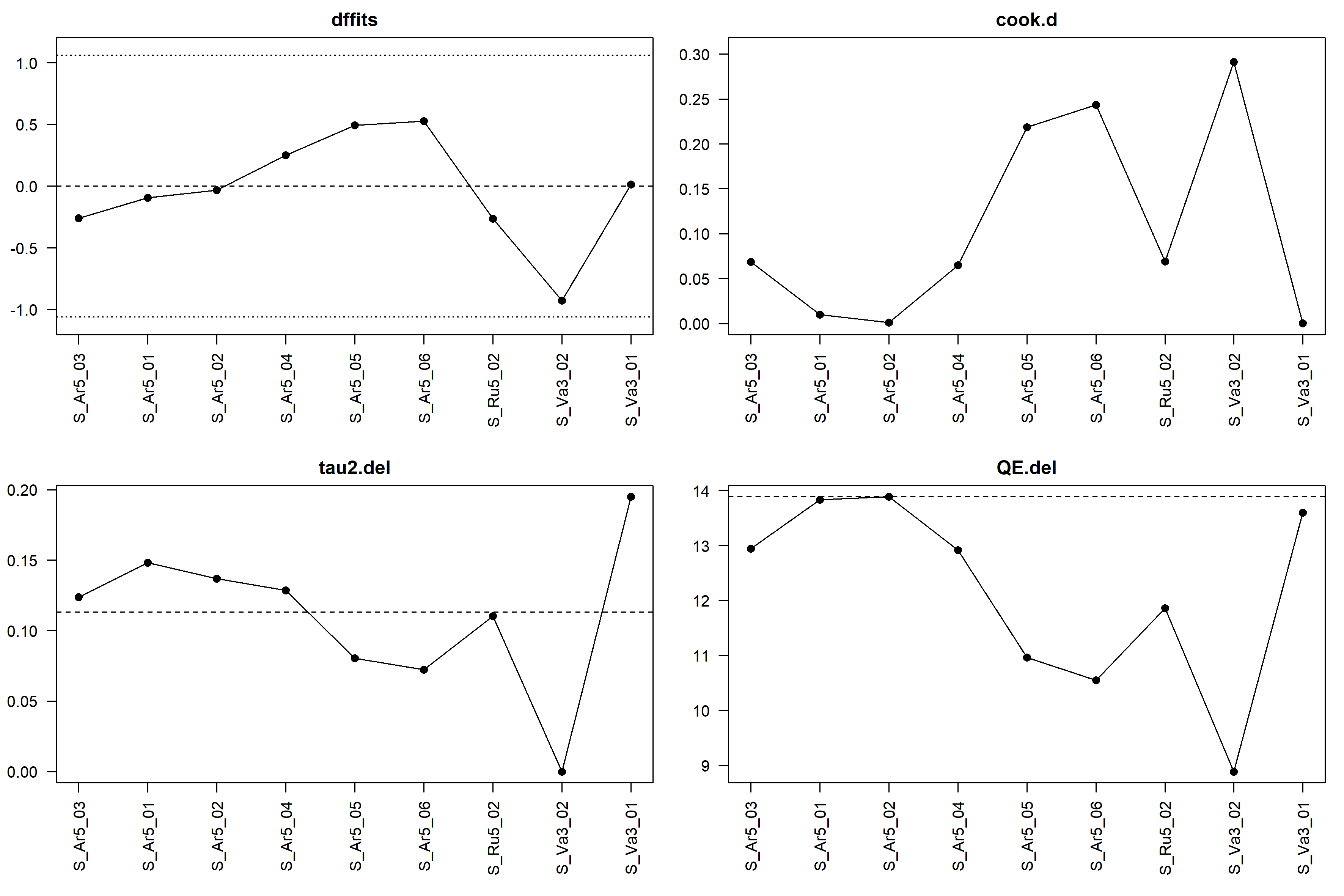


*B. For acidification of water no observation are considered influential on the outcome of the meta-analysis.*

**Supplementary Table S6**: The 32 publications included in the analysis have BIOPIGEE internal short hands listed in column ‘Article ID’. This table furthermore provides information on the article title, author, year of publication, country where the study was undertaken, the study design, the pathogen and farm type(s) under consideration.

| Article ID | Article title | Author | Year | Country | Study Design | Pathogen | Farm Type |
| --- | --- | --- | --- | --- | --- | --- | --- |
| S_Ar5 | Effect of the Addition of Organic Acids in Drinking Water or Feed During Part of the Finishing Period on the Prevalence of *Salmonella* in Finishing Pigs | Arguello, H., Carvajal, A., Costillas, S., Rubio, P. | 2013 | ES | experimental | *Salmonella* | farrow-to-finish |
| S_Ef1 | Analysis of the baseline survey on the prevalence of *Salmonella* in holdings with breeding pigs, in the EU, 2008 Part B: factors associated with *Salmonella* pen positivity European Food Safety Authority | Authority, European Food Safety | 2011 | EU | observational, cross-sectional | *Salmonella* | mixed |
| S_Be27 | Risk factors for *Salmonella* *enterica* subsp enterica shedding by market-age pigs in French farrow-to-finish herds | Beloeil, P. A., Fravalo, P., Fablet, C., Jolly, J. P., Eveno, E., Hascoet, Y., Chauvin, C., Salvat, G., Madec, F. | 2004 | FR | observational, cross-sectional | *Salmonella* | farrow-to-finish |
| S_Ca15 | *Salmonella* in fattening pigs in Reunion Island: Herd prevalence and risk factors for infection | Cardinale, E., Maeder, S., Porphyre, V., Debin, M. | 2010 | FR | observational, cross-sectional | *Salmonella* | farrow-to-finish |
| H_Ca3 | Hepatitis E Virus: A Cross-Sectional Serological and Virological Study in Pigs and Humans at Zoonotic Risk within a High-Density Pig Farming Area | Caruso, C., Peletto, S., Rosamilia, A., Modesto, P., Chiavacci, L., Sona, B., Balsamelli, F., Ghisetti, V., Acutis, P. L., Pezzoni, G., Brocchi, E., Vitale, N., Masoero, L. | 2017 | IT | observational, cross-sectional | HEV | mixed |
| S_Ce5 | Longitudinal study describing time to *Salmonella* seroconversion in piglets on three farrow-to-finish farms | Cevallos-Almeida, M., Fablet, C., Houdayer, C., Dorenlor, V., Eono, F., Denis, M., Kerouanton, A. | 2019 | FR | observational, longitudinal | *Salmonella* | farrow-to-finish |
| S_Co13 | Assessing risk profiles for *Salmonella* serotypes in breeding pig operations in Portugal using a Bayesian hierarchical model | Correia-Gomes, C., Economou, T., Mendonca, D., Vieira-Pinto, M., Niza-Ribeiro, J. | 2012 | PT | case-control | *Salmonella* | breeding |
| S_Cr2 | Effect of acidified feed on the prevalence of *Salmonella* in market-age pigs | Creus, E., Perez, J. F., Peralta, B., Baucells, F., Mateu, E. | 2007 | ES | experimental | *Salmonella* | farrow-to-finish,fattening |
| S_Da16 | Risk of shedding *Salmonella* organisms by market-age hogs in a barn with open-flush gutters | Davies, P. R., Morrow, W. E. M., Jones, F. T., Deen, J., FedorkaCray, P. J., Gray, J. T. | 1997 | US | Observational, case-control | *Salmonella* | finishing |
| S_Da15 | Prevalence of *Salmonella* in finishing swine raised in different production systems in North Carolina, USA | Davies, P. R., Morrow, W. E. M., Jones, F. T., Deen, J., FedorkaCray, P. J., Harris, I. T. | 1997 | US | observational, cross-sectional | *Salmonella* | farrow-to-finish |
| S_Do12 | Prevalence and risk factors for *Lawsonia intracellularis*, *Brachyspira hyodysenteriae* and *Salmonella* spp. in finishing pigs in Polish farrow-to-finish swine herds | Dors, A., Pomorska-Mol, M., Czyzewska, E., Wasyl, D., Pejsak, Z. | 2015 | PL | observational, cross-sectional | *Salmonella* | farrow-to-finish |
| S_Fa8 | Recherche des facteurs de risque de l’excrétion de *Salmonella* *enterica* par les porcs en croissance. Enquête épidémiologique analytique en élevage naisseur-engraisseur | Fablet, Christelle, Fravalo, Philippe, Jolly, Jean-Pierre, Eveno, Eric, Madec, François, Beloeil, Pierre-Alexandre | 2003 | FR | observational, cross-sectional | *Salmonella* | farrow-to-finish |
| S_Ga5 | Herd-level risk factors for faecal shedding of *Salmonella enterica* in Spanish fattening pigs | Garcia-Feliz, C., Carvajal, A., Collazos, J. A., Rubio, P. | 2009 | ES | observational, cross-sectional | *Salmonella* | mixed |
| S_Go12 | Main risk factors for *Salmonella*-infections in pigs in north-western Germany | Gotter, V., Klein, G., Koesters, S., Kreienbrock, L., Blaha, T., Campe, A. | 2012 | DE | Observational, case-control | *Salmonella* | NA |
| H_Lo3 | Risk factors associated with hepatitis E virus in pigs from different production systems | Lopez-Lopez, P., Risalde, M. D., Frias, M., Garcia-Bocanegra, I., Brieva, T., Caballero-Gomez, J., Camacho, A., Fernandez-Molera, V., Machuca, I., Gomez-Villamandos, J. C., Rivero, A., Rivero-Juarez, A. | 2018 | ES | observational, cross-sectional | HEV | mixed |
| S_Ma12 | Evaluation of an enhanced cleaning and disinfection protocol in *Salmonella* contaminated pig holdings in the United Kingdom | Martelli, F., Lambert, M., Butt, P., Cheney, T., Tatone, F. A., Callaby, R., Rabie, A., Gosling, R. J., Fordon, S., Crocker, G., Davies, R. H., Smith, R. P. | 2017 | GB | observational | *Salmonella* | finishing |
| S_Me20 | *Salmonella* seroprevalence in different pig production systems | Meyer, C., Beilage, G. E., Krieter, J. | 2005 | DE | observational, cross-sectional | *Salmonella* | mixed |
| H_Pa5 | Seroprevalence and phylogenetic characterization of hepatitis E virus in pig farms in Southern Italy | Pavia, G., Gioffre, A., Pirolo, M., Visaggio, D., Clausi, M. T., Gherardi, M., Samele, P., Ciambrone, L., Di Natale, R., Spatari, G., Visca, P., Casalinuovo, F. | 2021 | IT | observational, cross-sectional | HEV | mixed |
| S_Po5 | Multilevel analysis of risk factors for Salmonella shedding in Ontario finishing pigs | Poljak, Z., Dewey, C. E., Friendship, R. M., Martin, S. W., Christensen, J. | 2008 | CA | observational, cross-sectional | *Salmonella* | finishing |
| S_Ra15 | Farm-level risk factors for the presence of *Salmonella* in 89 Alberta swine-finishing barns | Rajic, A., O'Connor, B. P., Deckert, A. E., Keenliside, J., McFall, M. E., Reid-Smith, R. J., Dewey, C. E., McEwen, S. A. | 2007 | CA | observational, longitudinal study | *Salmonella* | finishing |
| S_Ru5 | The synergistic effect of organic acids, phytochemicals and a permeabilizing complex reduces *Salmonella* Typhimurium 1,4,[5],12:i-shedding in pigs (vol 42, pg 209, 2018) | Ruggeri, J., Foresti, F., Pavesi, R., Terrini, A., Giudici, F., Padoan, D., Corradi, A., Ossiprandi, M. C., Pasquali, P., Alborali, G. L. | 2018 | IT | experimental | *Salmonella* | weaning |
| S_Sa10 | Relationship between *Salmonella* infection, shedding and serology in fattening pigs in low-moderate prevalence areas | San Roman, B., Garrido, V., Sanchez, S., Martinez-Ballesteros, I., Garaizar, J., Mainar-Jaime, R. C., Migura-Garcia, L., Grillo, M. J. | 2018 | ES | observational, cross-sectional | *Salmonella* | fattening |
| S_Sm6 | How do pig farms maintain low *Salmonella* prevalence: a case-control study | Smith, R. P., Andres, V., Cheney, T. E., Martelli, F., Gosling, R., Marier, E., Rabie, A., Gilson, D., Davies, R. H. | 2018 | GB | experimental | *Salmonella* | mixed |
| S_St6 | Data-quality issues and alternative variable-screening methods in a questionnaire-based study on subclinical *Salmonella enterica* infection in Danish pig herds | Stege, H., Christensen, J., Nielsen, J. P., Willeberg, P. | 2001 | DK | experimental | *Salmonella* | mixed |
| S_Tw1 | Association between biosecurity and *Salmonella* species prevalence on English pig farms | Twomey, D. F., Miller, A. J., Snow, L. C., Armstrong, J. D., Davies, R. H., Williamson, S. M., Featherstone, C. A., Reichel, R., Cook, A. J. C. | 2010 | GB | observational, cross-sectional | *Salmonella* | mixed |
| S_Va3 | Administration of acidified drinking water to finishing pigs in order to prevent *Salmonella* infections | van der Wolf, P. J., van Schie, F. W., Elbers, A. R. W., Engel, B., van der Heijden, H. M. J. F., Hunneman, W. A., Tielen, M. J. M. | 2001 | NL | experimental | *Salmonella* | finishing |
| S_Va8 | Herd level husbandry factors associated with the serological *Salmonella* prevalence in finishing pig herds in The Netherlands | van der Wolf, P. J., Wolbers, W. B., Elbers, A. R. W., van der Heijden, H. M. J. F., Koppen, J. M. C. C., Hunneman, W. A., van Schie, F. W., Tielen, M. J. M. | 2001 | NL | observational, cross-sectional | *Salmonella* | farrow-to-finish |
| S_Vi9 | Salmonellosis in Finishing Pigs in Spain: Prevalence, Antimicrobial Agent Susceptibilities, and Risk Factor Analysis | Vico, J. P., Rol, I., Garrido, V., Roman, B. S., Grillo, M. J., Mainar-Jaime, R. C. | 2011 | ES | observational, cross-sectional | *Salmonella* | mixed |
| S_Al19 | Results of the German Investigation in the EU-Project "*Salmonella* in Pork (Salinpork)" - Part 1: Investigations in the farms | Von Altrock, A., Schutte, A., Hildebrandt, G. | 2000 | DE | observational, cross-sectional | *Salmonella* | mixed,breeding |
| H_Wa5 | Risk factors associated with the presence of hepatitis E virus in livers and seroprevalence in slaughter-age pigs: a retrospective study of 90 swine farms in France | Walachowski, S., Dorenlor, V., Lefevre, J., Lunazzi, A., Eono, F., Merbah, T., Eveno, E., Pavio, N., Rose, N. | 2014 | FR | observational, cross-sectional | HEV | NA |
| S_Wi2 | Distribution of *Salmonella* serovars in breeding, nursery, and grow-to-finish pigs, and risk factors for shedding in ten farrow-to-finish swine farms in Alberta and Saskatchewan | Wilkins, W., Rajic, A., Waldner, C., McFall, M., Chow, E., Muckle, A., Rosengren, L. | 2010 | CA | observational, cross-sectional | *Salmonella* | farrow-to-finish |
| S_Wi6 | Effect of a microencapsulated feed additive of lactic and formic acid on the prevalence of *Salmonella* in pigs arriving at the abattoir | Willamil, J., Creus, E., Perez, J. F., Mateu, E., Martin-Orue, S. M. | 2011 | ES | experimental | *Salmonella* | farrow-to-finish |
